# Proteomic analysis of free-living *Bradyrhizobium diazoefficiens*: highlighting potential determinants of a successful symbiosis

**DOI:** 10.1186/1471-2164-15-643

**Published:** 2014-08-03

**Authors:** Douglas Fabiano Gomes, Jesiane Stefânia da Silva Batista, Amanda Alves Paiva Rolla, Luciano Paulino da Silva, Carlos Bloch, Lygia Vitoria Galli-Terasawa, Mariangela Hungria

**Affiliations:** Embrapa Soja, Embrapa Soja, C.P. 231 86001-970, Londrina Paraná, Brazil; Departamento de Genética, Universidade Federal do Paraná, C.P. 19031 81531-900, Curitiba, PR Brazil; Departamento de Biologia Estrutural, Molecular e Genética, Universidade Estadual de Ponta Grossa, C.P. 6001 84030-900 Ponta Grossa, PR Brazil; Embrapa Recursos Genéticos e Biotecnologia, PqEB s/n°, 70770-901 Brasília, DF Brazil

**Keywords:** Symbiosis, Nitrogen fixation, Two-dimensional proteomics, RT-qPCR, *Bradyrhizobium*

## Abstract

**Background:**

Strain CPAC 7 (=SEMIA 5080) was recently reclassified into the new species *Bradyrhizobium diazoefficiens*; due to its outstanding efficiency in fixing nitrogen, it has been used in commercial inoculants for application to crops of soybean [*Glycine max* (L.) Merr.] in Brazil and other South American countries. Although the efficiency of *B. diazoefficiens* inoculant strains is well recognized, few data on their protein expression are available.

**Results:**

We provided a two-dimensional proteomic reference map of CPAC 7 obtained under free-living conditions, with the successful identification of 115 spots, representing 95 different proteins. The results highlighted the expression of molecular determinants potentially related to symbiosis establishment (e.g. inositol monophosphatase, IMPase), fixation of atmospheric nitrogen (N_2_) (e.g. NifH) and defenses against stresses (e.g. chaperones). By using bioinformatic tools, it was possible to attribute probable functions to ten hypothetical proteins. For another ten proteins classified as “NO related COG” group, we analyzed by RT-qPCR the relative expression of their coding-genes in response to the nodulation-gene inducer genistein. Six of these genes were up-regulated, including blr0227, which may be related to polyhydroxybutyrate (PHB) biosynthesis and competitiveness for nodulation.

**Conclusions:**

The proteomic map contributed to the identification of several proteins of *B. diazoefficiens* under free-living conditions and our approach—combining bioinformatics and gene-expression assays—resulted in new information about unknown genes that might play important roles in the establishment of the symbiosis with soybean.

**Electronic supplementary material:**

The online version of this article (doi:10.1186/1471-2164-15-643) contains supplementary material, which is available to authorized users.

## Background

Biological N_2_ fixation (BNF) is a fundamental component of the global nitrogen (N) cycle, both in natural and agricultural environments. The symbiosis of legumes with soil-borne symbiotic N_2_-fixing bacteria, which are frequently referred to as rhizobia, can often provide more than 60% of the plant’s N requirements [1, 2]. Regarding the concept of agriculture sustainability, BNF contributes to the improvement of food production without cultivation of new lands, to lowering input costs for the farmers and to mitigating environmental degradation. Such benefits occur when BNF replaces chemical N-fertilizers, which are expensive, and, among other harmful environment impacts, foment greenhouse-gas emissions [3, 4].

Cultivation of soybean [*Glycine max* (L.) Merr.] has increased globally, mainly due to its high protein and oil contents, and plant breeding has resulted in increasing yields [5]. Certainly, efficient BNF is a major contributor to the achievement of high yields with low input costs [6]. An important example is the contribution of BNF to soybean cropping in Brazil, associated with application to the seeds at sowing of inoculants containing elite strains of *Bradyrhizobium*, including CPAC 15 (=SEMIA 5079) and CPAC 7 (=SEMIA 5080) [7, 8]. The combination of these strains can fulfill much of the crop’s N needs, resulting in estimated savings of about US $15 billion in N-fertilizers per cropping season [9].

*Bradyrhizobium diazoefficiens* was recently reclassified as a novel species on the bases of morpho-physiological, genotypic and genomic differences from *Bradyrhizobium japonicum*[10]. Strain CPAC 7 (=SEMIA 5080) has outstanding efficiency in fixing N_2_ with soybean and good adaptation to the often-stressful edaphoclimatic conditions of the tropics [11, 12]. These features are responsible for the inclusion of this strain in inoculants applied to soybean in Brazil since 1992 [7, 9].

The type strain of *B. diazoefficiens*, USDA 110^T^ has had its genome elucidated; however, of the 8,317 potential protein-encoding genes, 30% were assigned as hypothetical and 18% showed no similarity to any known gene [13]. Later, the expression of several predicted protein-coding genes in USDA 110^T^ was reported in transcriptomic and proteomic studies [14–19]. Nevertheless, despite the economic importance of *B. diazoefficiens* as a component of soybean inoculants worldwide [9, 10], few data are available on the proteins it synthesizes in the free-living state. It is well known that major attributes of successful elite strains, such as saprophytic competence, adaptation to stressful conditions and nodulation competitiveness must be expressed when free-living.

Our research group has just completed the genome sequencing of strain CPAC 7 [20] and, as occurred with USDA 110 [13], it was not possible to attribute functions to about 50% of the genes. Therefore, the establishment of a proteomic reference map for this strain in the free-living state can both add valuable protein-expression data to the genomic-annotation process [21–23] and help to attribute probable functions to hypothetical proteins [21, 23].

Here we present the first two-dimensional proteomic reference map for free-living *B. diazoefficiens* strain CPAC 7, emphasizing molecular determinants of symbiosis-establishment and of tolerance of environmental stresses. Additionally, we ascribe putative functions to some hypothetical proteins detected at the proteomic level. For other hypothetical proteins without available information, we analyzed the relative expression of their coding-genes in response to the main soybean-nodulation-inducing molecule, the flavonoid genistein.

## Results and discussion

### Two-dimensional gel electrophoresis and protein identification

In studies with two-dimensional gels, it is necessary to optimize the resolution of the protein maps as a function of the nature and characteristics of the samples studied. With this goal, a previous experiment to obtain an overview of the protein distribution of *B. diazoefficiens* strain CPAC 7 was carried out with a broad-range IPG strip (pH 3–10) for the first-dimensional protein separation. After SDS-PAGE, the results showed that most of protein spots remained clustered in the pI range of pH 5–7 (data not shown). To improve the separation of the proteins, we then employed in the first-dimension electrophoresis IPG strips with a narrower pH range (pH 4–7), that confirmed, in triplicated gels, better resolution than the strips with pH 3–10.

**Figure 1:**
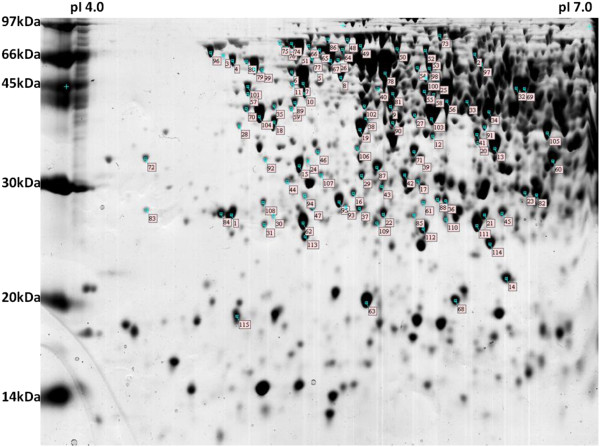
**Two-dimensional electrophoresis protein profile of** ***Bradyrhizobium diazoefficiens*** **CPAC 7 whole cell extract at free-living state.** More information about expressed proteins is available in Tables 1 and 2.

Using computer-assisted gel-image analysis software, well-defined spots were detected and the majority of their molecular weights ranged between 14 kDa and 97 kDa (Figure 1). Among these, 150 spots were selected and analyzed by MALDI-TOF MS or, when necessary, by MS/MS. Mass spectra of peptide fragments were compared with database entries, regarding the statistical requirements, and 115 spots were successfully identified, representing 95 different proteins (Tables 1 and 2). Information on the spectrometry data set is available in Additional file 1: Table S1. The presence of distinct spots for the same protein may be the result of posttranslational modifications.

**Table 1:** **Identified proteins of** ***Bradyrhizobium diazoefficiens*** **CPAC 7 whole cell extract and classification according to COG**

Spot ID	Gene	Product	NCBI ID	Cellular location	*T/**E pI	*T/**E MW	Organism
**Metabolism**							
**Energy production and conversion**							
1	*nuoC*	NADH dehydrogenase subunit C	gi|27380028	Cytoplasmic	4.88/4.94	23201/27000	*B. diazoefficiens* USDA 110
2	*sdhA*	Succinate dehydrogenase flavoprotein subunit	gi|27375625	Periplasmic	5.91/6.34	66903/70000	*B. diazoefficiens* USDA 110
3	*pdhB*	Pyruvate dehydrogenase subunit beta	gi|27379893	Cytoplasmic	4.81/4.90	48906/65000	*B. diazoefficiens* USDA 110
4	*pdhB*	Pyruvate dehydrogenase subunit beta	gi|27379893	Cytoplasmic	4.81/4.95	48906/65000	*B. diazoefficiens* USDA 110
5	*atpD*	ATP synthase F0F1 subunit beta	gi|27375551	Cytoplasmic	5.13/5.42	50987/62000	*B. diazoefficiens* USDA 110
6	*atpD*	ATP synthase F0F1 subunit beta	gi|27375552	Cytoplasmic	5.13/5.28	50987/62000	*B. diazoefficiens* USDA 110
7	*atpD*	ATP synthase F0F1 subunit beta	gi|27375551	Cytoplasmic	5.13/5.35	50987/57000	*B. diazoefficiens* USDA 110
8		Aldehyde dehydrogenase	gi|27379895	Cytoplasmic	6.04/5.57	55297/60000	*B. diazoefficiens* USDA 110
9		Succinate-semialdehyde dehydrogenase	gi|27379109	Cytoplasmic	5.3/5.86	50087/51000	*B. diazoefficiens* USDA 110
10	*eno*	Phosphopyruvate hydratase	gi|27379905	Cytoplasmic	5.08/5.36	45314/53000	*B. diazoefficiens* USDA 110
11	*eno*	Phosphopyruvate hydratase	gi|27379905	Cytoplasmic	5.08/5.28	45314/55000	*B. diazoefficiens* USDA 110
12	*mdh*	Malate dehydrogenase	gi|384214148	Cytoplasmic	5.88/6.09	34259/41000	*B. japonicum* USDA 6
13	*mdh*	Malate dehydrogenase	gi|27375567	Cytoplasmic	5.88/6.09	34275/41000	*B. diazoefficiens* USDA 110
14		Rieske iron-sulfur protein	gi|354959910	Cytoplasmic	5.9/6.52	15192/22000	*B. japonicum* USDA 6
15	*etfL*	Electron transfer flavoprotein large subunit	gi|27376489	Cytoplasmic	5.14/5.33	32186/34000	*B. diazoefficiens* USDA 110
16		Ferredoxin NADP + reductase	gi|27375211	Cytoplasmic	5.54/5.64	31764/29000	*B. diazoefficiens* USDA 110
**Carbohydrate transport and metabolism**							
17		Inositol monophosphatase	gi|27382842	Cytoplasmic	5.61/6.02	28290/31000	*B. diazoefficiens* USDA 110
18		Sugar kinase	gi|27375915	Cytoplasmic	5.06/5.20	35336/38000	*B. diazoefficiens* USDA 110
19		Sugar ABC transporter substrate-binding protein	gi|27378319	Periplasmic	5.63/5.68	38378/38000	*B. diazoefficiens* USDA 110
20		6-Phosphogluconate dehydrogenase	gi|27381870	Cytoplasmic	5.88/6.35	35880/36000	*B. diazoefficiens* USDA 110
**Amino acid transport and metabolism**							
21	*trpF*	N-(5'-phosphoribosyl)anthranilate isomerase	gi|27375855	Cytoplasmic	5.82/6.36	23966/27000	*B. diazoefficiens* USDA 110
22	*leuD*	Isopropylmalate isomerase small subunit	gi|27375606	Cytoplasmic	5.52/5.74	22781/27000	*B. diazoefficiens* USDA 110
23		Amino acid ABC transporter substrate-binding protein	gi|27379557	Periplasmic	6.21/6.60	36860/30000	*B. diazoefficiens* USDA 110
24	*dapF*	Diaminopimelate epimerase	gi|27375588	Cytoplasmic	5.09/5.30	31803/33000	*B. diazoefficiens* USDA 110
26	*glnA*	Glutamine synthetase	gi|27380060	Cytoplasmic	5.44/5.47	52623/66000	*B. diazoefficiens* USDA 110
27		L-asparaginase	gi|27380061	Periplasmic	5.93/5.93	39549/40000	*B. diazoefficiens* USDA 110
28	*serB*	Phosphoserine phosphatase	gi|27381616	Cytoplasmic	4.83/4.93	32322/39000	*B. diazoefficiens* USDA 110
29	*argB*	Acetylglutamate kinase	gi|27383212	Cytoplasmic	5.33/5.60	31294/32000	*B. diazoefficiens* USDA 110
**Coenzyme transport and metabolism**							
31	*thiE*	Thiamine-phosphate pyrophosphorylase	gi|27381769	Cytoplasmic	4.99/5.06	22484/26000	*B. diazoefficiens* USDA 110
32	*ahcY*	S-Adenosyl-L-homocysteine hydrolase	gi|27381055	Cytoplasmic	6.00/6.55	52318/45000	*B. diazoefficiens* USDA 110
33	*metK*	S-Adenosylmethionine synthetase	gi|27381056	Cytoplasmic	5.88/6.25	43613/42000	*B. diazoefficiens* USDA 110
34	*metK*	S-Adenosylmethionine synthetase	gi|27381056	Cytoplasmic	5.88/6.38	43613/41000	*B. diazoefficiens* USDA 110
35	*hemB*	Delta-aminolevulinic acid dehydratase/Porphobilinogen synthase	gi|1170210	Cytoplasmic	4.99/5.11	38843/42000	*B. diazoefficiens* USDA 110
**Nucleotide transport and metabolism**							
38	*hemH*	Phosphoribosylaminoimidazole-succinocarboxamide synthase	gi|27375923	Cytoplasmic	5.35/5.64	33736/40000	*B. diazoefficiens* USDA 110
39	*hemH*	Phosphoribosylaminoimidazole-succinocarboxamide synthase	gi|27375923	Cytoplasmic	5.35/5.90	33736/34000	*B. diazoefficiens* USDA 110
**Lipid transport and metabolism**							
40		3-Oxoacyl-ACP synthase	gi|27378919	Cytoplasmic	5.56/5.71	44874/45000	*B. diazoefficiens* USDA 110
41	*ispH*	4-Hydroxy-3-methylbut-2-enyl diphosphate reductase	gi|27376425	Cytoplasmic	5.75/6.3	35239/37000	*B. diazoefficiens* USDA 110
42	*fabD*	Nitrogenase iron protein ACP S-malonyl transferase	gi|27379193	Cytoplasmic	5.57/5.87	32462/32000	*B. diazoefficiens* USDA 110
43	*fadB*	Enoyl CoA hydratase	gi|27378147	Cytoplasmic	5.44/5.73	27829/30000	*B. diazoefficiens* USDA 110
44	*Ipk*	4-Diphosphocytidyl-2-C-methyl-D-erythritol kinase	gi|27377637	Cytoplasmic	5.03/5.19	31076/31000	*B. diazoefficiens* USDA 110
**Inorganic ion transport and metabolism**							
45	*modA*	ABC transporter molybdenum-binding protein	gi|27383271	Periplasmic	6.62/6.46	27290/27000	*B. diazoefficiens* USDA 110
46	*nifH*	Nitrogenase iron protein	gi|128264	Cytoplasmic	5.03/5.36	31902/35000	*B. diazoefficiens* USDA 110
**Secondary metabolites biosynthesis, transport and catabolism**							
47		Thiol oxidoreductase FrnE	gi|27380891	Cytoplasmic	5.1/5.33	24357/28000	*B. diazoefficiens* USDA 110
**Information storage and processing**							
**Translation, ribosomal structure and biogenesis**							
48	*fusA*	Elongation factor G	gi|27380514	Cytoplasmic	5.32/5.52	76067/84000	*B. diazoefficiens* USDA 110
49	*fusA*	Elongation factor G	gi|27380514	Cytoplasmic	5.32/5.60	76067/81000	*B. diazoefficiens* USDA 110
50	*typA*	GTP-binding tyrosin phosphorylated protein	gi|27375651	Cytoplasmic	5.49/5.82	67131/74000	*B. diazoefficiens* USDA 110
51	*typA*	GTP-binding tyrosin phosphorylated protein	gi|27375651	Cytoplasmic	5.49/5.26	67131/72000	*B. diazoefficiens* USDA 110
52	*aspS*	Aspartyl-tRNA synthetase	gi|27379254	Cytoplasmic	5.71/6.00	66989/73000	*B. diazoefficiens* USDA 110
53		Transporter ATP-binding protein	gi|27381796	Inner-membrane	5.58/6.00	61850/67000	*B. diazoefficiens* USDA 110
54	*gatA*	Aspartyl/glutamyl-tRNA amidotransferase subunit A	gi|13470863	Cytoplasmic	5.66/5.95	55801/59000	*Mesorhizobium loti* MAFF303099
55	*tuf*	Elongation factor Tu	gi|27380513	Cytoplasmic	5.78/5.99	43569/44000	*B. diazoefficiens* USDA 110
56	*tuf*	Elongation factor Tu	gi|27380513	Cytoplasmic	5.79/6.12	43569/43000	*B. diazoefficiens* USDA 110
57	*tuf*	Elongation factor Tu	gi|27380513	Cytoplasmic	5.78/5.00	43569/42000	*B. diazoefficiens* USDA 110
58	*tuf*	Elongation factor Tu	gi|27380513	Cytoplasmic	5.79/6.05	43569/43000	*B. diazoefficiens* USDA 110
59	*hisZ*	ATP phosphoribosyltransferase	gi|27382635	Cytoplasmic	5.1/5.22	41078/41000	*B. diazoefficiens* USDA 110
60		Elongation factor Ts	gi|27379971	Cytoplasmic	6.17/6.77	32175/33000	*B. diazoefficiens* USDA 110
61		Sigma-54 modulation protein	gi|27375835	Cytoplasmic	5.62/5.97	21727/29000	*B. diazoefficiens* USDA 110
62	*rplI*	50S ribosomal protein L9	gi|27379187	Cytoplasmic	5.08/5.22	21886/26000	*B. diazoefficiens* USDA 110
63	*rpsF*	ACP S-malonyltransferase	gi|27379190	Cytoplasmic	5.46/5.64	18616/20000	*B. diazoefficiens* USDA 110
**RNA processing and modification**							
64	*rpsA*	30S ribosomal protein S1	gi|384214454	Cytoplasmic	5.27/5.49	62737/71000	*B. japonicum* USDA 6
65	*rpsA*	30S ribosomal protein S1	gi|27375851	Cytoplasmic	5.27/5.38	64213/70000	*B. diazoefficiens* USDA 110
66	*rpsA*	30S ribosomal protein S1	gi|27375851	Cytoplasmic	5.27/5.31	64213/71000	*B. diazoefficiens* USDA 110
67	*rpsA*	30S ribosomal protein S1	gi|27375851	Cytoplasmic	5.27/5.42	64213/64000	*B. diazoefficiens* USDA 110
**Transcription**							
68	*greA*	Transcription elongation factor GreA	gi|27382489	Cytoplasmic	5.67/6.18	17169/20000	*B. diazoefficiens* USDA 110
69	*rho*	Transcription termination factor Rho	gi|27375746	Cytoplasmic	6.08/6.6	47121/45000	*B. diazoefficiens* USDA 110
70	*rpoA*	DNA-directed RNA polymerase alpha subunit	gi|354957001	Cytoplasmic	4.91/4.97	38082/41000	*B. diazoefficiens* USDA 110
71	*rpoA*	DNA-directed RNA polymerase subunit alpha	gi|27380487	Cytoplasmic	4.9/5.5	38035/35000	*B. diazoefficiens* USDA 110
**Cellular processes and signaling**							
**Posttranslational modification, protein turnover, chaperones**							
73	*clpB*	ATP-dependent protease ATP-binding subunit	gi|27376515	Cytoplasmic	5.7/6.10	96620/90000	*B. diazoefficiens* USDA 110
74	*dnaK*	Heat shock protein	gi|12642164	Cytoplasmic	5.27/5.21	65113/72000	*Bradyrhizobium* sp. WM9
75	*htpG*	Heat shock protein 90	gi|27382900	Cytoplasmic	5.08/5.16	69004/72000	*B. diazoefficiens* USDA 110
76	*htpG*	Heat shock protein 90	gi|27382900	Cytoplasmic	5.08/5.19	69004/710000	*B. diazoefficiens* USDA 110
77	*groEL*	Molecular chaperone GroEL	gi|27377170	Cytoplasmic	5.19/5.32	57749/65000	*B. diazoefficiens* USDA 110
78	*groEL*	Molecular chaperone GroEL	gi|27380737	Cytoplasmic	5.45/5.75	57716/58000	*B. diazoefficiens* USDA 110
79	*tig*	Trigger factor	gi|27380056	Cytoplasmic	4.87/5.06	50061/57000	*B. diazoefficiens* USDA 110
80	*tig*	Trigger factor	gi|27380056	Cytoplasmic	4.87/4.97	50061/64000	*B. diazoefficiens* USDA 110
81	*clpX*	ATP-dependent protease ATP-binding subunit ClpX	gi|27380054	Cytoplasmic	5.575.80	46932/44000	*B. diazoefficiens* USDA 110
82		Anti-oxidant protein	gi|27380419	Cytoplasmic	6.1/6.66	24420/29000	*B. diazoefficiens* USDA 110
84	*grpE*	Heat shock protein GrpE	gi|27375787	Cytoplasmic	4.84/4.84	21642/27000	*B. diazoefficiens* USDA 110
85	*Pcm*	Protein-L-isoaspartate O-methyltransferase	gi|27379583	Cytoplasmic	5.95/5.93	25182/27000	*B. diazoefficiens* USDA 110
**Cell cycle control, cell division, chromosome partitioning**							
86	*ftsZ*	Cell division protein FtsZ	gi|27381707	Periplasmic	5.21/5.40	62990/72000	*B. diazoefficiens* USDA 110
**Signal transduction mechanisms**							
88		Two-component response regulator OmpR	gi|27377311	Cytoplasmic	5.69/6.07	26233/29000	*B. diazoefficiens* USDA 110
**Poorly characterized**							
**General function prediction only**							
89	*ychF*	GTP-dependent nucleic acid-binding protein EngD	gi|27382550	Cytoplasmic	5.13/5.22	39493/42000	*B. diazoefficiens* USDA 110
90	*cobS*	Cobalt insertion protein	gi|383768898	Cytoplasmic	5.62/5.80	37304/39000	*Bradyrhizobium* sp. S23321
91		Dehydrogenase	gi|27378316	Cytoplasmic	5.92/6.32	33290/38000	*B. diazoefficiens* USDA 110
93	*cinA*	Competence-damage associated protein	gi|27380722	Cytoplasmic	5.29/5.51	26451/28000	*B. diazoefficiens* USDA 110
**NO related COG**							
96	*nusA*	Transcription elongation factor NusA	gi|27375896	Cytoplasmic	4.70/4.77	59333/67000	*B. diazoefficiens* USDA 110
97		ATP-dependent phosphoenolpyruvate carboxykinase	gi|398824719	Cytoplasmic	6.01/6.34	59235/62000	*Bradyrhizobium* sp. YR681
98		ABC transporter substrate-binding protein	gi|27380707	Periplasmic	6.93/6.00	59019/58000	*B. diazoefficiens* USDA 110
100	*tldD*	TldD protein	gi|27376279	Cytoplasmic	5.516.00	51101/51000	*B. diazoefficiens* USDA 110
102		ABC transporter substrate-binding protein	gi|27382938	Periplasmic	5.57/5.66	47774/42000	*B. diazoefficiens* USDA 110
104	*glpX*	Fructose 1,6-bisphosphatase II	gi|27379474	Cytoplasmic	5.68/6.06	35603/4000	*B. diazoefficiens* USDA 110
104		Substrate-binding protein	gi|27382959	Periplasmic	6.93/5.35	40839/41000	*B. diazoefficiens* USDA 110
105	*ilvC*	Ketol-acid reductoisomerase	gi|384216635	Cytoplasmic	6.09/6.70	37076/38000	*B. diazoefficiens* USDA 110
106		Dioxygenase	gi|27377910	Cytoplasmic	5.35/5.58	34110/35000	*B. diazoefficiens* USDA 110
111		ATP-dependent Clp protease proteolytic subunit	gi|338974245	Cytoplasmic	5.97/6.32	22404/26000	*B. diazoefficiens* USDA 110

^*^Theoretical and ^**^Experimental.

Matched peptides masses and MS/MS combined results are available at the Additional file 1: Table S1.

**Table 2:** **Functional prediction of hypothetical proteins identified in** ***Bradyrhizobium diazoefficiens*** **CPAC 7 whole protein extract based on protein sequences, conserved domains and motifs, protein-protein interactions and cellular locations**

Spot ID	Hypothetical protein	NCBI ID	Cellular location	*T/**E pI	*T/**E MW	Predicted function
**Metabolism**						
**Amino acid transport and metabolism**						
25	Blr3064	gi|27378175	Cytoplasmic	5.64/6.00	50837/49000	Succinyl-diaminopimelate desuccinylase (COG/NCBI); Peptidase family M20 - dimerisation domain (Pfam); GO: Hydrolase activity (InterPro); Lysine biosynthesis EC:3.5.1.18 (KEGG).
30	Blr5678	gi|27380789	Periplasmic	5.84/5.11	33744/27000	L-aminopeptidase/D-esterase (COG); Peptidase family S58 (Pfan); GO:arginine biosynthetic process; DmpA/ArgJ- Like domains (InterPro).
**Coenzyme transport and metabolism**						
36	Blr3798	gi|27378909	Cytoplasmic	5.66/6.13	27452/29000	Demethylmenaquinone methyltransferase (COG); Methyltransf_6 (Pfam);GO: methyltransferase activity (UniProtKB); Ribonuclease E inhibitor RraA domain (InterPro).
37	Bll4565	gi|27379676	Cytoplasmic	5.3/5.59	24885/28000	Demethylmenaquinone methyltransferase (COG); Methyltransf_6 (Pfam);GO: methyltransferase activity (UniProtKB); Ribonuclease E inhibitor RraA domain (InterPro).
**Information storage and processing**						
**Transcription**						
72	Bll4752	gi|27379863	Cytoplasmic	4.41/4.43	27960/32000	Predicted transcriptional regulator containing the HTH domain (COG); Putative transcriptional regulators (Ypuh-like)(Pfam); Winged helix-turn-helix DNA-binding domain (InterPro).
**Cellular processes and signaling**						
**Posttranslational modification, protein turnover, chaperones**						
83	BJ6T_08050***	gi|354953419	Cytoplasmic	4.46/4.43	20515/28000	Thioredoxin-like proteins and domains(COG); Scaffold protein Nfu/NifU N terminal (Pfam); GO: Iron-sulfur cluster binding (InterPro).
**Signal transduction mechanisms**						
87	Blr2761	gi|27377872	Cytoplasmic	5.465.70	29257/33000	Universal stress protein UspA (COG); Universal stress protein family (Pfam); GO: response to stress (InterPro).
**Poorly characterized**						
**General function prediction only**						
92	Bll5663	gi|27380774	Cytoplasmic	4.92/5.08	33547/34000	MoxR-like ATPases (COG); ATPases Associated with diverse cellular Activities – AAA proteins (Pfam); GO: ATPase activity (InterProt).
**Function unknown**						
94	Blr5067	gi|27380178	Cytoplasmic	5.25/5.29	24247/29000	Uncharacterized ACR (COG); putative metal binding site - region_name = "LabA (NCBI); NYN domain (Pfam).
95	Blr5067	gi|27380178	Cytoplasmic	5.93/5.96	16852/28000	Uncharacterized ACR (COG); putative metal binding site - region_name = "LabA (NCBI); NYN domain (Pfam).
**NO related COG**						
99	Bll0565	gi|27375676	Periplasmic	4.98/5.04	41554/57000	No related data
101	Blr7534	gi|27382645	Periplasmic	5.86/4.99	49518/45000	No related data
107	Bll5131	gi|27380242	Extracellular	7.68/5.80	34214/32000	Protein of unknown function DUF (Pfam/InterPro).
108	Blr2961	gi|27378072	Cytoplasmic	4.96/5.06	25510/29000	GO: catalytic activity/Metabolic process (InterPro); Protein of unknown function (Pfam).
109	Bll5307	gi|27380418	Periplasmic	5.55/5.70	14177/25000	No related data
110	Blr2191	gi|27377302	Cytoplasmic	6.58/6.12	25491/27000	Uncharacterized protein conserved in bacteria (DUF2328) (Pfam). KO: chpT histidine phosphotransferase (KEGG); Two-component system/His Kinase A (phospho-acceptor) domain (IMG).
112	Bll7551	gi|27382662	Cytoplasmic	5.95/5.92	27565/26000	No related data
113	Blr0227	gi|27375338	Periplasmic	5.17/5.29	22619/25000	PHB accumulation regulatory domain (Pfam);GO: Regulation of transcription/Transcription repressor activity (NCBI/InterPro).
114	Blr0227	gi|27375338	Periplasmic	5.17/6.40	22619/24000	PHB accumulation regulatory domain (Pfam);GO: Regulation of transcription/Transcription repressor activity (NCBI/InterPro).
115	Blr7436	gi|27382547	Cytoplasmic	4.82/4.90	15290/18000	No related data

^*^Theoretical and ^**^Experimental.

***Best match with *Bradyrhizobium japonicum* USDA 6.

Matched peptides masses and MS/MS combined results are available at the Additional file 1: Table S1.

### Protein functional classification and cellular location

**Figure 2:**
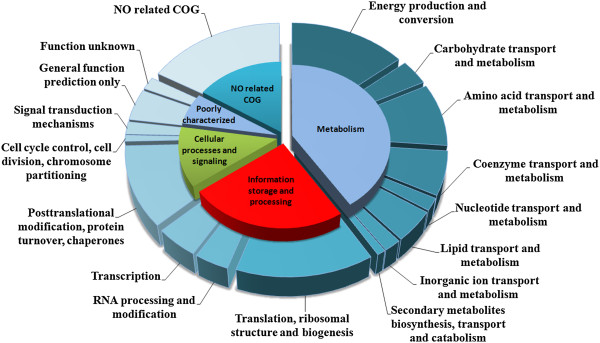
**Functional distribution of identified proteins of** ***Bradyrhizobium diazoefficiens*** **CPAC 7 into COG categories.**

According to the functional classification in COG, proteins were distributed in 16 categories, belonging to four functional groups (Figure 2). In the metabolism-related functional group, there were eight categories, comprising the greatest number of experimentally identified proteins (40%). Next, 21% of the proteins were clustered in three functional categories related to information storage and processing, while the cellular-processes signaling group encompassed 13% of the proteins distributed in three categories. Finally, seven proteins were pooled in two other categories of a poorly characterized group, and 20 proteins did not fit any of the COG categories, being assigned as “not in COG” (Figure 2).

The high percentage of proteins with metabolic functions in CPAC 7 (Figure 2) is consistent with rhizobia’s ability to adapt to varied edaphoclimatic conditions. Several of these proteins are related to energy metabolism, e.g. succinate dehydrogenase flavoprotein subunit and malate dehydrogenase, which participate in the tricarboxylic acid (TCA) cycle, the main pathway for obtaining energy and also important in the synthesis of precursors of the biosynthesis of amino acids, purines, pyrimidines and vitamins [23].

Proteins associated with amino-acid and lipid metabolism may be particularly important for free-living cells, and several of those proteins identified in CPAC 7 have been previously reported in *B. japonicum* CPAC 15 [23]. Beyond their main functions, proteins in these two categories may also play important roles at various stages of the symbiotic interaction, since auxotrophic mutants in both of them are defective in both nodulation and in N_2_-fixation abilities [24].

The second largest functional group—information storage and processing (Figure 2)—encompassed several transcriptional and translational factors (Table 1). These proteins have fundamental roles in controlling metabolic pathways because they regulate and ensure the accuracy of gene expression [25]; furthermore, under stress conditions they can also perform as chaperonins, helping in *de novo* protein folding and preventing damaged proteins from forming aggregates [26, 27].

Most proteins belonging to the cellular-processes-and-signaling functional category were correlated with defense against stressful conditions (Table 1). Mechanisms of response to stresses are usually conserved among bacterial species, and required for rapid adaptation to environmental and metabolic changes. One of these responses comprises the expression of molecular chaperones, such as DnaK, GrpE and, GroEL [28], all of which were detected in our study (Table 1). Also related to the mediation of adaptive responses to adverse conditions [29, 30], two Clp proteases, ClpB and ClpX, were expressed in *B. diazoefficiens* CPAC 7. Finally, there was a cytoplasmic protein member of the two component response regulator OmpR family; proteins of this family are amongst the best characterized bacterial positive regulators, improving the transcriptional capacity of RNA polymerase, with reported effects in osmoregulation in *Escherichia coli*[31].

The cellular locations of all 115 identified proteins, predicted by PSORT-B and PSLpred, are listed in Tables 1 and 2. Although the majority of the proteins extracted in our study are located in cell cytoplasm and periplasm, two inner-membrane and one extracellular protein were obtained. Similar results have been reported in previous rhizobial proteomic studies by our group [23, 32].

### Symbiosis establishment and N_2_-fixation-related proteins

In the establishment of the legume-rhizobia symbiosis, an exchange of molecular signals starts with the host plant’s release of molecules, mainly flavonoids, that induce expression of rhizobial nodulation (*nod*) genes. The products of *nod*-genes, the Nod factors (lipo-chitin oligosaccharides, LCOs), play critical roles in root nodulation [33]. Host specificity is also determined by the Nod factors, by means of the incorporation of α,β-unsaturated acyl chains in the backbone structure [34]. In our proteomic reference map, we identified one acyl carrier protein, 3-oxoacyl-ACP synthase, and one ACP S-malonyl transferase (Table 2), both required for the synthesis of essential fatty acyl chains.

Also important to the production of an effective N_2_-fixing symbiosis, the exopolysaccharides (EPSs) play an essential role in the symbiotic interaction with compatible host plants [35, 36]. We found one inositol monophosphatase (IMPase) (Table 1); this protein has been related with the regulation of EPS production, which, when mutated in *Rhizobium leguminosarum* bv. trifolii, resulted in defective-EPS production and a non-N_2_-fixing phenotype [37–39].

Several microbial factors, classified either as general or host-specific elicitors, are related to the induction of immune responses in plants [40, 41]. General elicitors include flagellins, cold-shock proteins (CSPs), LCOs and LPSs. We identified the elongation factor Tu (EFTu), which also acts as elicitor and, in general, is conserved across multiple groups of bacteria, allowing plants to perceive and respond to an epitope common to many bacteria [42, 43].

In response to general bacterial elicitors, plants have basal defense mechanisms that include increases in extracellular pH, ethylene production, and synthesis of reactive oxygen species (ROSs) [40]. Upon an initial “unfriendly” reception from the host plant, rhizobia must avoid host defenses, and elicit a successful environment to establish an effective N_2_-fixing symbiosis [44–46]. Among the several features presented by the bacteria to overcome plant defenses, GTP-binding protein TypA, which confers resistance to certain antimicrobial peptides and survival under stress conditions, has been recognized as the main contributor to a successful interaction between *Sinorhizobium* (=*Ensifer*) *meliloti* and some *Medicago truncatula* lines [44]. A probable symbiotic function of this protein was also observed in *B. japonicum* CPAC 15 in response to the host flavonoid genistein [47], and now its constitutive expression has been detected in *B. diazoefficiens* CPAC 7.

The reduction of N_2_ to ammonia by the nitrogenase complex can take place either by the rhizobial bacteroids inside the nodules, or in free-living rhizobia, including some *Bradyrhizobium* strains. In both cases, a finely balanced regulation of oxygen availability is required, since rhizobia are aerobic and need oxygen, whereas the element can denature nitrogenase. Inside the nodule, the ideal oxygen environment is reached by the participation of multiple factors, including the synthesis of heme compounds by the rhizobia [48, 49]. Proteins HemB and HemH, identified in CPAC 7 (Table 1), catalyze two important steps in heme synthesis and are essential for the *Bradyrhizobium*/soybean symbiosis, since mutants defective in these genes generate a microaerobic condition with poorly developed nodules that are inefficient in fixing N_2_[48, 50].

A protein related to amino acid metabolism and also key in N_2_ fixation is the glutamine synthetase I (GS I), which was identified in our proteomic map (Table 1). The role of GS I in the regulation of nitrogenase has been highlighted by studies with *Rhizobium* sp. mutants, resulting in defective ability to derepress the enzyme, both *in vitro*[51] and in symbiotic conditions [52].

In our study, we also detected the constitutive expression of a sigma-54 modulation protein, which fitted in the translation, ribosomal structure and biogenesis functional category (Table 1). Similarly to NifA, this protein participates in controlling the expression of sigma-54 (RpoN*,* NtrA), which, in turn, helps initiate the transcription of genes encoding proteins for diverse cell functions [53, 54]. Among several roles, RpoN is directly involved in the N_2_-fixation process, being required for control of major N_2_-fixation genes, as *nifHDK*, the products of which constitute the nitrogenase complex and accessory proteins [55]. RpoN is also related to free-living metabolic pathways, as demonstrated when rhizobia *rpoN-*mutants showed, beyond defects in symbiotic N_2_-fixation [56], alterations in free-living nitrate assimilation [57]. We may suppose that the constitutive sigma-54 modulation protein may be important in mediating adaptations to changing environmental conditions, both in free-living and in symbiotic conditions.

The expression of the key protein NifH (nitrogenase iron protein, or component II) in our study may be related to the presence of sigma-54 modulation protein, which was shown to be a regulator of the sigma-54 expression. To support this, results show that the knockout of sigma-54 transcriptional factor in *B. japonicum* leads to strong pleiotropic effects, including the absence of NifH [57] and the abolishment of symbiotic N_2_-fixation ability [55]. The detection of NifH in our proteomic study might correlate with reports of expression of nitrogenase in *Bradyrhizobium* strains under free-living conditions; however, measurements of nitrogenase activity under these conditions are difficult and require a fine adjustment of the oxygen concentration [58–61].

### Stress-tolerance proteins

In tropical regions, crops and soil microorganisms are frequently exposed to stressful conditions, in particular high temperatures, salinity and soil acidity [6, 62, 63]. Therefore, in addition to high efficiency of N_2_ fixation, commercial rhizobial strains must be tolerant of such adverse factors. Indeed, several soybean bradyrhizobia have been extensively studied to characterize their tolerances of salt [64], desiccation [65], antibiotics [66], acidity [67], among other stresses. Now, in *B. diazoeficiens* CPAC 7 we produced evidence of several molecular determinants related to the ability to overcome adverse conditions, including chaperonins and other proteins, such as Clp proteases and transcription-elongation factors, with roles in cell defense.

ATP-dependent Clp proteases participate in diverse cell processes, including rapid adaptive responses of bacteria to environmental changes [29] and to stressful conditions [30], and ClpB and ClpX were detected in CPAC 7. These two proteins have the properties associated with molecular chaperones, such as preventing the aggregation of denatured proteins and, in some cases, refolding them [68]. These properties are particularly important under stress conditions that exacerbate the occurrence of protein denaturation, and ClpB and ClpX help to ensure a fast return to the pre-stressed state, maintaining cell homeostasis [69, 70].

*Bradyrhizobium* is acid-tolerant, it grows at pH 4.5, over 30% of the strains are capable of growing at pH 4.0 and a few are tolerant of pH 3.5 [71]. The proteome of *B. diazoefficiens* USDA 110, when studied at pH 4.7 [72], revealed differential expression of several proteins, eight of which—spots 5, 8, 15, 49, 70, 81, 82, and 91—were constitutively expressed in the proteome of CPAC 7 (Table 1).

Another limiting factor for rhizobia, and also for the symbiosis, is high soil temperature, which can often exceed 40°C in the tropics and limit the success of inoculants [6, 8, 62]. Rhizobia, similar to most organisms [73], make use of molecular chaperones to tolerate high temperatures, including heat-shock proteins (HSPs) DnaK, GrpE, GroEL and HtpG, which were identified in our proteomic reference map (Table 1). DnaK and GrpE comprise a versatile chaperone system [74] that, together with GroEL and HtpG, play a critical role in thermotolerance, routinely rescuing the majority of the proteins denatured [69, 75].

Still associated with thermotolerance, it is worth mentioning the identification of three elongation factors (Ef-Tu, Ef-Ts and Ef-G) and two ribosomal proteins (30S and 50S) expressed in CPAC 7. Besides their main function of ensuring gene expression, elongation factors can also act as chaperones [26, 27]. This secondary role has been demonstrated recently at the proteomic level in *Rhizobium tropici* strain PRF 81 (now reclassified as *Rhizobium freirei*) under high-temperature stress [75]. In *B. japonicum*, 30S and 50S ribosomal proteins may be involved in heat-stress defense, once they were up-regulated at 43°C [76]. Considering this finding, those authors hypothesized that ribosomes may act as sensors of heat shock in *B. japonicum.* A similar mechanism has been suggested in *E. coli*, in which ribosomes seem to be the primary sensor of conditions that evoke heat-shock responses [77].

Oxidative stress is frequently caused by cell exposure to reactive oxygen species (ROSs), such as superoxide anion (O_2_^−^) and hydrogen peroxide (H_2_O_2_). ROSs are byproducts of normal metabolic processes and, if not properly detoxified, they become toxic. Oxidative stress also occurs by cell exposure to external ROSs, which in bacteria such as rhizobia may take place during interactions with other microorganisms or eukaryotic hosts. Therefore, tolerating and overcoming oxidative stress is critical to bacteria viability as well as for the establishment of a successful symbiotic infection [32, 78].

Of the proteins identified in CPAC 7, at least five have already been reported as showing antioxidant activity (spots 16, 50, 68, 82 and 83, Table 1). Among them, ferredoxin-NADP^+^ reductase (FRN) has been reported to overcome the harmful effects of ROSs on DNA replication [79]. Those authors emphasized the importance of FRN to cell protection against oxidative damage by comparing its role with those of superoxide dismutases, a group of proteins well known for mitigating damage caused by ROSs.

Salinity leads to loss of intracellular water, resulting in osmotic disturbances that can influence a range of metabolic activities [80]. Indeed, several negative effects in rhizobia-plant symbioses have been attributed to salinity; e.g. in growth and survival of rhizobia in soil, in root colonization and in nodule development [81].

Mutational studies with *S. meliloti* allowed the identification of multiple genes involved in salt tolerance, including trigger factor (*tig*) [82]. When this gene was absent, *Sinorhizobium* showed reduced ability to grow in LB with high salt concentrations, as well inability to compete against the wild-type for nodule occupancy [82]. Given these results, we suggest that *tig* may also contribute to competitiveness and to saprophytic competence under environmentally stressful conditions, as reported for CPAC 7 [7, 8, 10, 11, 22].

Classified in the information storage and processing COG category, the transcription elongation factor GreA has been recognized as a general stress protein (Gsp) induced in response to various environmental conditions. Additionally to the transcription elongation activity, its role in acid-, salt-, and cold-stress responses in *Streptococcus mutans*[83], *S. meliloti*[84] and *R. tropici*[85] has been reported. Constitutive expression of GreA was previously reported in a *B. japonicum* CPAC 15 proteomic assay [23]. Mutation of *greA* in *R. tropici* impaired the establishment of an effective symbiosis as a result of the altered ability to adapt to hyperosmosis and salt stress [85], highlighting the importance of this protein in overcoming adverse conditions during symbiosis establishment.

Altogether, these bacterial defense mechanisms are crucial to survival in the soil and to symbiosis establishment in the tropics, where rhizobia are commonly exposed to high soil temperatures, acid pH and saline conditions [3, 8].

### Hypothetical proteins: function prediction with bioinformatics tools

In several genome projects, portions of the annotated sequences have been classified in “hypothetical”, “conserved hypothetical” or “of unknown function” categories [86, 87]. These denominations are used when the existence of a gene is supported only by prediction of gene-finding software, and they do not show significant homology to any characterized gene [23]. With *B. diazoefficiens* USDA 110, these proteins were abundant, and, of the 8,317 protein-coding genes predicted, 30% showed similarity to hypothetical genes, whereas 18% showed no similarity to any registered gene [13]; in the genome of CPAC 7, both categories comprised 50% of all predicted genes [20]. A still modest improvement in annotation of hypothetical proteins has been achieved with proteomic studies [23, 87]. For example, the reference map of *B. japonicum* CPAC 15 contributed to the assignment of 26 hypothetical proteins by using bioinformatics tools [23].

In our study, 20 proteins were classified as hypothetical/conserved hypothetical or unknown, and by using bioinformatics tools, we were able to attribute probable functions to half of them (Table 2). Two proteins (Blr3064 and Blr5678) were assigned to the amino acid transport and metabolism COG category. The first one shows hydrolase activity and, according to the KEGG database, participates in lysine biosynthesis. Blr5678 is probably related to the arginine biosynthetic process, since it has an ArgJ-like domain.

Classified according to the COGnitor in the coenzyme transport and metabolism functional category, proteins Blr3798 and Bll4565, represented by spots 36 and 37 (Figure 1, Table 2) presented similar information in the databanks searched in our study. Both were annotated as demethylmenaquinone methyltransferase by COG and exhibited a ribonuclease E inhibitor RraA domain. The inhibitory activity of this domain was recently described in *E. coli* as having a regulatory function in gene expression, since the interactions of RraA with RNase E affect the composition of the RNA-degradosome, modulating its activity [88, 89].

The cytoplasmic protein Bll4752, which belongs to the transcriptional COG group is predicted as a transcriptional regulator protein that contains a Ypuh-like helix-turn-helix domain (Table 2). This protein probably plays a role in chromosomal partitioning during cell division.

BJ6T_08050, classified in the posttranslational modification, protein turnover, chaperones functional category, shows 97% of similarity with Bll0800 of *B. diazoefficiens* USDA 110. After comparison of databases, this hypothetical protein was assigned as a thioredoxin-like protein involved in iron transport (Table 2); however, it may be related to reactivation of proteins damaged by oxidative stress [90]. Also potentially related to cell defense, Blr2761 is closely similar to universal stress protein UspA, expression of which is known to be enhanced when cells are exposed to adverse conditions [91].

Two other proteins constitutively expressed in strain CPAC 7—Bll5663 and Blr5067—remained annotated as hypothetical in the NCBInr database (Table 2). Bll5663 is a member of the MoxR family of AAA + ATPases, widespread among bacteria. Associated with diverse cellular activities, MoxR ATPases display a chaperone-like function and have been found to be important modulators of multiple-stress-response pathways in various organisms, including *R. leguminosarum*[92, 93]. The other protein, Blr5067, was also expressed in bacteroids of *B. diazoefficiens* USDA 110, and is still poorly characterized [14]; it could be a LabA-like protein with a putative metal-binding site. This family of proteins has been studied in cyanobacteria and reported to affect both gene expression and cellular metabolic state [94, 95]. Nevertheless, the biological role of Blr5067 in *Bradyrhizobium* remains to be determined.

### Genistein effect on the expression of hypothetical protein-coding genes

Ten out of the 20 hypothetical proteins identified in our proteomic study did not fit into any of the functional categories of COG, being assigned as “NO related COG” (Table 2). The lack of homology of these sequences with those of known proteins, combined with their detection at the proteomic level, suggest that they could be interesting subjects of study, possibly providing new information and deeper understanding of the organism. We analyzed the relative expression patterns of nine hypothetical protein-coding genes (two spots—113 and 114—showed similarity with the same protein Blr0227) in response to genistein. The localization of the genes encoding these proteins in the genome of *B. diazoefficiens* CPAC 7 is shown in Additional file 1: Table S1. Identified as one of the main components present in soybean root extract [96], genistein induces the expression of *nod* genes in *Bradyrhizobium*[16, 97]. In addition, it has been shown that flavonoids, such as genistein, can induce the expression of other genes besides *nod* genes [15, 47].

**Figure 3:**
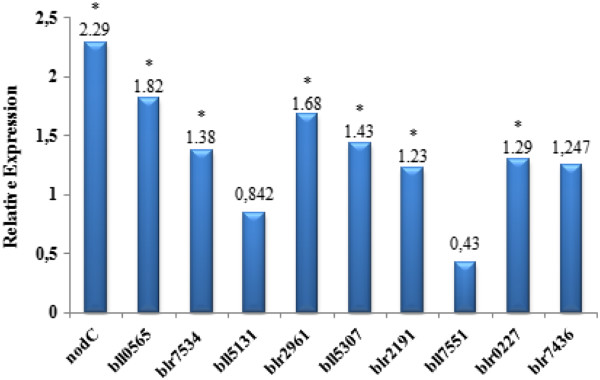
**Expression levels of hypothetical proteins coding genes of** ***Bradyrhizobium diazoefficiens*** **strain CPAC 7 after growth to exponential phase in the presence of genistein.** Relative expression was determined by REST2009 software.

The product of *nodC* directs the synthesis of the backbone of lipochitin oligosaccharides (LCOs), also called Nod factors, which are essential for the nodulation process [97–100]. In our study, the *nodC* gene of CPAC 7 was used as a positive control in the RT-qPCR analysis, and up-regulation was confirmed; in addition, six of the nine hypothetical protein-encoding genes were significantly up-regulated (Figure 3). Of these, bll0565 and blr2961 protein-coding genes showed the highest genistein-induction effect, and up-regulation was observed also for blr7534, bll5307, blr2191 and blr0227.

Blr2191 is a ChpT histidine phosphotransferase, well characterized in *Caulobacter crescentus*, a model organism in cell-cycle studies [101]; in this bacterium, the protein controls, via phosphorylation, the activity of the master cell-cycle regulator CtrA [102, 103]. The role of histidine phosphotransferases in the cell cycle of *B. diazoefficiens* is still to be elucidated; however, with the results from our study—showing its induction by genistein—we may suppose that it affects the growth rate of *B. diazoefficiens* in a genistein-enriched environment, such as the soybean rhizosphere [104].

Protein Blr0227 has also been identified in *B. japonicum* under neutral [23] and acidic conditions [72]. A potential implication of this protein in rhizobial competitiveness might exist, since it is up-regulated in response to the flavonoid daidzein in *B. japonicum* strain 4534, a strain highly competitive for nodulation, but not in the poorly competitive *B. japonicum* strain 4222 [105]; it is noteworthy that CPAC 7 is also competitive. Blr0227 has a polyhydroxybutyrate (PHB)-accumulation regulatory domain and it is known that in *B. japonicum* large amounts of carbon are directed to the synthesis of storage compounds, especially PHB [106]. Interestingly, the PHB biosynthesis also seems to be associated with rhizosphere competitiveness, since *B. japonicum* mutants defective in PHB synthesis show reduced competitiveness [107].

Three out of the nine proteins of CPAC 7 analyzed were not significantly induced by genistein, Bll5131, Bll7551 and Blr7436 (Figure 3, Additional file 2: Figure S1), confirming results reported with *B. japonicum* strain CPAC 15 [47]. They may play other roles, e.g. Bll7551 was one of the up-regulated proteins in bacteroids of *B. japonicum* USDA 110 [108], suggesting a role in latter steps in the development of the symbiosis.

## Conclusions

CPAC 7 is an agronomically important strain used in commercial inoculants for application to soybean crops in Brazil and in other South American countries; it presents high N_2_-fixation efficiency, and adaptability to tropical conditions [7, 8, 11, 109]. Here we provide the first proteomic map for this bacterium, revealing molecular determinants of distinct steps in the establishment and functioning of the symbiotic biological N_2_-fixation process. We also report the constitutive expression of proteins such as DnaK, ferredoxin-NADP^+^ reductase (FRN) and trigger factor (*tig*) related to cell protection against heat-, oxidative- and salt stress conditions, that should contribute to bacterial survival and symbiotic functioning under adverse environmental conditions common in the tropics. In general, no function can be attributed to more than one third of the putative genes in bacterial genomes. With the approach taken in our study—including proteomics, use of bioinformatics tools and transcriptomic assays—it was possible to obtain information about several hypothetical genes/proteins of *B. diazoefficiens*, revealing interesting information, with an emphasis on genistein-induced genes, that deserve further study to confirm their roles in the soybean root-nodule symbiosis.

## Methods

### Strain and culture conditions

*Bradyrhizobium diazoefficiens* strain CPAC 7 (=SEMIA 5080, =CNPSo 6), a natural variant of CB 1809 (=USDA 136, a subculture of USDA 122) [10, 110] is used in commercial inoculants in Brazil since 1992 [7]. Information about morpho-physiologic, genetic and symbiotic properties is available elsewhere [10, 11, 111–113]. CPAC 7 is deposited at the *“*Diazotrophic and Plant Growth Promoting Bacteria Culture Collection*”* of Embrapa Soja (WFCC Collection # 1213, WDCC Collection # 1054).

The strain was pre-cultured in 10-mL aliquots of arabinose-gluconate (AG) medium [114], at 80 rpm and 28°C, in the dark. For the proteomic experiment, pre-cultures were transferred to Erlenmeyer flasks containing 200 mL of the same medium and were grown under the same conditions as for the pre-cultures until the exponential phase (O.D. of 0.6 at 600 nm). Low agitation (80 rpm) was employed to minimize the production of extracellular polysaccharides, which can interfere with the 2-D gel electrophoresis.

For the reverse transcription quantitative PCR (RT-qPCR), pre-cultures were transferred to Erlenmeyer flasks containing 100 mL of AG medium. Bacteria were grown to the exponential phase under two treatment conditions: induced or not with genistein (5 μM, final concentration) dissolved in methanol, added as 50 μL per 100 mL of culture [15]; to the non-induced cultures, the same amount of methanol was added. In both proteomics and qPCR experiments, bacteria were grown in triplicates for each treatment.

### Whole-cell protein extraction

Cultures were centrifuged at 5,000 *g*, at 4°C and cells were carefully washed with a solution containing 3 mM KCl; 1.5 mM KH_2_PO_4_; 68 mM NaCl; and 9 mM NaH_2_PO_4_. Washed cells were resuspended in 600 μL of a buffer containing 10 mM Tris–HCl pH 8.0; 1.5 mM MgCl_2_; 10 mM KCl; 0.5 mM DTT; and 0.5 mM PMSF. Aliquots of 150 μL were stored in ultrafreezer (−80°C) until the analyses.

For total protein extraction, aliquots were resuspended in lysis buffer (9.5 M urea; 2% CHAPS; 0.8% v/v Pharmalyte 4–7; and 1% DTT), and submitted to forty cycles of freezing in liquid N_2_ and thawing at 37°C, as described before [115]. The lysates were separated from particulate material by centrifugation at 14,000 *g* for 90 min, at 4°C. Protein extract was washed with phenol and the concentration was determined by NanoDrop 1000 Spectrophotometer V3.7 (Thermo Scientific).

### Two-dimensional gel electrophoresis and visualization

For isoelectric focusing (IEF), 300 μg of protein extract were dissolved with DeStreak buffer (GE Healthcare) and 2% v/v IPGphor to a final volume of 250 μL. IPG-strips (pH 4–7, 13 cm, GE Healthcare) were rehydrated overnight with the protein solution and covered with Cover Fluid (GE Healthcare). Loaded strips were submitted to isoelectric focalization in an Ettan IPGphor IEF system (GE Healthcare) for 1 h at 200 V, 1 h at 500 V, a gradient step to 1,000 V for 1 h, a gradient step to 8,000 V for 2 h 30 min, and fixed at 8,000 V for 1 h 30 min. The final Vh was fixed at 24,800. Prior to second dimension,, strips were equilibrated first for 20 min in 5 mL of an equilibration buffer (50 mM Tris–HCl pH 8.8; 6 M urea; 30% v/v glycerol; 2% w/v SDS; and 0.2% v/v of a 1% solution of bromophenol blue) supplemented with 50 mg DTT and then in TE buffer with 175 mg of iodoacetamine, also for 20 min.

The second dimension electrophoresis was performed in a 12% polyacrylamide gel in a Ruby SE 600 vertical electrophoresis system (GE Healthcare). The run was carried out for 30 min at 15 mA/gel and 240 min at 30 mA/gel, using the Low Molecular Weight Calibration Kit for SDS Electrophoresis (Amersham Biosciences) as standard. Similarly to the protein extraction step, both dimensions of gel electrophoresis were run in triplicate. Gels were fixed overnight with an ethanol-acetic acid solution before being stained with Coomassie Blue PhastGel™ R-350 (GE Healthcare) and were then scanned (ImageScanner LabScan v5.0).

### Gel image analysis and sample preparation to mass spectrometry

Protein spots were automatically detected in the high-resolution digitized gel images and analyzed by Image Master 2D Platinum v 5.0 software (GE Healthcare). Well defined spots were manually selected, excised and processed as previously described [116]. Digestion was achieved with trypsin (Gold Mass Spectrometry Grade, Promega, Madison, WI) at 37°C, overnight.

Peptides from digested proteins were mixed with saturated solution of α-cyano- 4-hydroxy-cinnamic acid (HCCA) in 50% acetonitrile, 0.1% trifluoroacetic acid (TFA). The mixture was spotted onto a MALDI (Matrix Assisted Laser Desorption Ionization) target plate and allowed to crystallize at room temperature. The same procedure was used for the standard peptide calibration mixture I (Bruker Daltonics). For mass spectra acquisition, a MALDI-TOF-TOF (MALDI-time-of-flight in tandem) UltraFlex III mass spectrometer (Bruker Daltonics) was operated in the reflector for MALDI-TOF MS peptide mass fingerprint (PMF) and in the “LIFT™” mode for MALDI-TOF-TOF MS/MS fragmentation experiments, on fully manual mode using FlexControl software v. 2.2. To process the data obtained, Flex Analysis v.3.0 software (Bruker Daltonics, Billerica, MA) was employed.

### Protein identification

PMFs and MS/MS ion spectra generated were searched against the public database NCBInr (National Center for Biotechnology Information non-redundant), using Mascot software search engine v. 2.3 (http://www.matrixscience.com/). For protein searches, performed in the Proteobacteria taxonomic group, monoisotopic masses were used, considering a peptide tolerance of 150 ppm and allowance of one missed cleavage. When MS/MS was carried out, a tolerance of 0.3 Da was acceptable. Carbamidomethylation of cysteine and oxidation of methionine were considered fixed and variable modifications, respectively.

Identifications were validated only when the Mowse (molecular weight search) score was significant. Searches on the Decoy database (Mascot) were done and both decoy score and false discovery rates were considered for the identification. The spectrometry datasets are available at the Additional file 1: Table S1.

### *In silico*protein characterization

A set of bioinformatics tools was used to improve the characterization of the proteins. The proteins were fitted into COG (Clusters of Orthologous Groups) categories according to their functional inference, using the COGnitor program (http://www.ncbi.nih.gov/COG) [117]. Software packages PSORT-B [118] and PSLpred [119] were used for the prediction of subcellular localization.

To search for putative roles of the hypothetical proteins, a package of bioinformatics tools was applied [87]. SignalP [120] was employed for the prediction of signal peptides. To determine the protein family and domains we used Pfam [121] and InterPro [122]. MicrobesOnline (http://www.microbesonline.org) [123], a suite of web-based comparative tools, and the Integrated Microbial Genomes system (http://img.jgi.doe.gov) [124] were also searched. Finally, the prediction of physical and functional protein interactions was carried out with STRING 9.1 (http://string-db.org/) [125].

### RNA extraction and primers design

Cells from 35 mL of the control and the genistein induced bacterial cultures (item 2.1) were centrifuged (8,000 *g* for 10 min at 4°C) and the pellet was resuspended in 280 μg of lysis buffer, consisted of 250 μL of TE (10 mM Tris, adjusted to pH 8.0 with HCl; 1 mM EDTA), 10 μL of lysozyme (5 mg/mL) and 20 μL of 10% SDS solution (w/v). After resuspended, the mixture was incubated at 37°C for 5 min to achieve an efficient cell disruption. Lysates were then centrifuged (8,000 *g* for 10 min at 4°C) and the supernatants were transferred to new 2-mL tubes and homogenized with 1 mL of TRIzol^®^ reagent (Life Technologies). This new mixture was centrifuged and the superior phase was transferred to another tube. After a wash step with chloroform, RNA was precipitated by adding 500 μL of cold isopropanol, purified with RNeasy Mini Kit (Quiagen) and quantified by NanoDrop ND-1000 (NanoDropTechnologies, Inc.). The RNA was assessed in a 1% (w/v) agarose gel.

Primers were designed using PrimerExpress 3.0 (Applied Biosystems/Life Technologies, Grand Island, NY, USA) targeting an amplicon size of 50–200 bp. The primer sequences were searched against the *B. diazoefficiens* strain USDA 110 genome (http://www.ncbi.nlm.nih.gov/genome/18384) to verify their specificity. Primer sequences and amplification efficiency rates are shown in Additional file 3: Table S2.

Extracted RNAs were submitted to DNAse treatment (Invitrogen/Life Technologies, Grand Island, NY, USA), and high quality total RNA was used to synthesize cDNA strands (Superscript II First Strand Synthesis, Invitrogen/Life Technologies, Grand Island, NY, USA).

### Relative gene expression analysis by RT-qPCR

After carrying out the amplification to determine the primers efficiency rate, nine hypothetical genes were amplified by RT-qPCR using a 7500 RT-qPCR Thermocycler (Applied Biosystems/Life Technologies, Grand Island, NY, USA) with the following manufacturer's instructions: 50°C for 2 min, 95°C for 10 min, 45 cycles at 95°C for 2 min, 60°C for 30 s and 72°C for 30 s, in 45 cycles. The 16S rRNA gene was used as endogenous control (Additional file 3: Table S2).

Rest2009 software package [126] was used to evaluate the data by providing a robust statistical analysis (Additional file 2: Figure S1). The normalization of cycle threshold (Ct) of RT-qPCR amplifications was performed based on the selected endogenous gene (16S rRNA). The genistein responsive gene *nodC*[127] was used as positive control.

## Electronic supplementary material

Additional file 1: Table S1: Complementary information about protein identifications. All searches were performed with Mascot software v. 2.3 (http://www.matrixscience.com/) against the public database NCBInr (National Center for Biotechnology Information non-redundant). *Identified by MS; **Identified by MS/MS. (PNG 92 KB)

Additional file 2: Figure S1: Localization in the genome of *B. diazoefficiens* CPAC 15 of the genes coding hypothetical proteins used in our study. (DOC 240 KB)

Additional file 3: Table S2: Statistical results of RT-qPCR data provided by Rest2009 software package. (DOCX 19 KB)
