# Supplementary figures and images for: Proteomic analysis of free-living Bradyrhizobium diazoefficiens: highlighting potential determinants of a successful symbiosis
Source: BMC Genomics. 2014 Aug 3;15:643. doi: 10.1186/1471-2164-15-643 (PMC4287336; doi:10.1186/1471-2164-15-643)

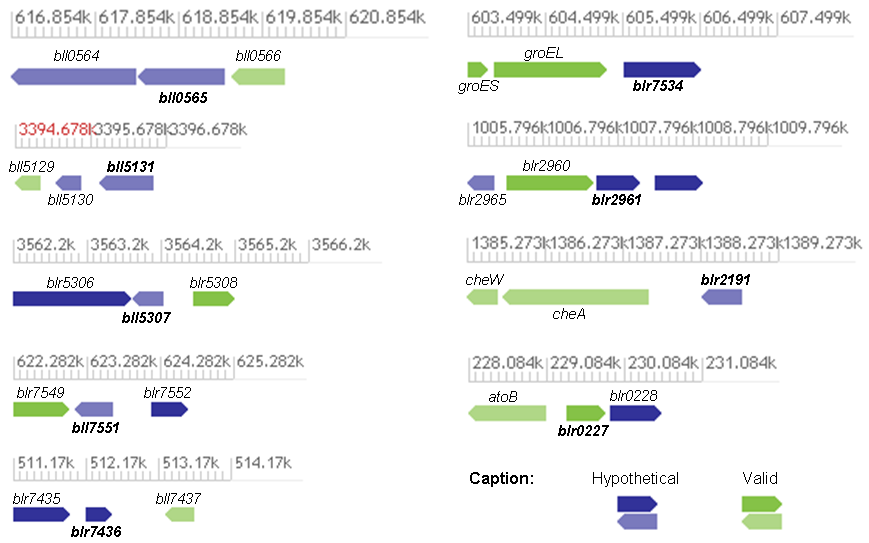

Supplement: Supplementary file 1 — Additional file 1: Table S1: Complementary information about protein identifications. All searches were performed with Mascot software v. 2.3 (http://www.matrixscience.com/) against the public database NCBInr (National Center for Biotechnology Information non-redundant). *Identified by MS; **Identified by MS/MS. (PNG 92 KB) [file 12864_2014_6775_MOESM1_ESM.png]
