# Supplementary material for: Proteomic analysis of free-living Bradyrhizobium diazoefficiens: highlighting potential determinants of a successful symbiosis
Source: BMC Genomics. 2014 Aug 3;15:643. doi: 10.1186/1471-2164-15-643 (PMC4287336; doi:10.1186/1471-2164-15-643)
Supplement: Supplementary file 2 — Additional file 2: Figure S1: Localization in the genome of B. diazoefficiens CPAC 15 of the genes coding hypothetical proteins used in our study. (DOC 240 KB) [file 12864_2014_6775_MOESM2_ESM.doc]

**Additional file 2: Table S1:** Complementary information about protein identifications. All searches were performed with Mascot software v. 2.3 (http://www.matrixscience.com/) against the public database NCBInr (National Center for Biotechnology Information non-redundant). *Identified by MS; **Identified by MS/MS.

Spot ID: 1**

**NADH dehydrogenase subunit C [*Bradyrhizobium japonicum* USDA 110] Mass:** 23201    **Score:** 91     **Matches:** 1(1)  **Sequences:** 1(1)  **Protein sequence coverage:** 5%

**Monoisotopic mass of neutral peptide Mr(calc):** 1266.6245

**Fixed modifications:** Carbamidomethyl (C)

**Ions Matches:** 49/166 fragment ions using 47 most intense peaks.

**Start – End Observed Mr(expt) Mr(calc) ppm M Peptide**

**148 – 158 1267.5437 1266.5364 1266.6245 -69.5 0**

**K.DFPTTGFVEVR.Y**

Spot ID: 2*

**Succinate dehydrogenase flavoprotein subunit [*Bradyrhizobium japonicum* USDA 110]**

**Mass:** 66903 **Score:** 264 **Mass values matched:** 27 **Protein sequence coverage:** 51%

**Start – End Observed Mr(expt) Mr(calc) ppm M Peptide**

**19 – 40 2259.2009 2258.1937 2258.1332 26.8 0 K.AYPIEDHTYDVVVVGAGGAGLR.A**

**41 – 51 1118.6288 1117.6215 1117.5550 59.5 0 R.AVVGCSEAGLR.T**

**94 – 109 1854.8887 1853.8814 1853.8254 30.2 0 K.GSDWLGDQDAIEYMVR.N**

**94 – 109 1870.9198 1869.9125 1869.8203 49.3 0 K.GSDWLGDQDAIEYMVR.N + Oxidation (M)**

**110 – 127 2087.0430 2086.0357 2085.9908 21.5 0 R.NAPDAVYELEHWGVPFSR.T**

**133 – 147 1729.9128 1728.9056 1728.8658 23.0 0 K.IYQRPFGGMTLDFGK.G**

**160 – 174 1702.8691 1701.8619 1701.8079 31.7 0 R.TGHAMLHTMYGQSLR.H**

**160 – 174 1718.8802 1717.8730 1717.8028 40.8 0 R.TGHAMLHTMYGQSLR.H + Oxidation (M)**

**198 – 211 1507.9089 1506.9017 1506.8518 33.1 1 R.GVIALKLDDGTLHR.F**

**212 – 226 1609.9291 1608.9218 1608.8736 29.9 1 R.FRAQTVILATGGYGR.A**

**214 – 226 1306.7726 1305.7653 1305.7041 46.9 0 R.AQTVILATGGYGR.A**

**227 – 247 2171.9805 2170.9732 2170.9194 24.8 0 R.AYASCTSAHTCTGDGGGMVLR.A**

**227 – 247 2188.0222 2187.0149 2186.9144 46.0 0 R.AYASCTSAHTCTGDGGGMVLR.A + Oxidation (M)**

**248 – 277 3268.5649 3267.5577 3267.5152 13.0 0 R.AGLPMQDMEFVQFHPTGIYGSGCLVTEGAR.G**

**278 – 290 1366.7006 1365.6933 1365.6161 56.6 0 R.GEGGYLVNSEGER.F**

**278 – 294 1929.9377 1928.9305 1928.8687 32.0 1 R.GEGGYLVNSEGERFMER.Y**

**390 – 417 2837.4170 2836.4097 2836.3596 17.7 1 K.KDGDDNAVIPGLMAIGEAACVSVHGANR.L**

**391 – 417 2709.3252 2708.3179 2708.2646 19.7 0 K.DGDDNAVIPGLMAIGEAACVSVHGANR.L**

**418 – 431 1489.8885 1488.8813 1488.8300 34.4 0 R.LGSNSLIDLVVFGR.A**

**441 – 461 2190.1177 2189.1104 2189.1804 -32.0 1 K.LTPNAKQPELPANSAELALGR.L**

**462 – 466 703.4163 702.4090 702.3449 91.2 0 R.LDHYR.Y**

**477 – 493 2063.0278 2062.0206 2061.9659 26.5 1 K.LREGMQHVMQSNCAVFR.T**

**479 – 493 1793.8605 1792.8532 1792.7807 40.4 0 R.EGMQHVMQSNCAVFR.T**

**508 – 520 1369.7867 1368.7795 1368.6998 58.2 0 K.VHSGITDIAVSDR.S**

**585 – 607 2806.4656 2805.4583 2805.4160 15.1 1 K.VKIEYRPVHDYTMTNDVQYIPPK.A**

**587 – 607 2579.3184 2578.3111 2578.2526 22.7 0 K.IEYRPVHDYTMTNDVQYIPPK.A**

**587 – 607 2595.3052 2594.2979 2594.2475 19.4 0 K.IEYRPVHDYTMTNDVQYIPPK.A + Oxidation (M)**

Spot ID: 3**

**Pyruvate dehydrogenase subunit beta [*Bradyrhizobium* sp. ORS 278] Mass:** 49126    **Score:** 48     **Matches:** 1(1)  **Sequences:** 1(1) **Protein sequence coverage:** 4%

**Monoisotopic mass of neutral peptide Mr(calc):** 1999.9963

**Fixed modifications:** Carbamidomethyl (C)

**Matches:** 58/375 fragment ions using 63 most intense peaks

**Start – End Observed Mr(expt) Mr(calc) ppm M Peptide**

**396 – 415 2000.7055 1999.6982 1999.9963 -149 0 AVTVEEGWAQSGVGAEIAAR.I**

Spot ID: 4*

**Pyruvate dehydrogenase subunit beta [*Bradyrhizobium japonicum* USDA 110]** **Mass: 48906 Score: 186 Calculated pI: 4.81 Mass values searched: 34 Mass values matched: 18**

**Protein sequence coverage: 50%**

**Start – End Observed Mr(expt) Mr(calc) ppm M Peptide**

**59 – 89 3049.4456 3048.4383 3048.6230 -60.6 0 K.ILIPEGTADVAVNTPIATILADGESAADLAK.A**

**158 – 176 2120.8264 2119.8191 2119.9408 -57.4 0 R.DADVFVMGEEVAEYQGAYK.V**

**177 – 188 1290.6313 1289.6241 1289.6980 -57.3 0 K.VTQGLLQEFGAK.R**

**177 – 189 1446.7168 1445.7095 1445.7991 -61.9 1 K.VTQGLLQEFGAKR.V**

**238 – 253 1806.7324 1805.7251 1805.8263 -56.0 0 K.TLYMSGGQMGCSIVFR.G**

**238 – 253 1822.7292 1821.7220 1821.8212 -54.5 0 K.TLYMSGGQMGCSIVFR.G + Oxidation (M)**

**262 – 281 2236.9241 2235.9168 2236.0549 -61.8 0 R.VAAQHSQDYSSWYSNVPGLK.V**

**282 – 292 1167.5577 1166.5505 1166.5972 -40.0 0 K.VVAPFSAADYK.G**

**297 – 322 2878.3218 2877.3145 2877.5025 -65.3 1 K.AAIRDPNPVIFLENEVLYGHTGEVPK.L**

**357 – 374 1927.8782 1926.8709 1926.9898 -61.7 1 K.AADELAKDGIEAEVIDLR.T**

**364 – 374 1229.5804 1228.5732 1228.6299 -46.2 0 K.DGIEAEVIDLR.T**

**375 – 389 1703.8007 1702.7934 1702.8924 -58.1 0 R.TLRPMDTETIVNSVK.K**

**375 – 390 1831.8820 1830.8747 1830.9873 -61.5 1 R.TLRPMDTETIVNSVKK.T**

**394 – 413 2000.8838 1999.8765 1999.9963 -59.9 0 R.AVTVEEGWAQSGVGAEIAAR.I**

**414 – 428 1724.7345 1723.7272 1723.8239 -56.1 0 R.IMENAFDYLDAPVAR.V**

**414 – 428 1740.7329 1739.7256 1739.8188 -53.6 0 R.IMENAFDYLDAPVAR.V + Oxidation (M)**

**429 – 444 1718.7858 1717.7785 1717.8709 -53.8 1 R.VSGKDVPMPYAANLEK.L**

**445 – 458 1368.6964 1367.6891 1367.7660 -56.2 0 K.LALPSAAEVVEAAK.A**

Spot ID: 5*

**ATP synthase F0F1 subunit beta [*Bradyrhizobium japonicum* USDA 110]**

**Mass: 50987 Score: 235 Calculated pI: 5.13 Mass values searched: 35 Mass values matched: 23**

**Protein sequence coverage: 69%**

**Start – End Observed Mr(expt) Mr(calc) ppm M Peptide**

**8 – 34 2877.6367 2876.6294 2876.5648 22.5 0 R.VTQVIGAVVDVQFEGHLPAILNSLETK.N**

**40 – 54 1651.0088 1650.0015 1649.9101 55.4 0 R.LVLEVAQHLGESTVR.T**

**55 – 66 1306.7419 1305.7347 1305.6599 57.3 0 R.TIAMDTTEGLVR.G**

**67 – 88 2225.2507 2224.2435 2224.1448 44.4 1 R.GQEVTDTGSPIRVPVGEGTLGR.I**

**89 – 109 2206.3035 2205.2962 2205.2005 43.4 1 R.IINVIGEPIDEAGPVKSEGLR.A**

**110 – 133 2615.3923 2614.3851 2614.3126 27.7 0 R.AIHQEAPTYTDQSTEAEILVTGIK.V**

**158 – 170 1454.9203 1453.9130 1453.8504 43.1 0 K.TVLIQELINNVAK.A**

**171 – 184 1406.7798 1405.7725 1405.6739 70.2 0 K.AHGGYSVFAGVGER.T**

**185 – 199 1837.9747 1836.9675 1836.8642 56.2 1 R.TREGNDLYHEFIESK.V**

**215 – 229 1646.8726 1645.8653 1645.7705 57.6 0 K.CALVFGQMNEPPGAR.A**

**215 – 229 1662.9689 1661.9616 1661.7654 118 0 K.CALVFGQMNEPPGAR.A + Oxidation (M)**

**247 – 259 1569.9067 1568.8995 1568.7988 64.2 0 K.GQDVLFFVDNIFR.F**

**260 – 273 1435.8486 1434.8414 1434.7467 66.0 0 R.FTQAGSEVSALLGR.I**

**274 – 294 2218.2212 2217.2139 2217.1100 46.9 0 R.IPSAVGYQPTLATDMGALQER.I**

**274 – 294 2234.2109 2233.2037 2233.1049 44.2 0 R.IPSAVGYQPTLATDMGALQER.I + Oxidation (M)**

**342 – 355 1490.8457 1489.8384 1489.7413 65.2 0 K.GIYPAVDPLDSTSR.M**

**356 – 371 1771.0149 1770.0076 1769.9134 53.2 0 R.MLSPLVVGEEHYAVAR.Q**

**356 – 371 1786.9959 1785.9886 1785.9083 44.9 0 R.MLSPLVVGEEHYAVAR.Q + Oxidation (M)**

**372 – 379 998.6386 997.6313 997.5669 64.6 0 R.QVQQVLQR.Y**

**382 – 405 2643.4863 2642.4791 2642.3836 36.1 1 K.ALQDIIAILGMDELSEEDKLTVAR.A**

**382 – 405 2659.4307 2658.4234 2658.3786 16.9 1 K.ALQDIIAILGMDELSEEDKLTVAR.A + Oxidation (M)**

**412 – 429 1980.0455 1979.0383 1978.9611 39.0 0 R.FMSQPFHVAEIFTGSPGK.F**

**442 – 468 2995.5610 2994.5538 2994.4684 28.5 1 K.GLVEGKYDHLPEAAFYMVGTIEEAVEK.G**

Spot ID: 6*

**ATP synthase F0F1 subunit beta [*Bradyrhizobium japonicum* USDA 110] Mass: 50987 Score: 148 Calculated pI:** 5.13 **Mass values searched:** 22 **Mass values matched:** 14

**Protein sequence coverage:** 41%

**Start – End Observed Mr(expt) Mr(calc) ppm M Peptide**

**40 – 54 1650.8137 1649.8064 1649.9101 -62.8 0 R.LVLEVAQHLGESTVR.T**

**67 – 88 2225.0085 2224.0013 2224.1448 -64.5 1 R.GQEVTDTGSPIRVPVGEGTLGR.I**

**89 – 109 2206.0715 2205.0643 2205.2005 -61.8 1 R.IINVIGEPIDEAGPVKSEGLR.A**

**110 – 133 2615.1343 2614.1270 2614.3126 -71.0 0 R.AIHQEAPTYTDQSTEAEILVTGIK.V**

**171 – 184 1406.6035 1405.5962 1405.6739 -55.2 0 K.AHGGYSVFAGVGER.T**

**185 – 199 1837.7925 1836.7852 1836.8642 -43.0 1 R.TREGNDLYHEFIESK.V**

**215 – 229 1646.6815 1645.6742 1645.7705 -58.5 0 K.CALVFGQMNEPPGAR.A**

**260 – 273 1435.6803 1434.6730 1434.7467 -51.3 0 R.FTQAGSEVSALLGR.I**

**274 – 294 2217.9958 2216.9886 2217.1100 -54.7 0 R.IPSAVGYQPTLATDMGALQER.I**

**274 – 294 2233.9697 2232.9625 2233.1049 -63.8 0 R.IPSAVGYQPTLATDMGALQER.I + Oxidation (M)**

**342 – 355 1490.7191 1489.7118 1489.7413 -19.8 0 K.GIYPAVDPLDSTSR.M**

**356 – 371 1770.8160 1769.8088 1769.9134 -59.1 0 R.MLSPLVVGEEHYAVAR.Q**

**356 – 371 1786.7938 1785.7865 1785.9083 -68.2 0 R.MLSPLVVGEEHYAVAR.Q + Oxidation (M)**

**372 – 379 998.5100 997.5027 997.5669 -64.3 0 R.QVQQVLQR.Y**

Spot ID: 7*

**ATP synthase F0F1 subunit beta [*Bradyrhizobium japonicum* USDA 110] Mass: 50987 Score: 84 Calculated pI:** 5.13 **Mass values searched: 17 Mass values matched: 8**

**Protein sequence coverage: 32%**

**Start – End Observed Mr(expt) Mr(calc) ppm M Peptide**

**40 – 54 1650.6738 1649.6666 1649.9101 -148 0 R.LVLEVAQHLGESTVR.T**

**67 – 88 2224.8237 2223.8165 2224.1448 -148 1 R.GQEVTDTGSPIRVPVGEGTLGR.I**

**89 – 109 2205.8889 2204.8816 2205.2005 -145 1 R.IINVIGEPIDEAGPVKSEGLR.A**

**110 – 133 2614.9333 2613.9261 2614.3126 -148 0 R.AIHQEAPTYTDQSTEAEILVTGIK.V**

**274 – 294 2217.8066 2216.7994 2217.1100 -140 0 R.IPSAVGYQPTLATDMGALQER.I**

**274 – 294 2233.7935 2232.7862 2233.1049 -143 0 R.IPSAVGYQPTLATDMGALQER.I + Oxidation (M)**

**301 – 336 3702.3806 3701.3733 3701.8425 -127 0 K.GSITSVQAIYVPADDLTDPAPATSFAHLDATTTLSR.S**

**356 – 371 1770.6730 1769.6657 1769.9134 -140 0 R.MLSPLVVGEEHYAVAR.Q**

Spot ID: 8*

**Aldehyde dehydrogenase [*Bradyrhizobium japonicum* USDA 110] Mass: 55297 Score: 134 Calculated pI: 6.04 Mass values searched: 49 Mass values matched: 18**

**Protein sequence coverage: 36%**

**Start – End Observed Mr(expt) Mr(calc) ppm M Peptide**

**12 – 17 718.3761 717.3688 717.3810 -16.9 0 K.VPFAER.Y**

**12 – 25 1612.7541 1611.7469 1611.8045 -35.8 1 K.VPFAERYDNFIGGK.F**

**18 – 33 1712.7277 1711.7204 1711.8934 -101 1 R.YDNFIGGKFVAPISGK.Y**

**52 – 68 1682.7347 1681.7275 1681.7907 -37.6 0 R.SDAQDVEAALDAAHAAK.A**

**69 – 79 1120.5135 1119.5063 1119.5309 -22.0 0 K.AGWGSTSVAER.A**

**86 – 96 1375.6204 1374.6131 1374.6561 -31.3 1 K.IADRMEENLER.L**

**86 – 96 1391.6479 1390.6407 1390.6510 -7.45 1 K.IADRMEENLER.L + Oxidation (M)**

**114 – 125 1338.6721 1337.6648 1337.7092 -33.1 0 R.AADIPLAIDHFR.Y**

**126 – 132 811.4214 810.4141 810.4388 -30.5 0 R.YFAGVVR.A**

**232 – 245 1451.6688 1450.6615 1450.7780 -80.3 1 R.IAKIAFTGETSTGR.L**

**235 – 245 1139.5311 1138.5239 1138.5619 -33.4 0 K.IAFTGETSTGR.L**

**306 – 315 1172.5809 1171.5737 1171.5986 -21.3 0 R.ALVHADIYDR.F**

**306 – 319 1735.7876 1734.7803 1734.8512 -40.8 1 R.ALVHADIYDRFMER.A**

**306 – 319 1751.7711 1750.7638 1750.8461 -47.0 1 R.ALVHADIYDRFMER.A + Oxidation (M)**

**351 – 364 1534.7913 1533.7840 1533.8402 -36.7 1 K.ILSYIDIGKQEGAK.V**

**372 – 394 2422.0559 2421.0486 2421.1601 -46.0 0 R.AELGGDLSGGFYVQPTVFEGHNK.M**

**397 – 413 1940.9390 1939.9317 1940.0295 -50.4 0 R.IFQEEIFGPVVSVTTFK.T**

**414 – 436 2408.0679 2407.0606 2407.1656 -43.6 0 K.TDDEALAIANDTLYGLGAGVWSR.D**

Spot ID: 9*

**Succinate-semialdehyde dehydrogenase [Bradyrhizobium japonicum USDA 110]** **Mass: 50087 Score: 192 Calculated pI: 5.30 Mass values searched: 32 Mass values matched: 18**

**Protein sequence coverage: 50%**

**Start – End Observed Mr(expt) Mr(calc) ppm M Peptide**

**2 – 27 2837.4084 2836.4012 2836.3443 20.0 0 M.AYATTYPYTGEVLETFPTATDAEVAR.A**

**28 – 40 1412.8073 1411.8000 1411.7360 45.3 0 R.AIDAAHAAFLAWR.E**

**90 – 106 2099.1357 2098.1285 2098.0735 26.2 0 K.IFEYYVQNAESLLRPEK.L**

**158 – 171 1484.8010 1483.7937 1483.7168 51.9 0 K.HASNVPQSAAAFER.L**

**175 – 190 1721.9541 1720.9468 1720.8784 39.8 0 R.EAGLPEGAFINLYATR.S**

**191 – 201 1271.7224 1270.7151 1270.6517 49.9 0 R.SQVETIINDPR.V**

**202 – 223 2008.1307 2007.1235 2007.0385 42.3 0 R.VHGVALTGSEDAGAVVAAQAGK.A**

**252 – 257 735.4373 734.4300 734.3864 59.3 0 K.WAVFGR.H**

**271 – 284 1654.9196 1653.9123 1653.8436 41.5 0 R.MIIVDAVYDEFLAR.Y**

**271 – 284 1670.9299 1669.9227 1669.8385 50.4 0 R.MIIVDAVYDEFLAR.Y + Oxidation (M)**

**285 – 293 1020.6171 1019.6098 1019.5512 57.5 1 R.YREGVAGLR.A**

**330 – 359 3116.6262 3115.6189 3115.5575 19.7 0 K.AEQVGPPVPNQGAFVQPTILTDVADDNPAR.Y**

**360 – 372 1678.8805 1677.8732 1677.8014 42.8 0 R.YWEFFGPVSMLFR.A**

**360 – 372 1694.8746 1693.8674 1693.7963 42.0 0 R.YWEFFGPVSMLFR.A + Oxidation (M)**

**382 – 400 1913.0090 1912.0018 1911.9214 42.0 0 R.IANDSPFGLGGSVFTSDTK.H**

**422 – 432 1173.7073 1172.7000 1172.6190 69.1 0 K.VEADLPFGGIR.R**

**422 – 433 1329.7911 1328.7839 1328.7201 48.0 1 K.VEADLPFGGIRR.S**

**453 – 463 1275.7578 1274.7505 1274.6871 49.8 1 K.LIDVVDIDARF.-**

Spot ID: 10*

**Phosphopyruvate hydratase [Bradyrhizobium japonicum USDA 110] Mass: 45314 Score: 188 Mass values searched: 30 Mass values matched: 17 Protein sequence coverage: 45%**

**Start – End Observed Mr(expt) Mr(calc) ppm M Peptide**

**2 – 10 971.6176 970.6103 970.5811 30.0 0 M.TAIIDIIGR.E**

**17 – 34 1839.9523 1838.9450 1838.9374 4.12 0 R.GNPTVEVDVVLEDGALGR.A**

**35 – 52 1722.8818 1721.8746 1721.8696 2.86 0 R.AAVPSGASTGAHEAVELR.D**

**35 – 56 2138.0525 2137.0452 2137.0400 2.46 1 R.AAVPSGASTGAHEAVELRDGDK.A**

**106 – 120 1457.8412 1456.8339 1456.8072 18.4 0 R.LGANAILGVSLACAK.A**

**121 – 132 1321.6777 1320.6705 1320.6496 15.8 0 K.AAANSLDMPLYR.Y**

**133 – 140 810.4434 809.4361 809.4032 40.7 0 R.YVGGTSAR.L**

**181 – 190 1164.6385 1163.6313 1163.5975 29.0 0 R.YGAEVFHTLK.S**

**233 – 250 1929.9324 1928.9251 1928.9190 3.17 0 K.AGSDIVIGLDCASTEFFK.D**

**233 – 253 2230.0767 2229.0694 2229.0624 3.15 1 K.AGSDIVIGLDCASTEFFKDGK.Y**

**271 – 278 920.5445 919.5372 919.5127 26.7 0 K.YLADLVAR.Y**

**307 – 321 1763.9154 1762.9081 1762.9036 2.55 1 K.CQLVGDDLFVTNVKR.L**

**338 – 356 2026.0751 2025.0678 2025.0565 5.60 0 K.VNQIGTLTETLAAVEMAHK.A**

**338 – 356 2042.0599 2041.0527 2041.0514 0.63 0 K.VNQIGTLTETLAAVEMAHK.A + Oxidation (M)**

**357 – 366 1108.5472 1107.5400 1107.5131 24.2 0 K.AGYTSVMSHR.S**

**367 – 388 2280.0779 2279.0706 2279.0587 5.22 0 R.SGETEDSTIADLAVATNCGQIK.T**

**401 – 406 806.4813 805.4740 805.4446 36.5 0 K.YNQLLR.I**

Spot ID: 11*

**Phosphopyruvate hydratase [Bradyrhizobium japonicum USDA 110] Mass: 45314 Score: 89 Calculated pI: 5.08 Mass values searched: 25 Mass values matched: 10**

**Protein sequence coverage: 31%**

**Start – End Observed Mr(expt) Mr(calc) ppm M Peptide**

**2 – 16 1684.9662 1683.9589 1683.9519 4.16 1 M.TAIIDIIGREILDSR.G**

**17 – 34 1839.9407 1838.9334 1838.9374 -2.19 0 R.GNPTVEVDVVLEDGALGR.A**

**121 – 132 1321.6802 1320.6729 1320.6496 17.7 0 K.AAANSLDMPLYR.Y**

**271 – 278 920.5314 919.5241 919.5127 12.4 0 K.YLADLVAR.Y**

**279 – 297 2233.0566 2232.0494 2231.9239 56.2 0 R.YPIVTIEDGMSEDDMDGWK.E + 2 Oxidation (M)**

**307 – 321 1763.8967 1762.8895 1762.9036 -8.04 1 K.CQLVGDDLFVTNVKR.L**

**338 – 356 2026.0603 2025.0530 2025.0565 -1.69 0 K.VNQIGTLTETLAAVEMAHK.A**

**338 – 356 2042.0420 2041.0347 2041.0514 -8.16 0 K.VNQIGTLTETLAAVEMAHK.A + Oxidation (M)**

**367 – 388 2280.0576 2279.0503 2279.0587 -3.67 0 R.SGETEDSTIADLAVATNCGQIK.T**

**401 – 406 806.4568 805.4495 805.4446 6.08 0 K.YNQLLR.I**

Spot ID: 12**

**Malate dehydrogenase** **[*Bradyrhizobium japonicum* USDA 110]** **Mass:** 34275    **Score:** 200    **Matches:** 1(1)  **Sequences:** 1(1) **Protein sequence coverage:** 6% **Monoisotopic mass of neutral peptide Mr(calc):** 2147.0357

**Fixed modifications:** Carbamidomethyl (C)

**Matches :** 73/382 fragment ions using 66 most intense peaks

**Start – End Observed Mr(expt) Mr(calc) ppm M Peptide**

**228 – 248 2147.9071 2146.8998 2147.0357 -63.3 0**

**K.TGSAFYAPAASAIAMAESYLR.D**

Spot ID: 13**

### Malate dehydrogenase [*Bradyrhizobium japonicum* USDA 110] Mass: **34275**    Score: **200**    Matches: **1(1)** Sequences: **1(1)** Protein sequence coverage: 6%

**Monoisotopic mass of neutral peptide Mr(calc):** 2147.0357

**Fixed modifications:** Carbamidomethyl (C)

**Matches :** 73/382 fragment ions using 66 most intense peaks

### Start – End Observed Mr(expt) Mr(calc) ppm M Peptide

### 228 – 248 2147.9071 2146.8998 2147.0357 -63.3 0 K.TGSAFYAPAASAIAMAESYLR.D

Spot ID: 14**

### Rieske iron-sulfur protein [*Bradyrhizobium japonicum* USDA 6] Mass: **15192**   Score: **158**    Matches: **1(1)** Sequences: **1(1)** Protein sequence coverage: 11%

**Monoisotopic mass of neutral peptide Mr(calc):** 1667.8115

**Fixed modifications:** Carbamidomethyl (C)

**Matches :** 35/294 fragment ions using 28 most intense peaks

**Start – End Observed Mr(expt) Mr(calc) ppm M Peptide**

**51 – 66 1668.6996 1667.6923 1667.8115 -71.4 0 R.AVNVASLPDPQSDEAR.V**

Spot ID: 15*

**Electron transfer flavoprotein large subunit [Bradyrhizobium japonicum USDA 110] Mass: 32186 Score: 110 Calculated pI: 5.14 Mass values searched: 16 Mass values matched: 7**

**Protein sequence coverage: 50%**

**Start – End Observed Mr(expt) Mr(calc) ppm M Peptide**

**56 – 94 3976.1545 3975.1473 3975.1720 -6.23 1 K.KVLLADGALYAHDLAEPLAALIVSLAPSYDAIVAPATSR.F**

**57 – 94 3848.0864 3847.0792 3847.0771 0.54 0 K.VLLADGALYAHDLAEPLAALIVSLAPSYDAIVAPATSR.F**

**117 – 137 2305.1023 2304.0950 2304.2114 -50.5 0 K.VVAPDTYERPIYAGNAIQTVK.S**

**149 – 184 3394.3284 3393.3211 3393.6212 -88.4 0 R.TSTFAAAGEGGSAPVESVQAAADPGLSSFVGEEVAK.S**

**212 – 228 1716.8284 1715.8211 1715.9206 -58.0 1 K.YIEPLADKLGAGVGASR.A**

**229 – 247 1947.7278 1946.7205 1946.9123 -98.5 0 R.AAVDAGYAPNDWQVGQTGK.V**

**248 – 273 2653.3032 2652.2959 2652.4309 -50.9 1 K.VVAPELYVAVGISGAIQHLAGMKDSK.V**

Spot ID: 16**

Ferredoxin NADP+ reductase [*Bradyrhizobium japonicum* USDA 6] **Mass:** 29065    **Score:** 114    **Matches:** 1(1)  **Sequences:** 1(1) Protein sequence coverage: 6% **Monoisotopic mass of neutral peptide Mr(calc):** 1872.9370

**Fixed modifications:** Carbamidomethyl (C)

**Matches :** 94/313 fragment ions using 114 most intense peaks

**Start – End Observed Mr(expt) Mr(calc) ppm M Peptide**

**236 – 252 1873.8821 1872.8748 1872.9370 -33.2 0 R.DFIEGSGNKPGHFVIEK.A**

Spot ID: 17**

**Inositol monophosphatase** **[*Bradyrhizobium japonicum* USDA 110] Mass:** 28290    **Score:** 90     **Matches:** 1(1)  **Sequences:** 1(1)  **Protein sequence coverage:** 6% **Monoisotopic mass of neutral peptide Mr(calc):** 1741.7656

**Fixed modifications:** Carbamidomethyl (C)

**Matches :** 51/314 fragment ions using 55 most intense peaks

**Start – End Observed Mr(expt) Mr(calc) ppm M Peptide**

### 127 – 143 1743.0000 1741.9927 1741.7656 130 1 R.FSGDNGSANYKGPSGER.R

Spot ID: 18**

**Sugar kinase [*Bradyrhizobium japonicum* USDA 110] Mass:** 35336    **Score:** 156    **Matches:** 1(1)  **Sequences:** 1(1) **Protein sequence coverage:** 5% **Monoisotopic mass of neutral peptide Mr(calc):** 1866.8418

**Fixed modifications:** Carbamidomethyl (C)

**Matches :** 49/311 fragment ions using 52 most intense peaks

### Start – End Observed Mr(expt) Mr(calc) ppm M Peptide

### 110 – 127 1867.7033 1866.6960 1866.8418 -78.1 0 K.DGPATGCSYILVTGDGER.T

Spot ID: 19**

**Sugar ABC transporter substrate-binding protein** **[*Bradyrhizobium japonicum* USDA 110] Mass:** 38378    **Score:** 67     **Matches:** 1(1)  **Sequences:** 1(1) Protein sequence coverage: 7% **Monoisotopic mass of neutral peptide Mr(calc):** 2501.2108

**Fixed modifications:** Carbamidomethyl (C)

**Matches :** 123/496 fragment ions using 136 most intense peaks

**Start – End Observed Mr(expt) Mr(calc) ppm M Peptide**

**252 – 276 2502.0237 2501.0164 2501.2108 -77.7 0 K.GVGYGSADQPMPVISGQDAEVPSIK.A**

Spot ID: 20**

**6-phosphogluconate dehydrogenase [*Bradyrhizobium* *japonicum* USDA 6]** **Mass:** 35930    **Score:** 109    **Matches:** 1(1)  **Sequences:** 1(1) **Protein sequence coverage:** 5% **Monoisotopic mass of neutral peptide Mr(calc):** 1886.9276

**Fixed modifications:** Carbamidomethyl (C)

**Matches:** 86/312 fragment ions using 108 most intense peaks

**Start – End Observed Mr(expt) Mr(calc) ppm M Peptide**

**112 – 129 1887.8734 1886.8661 1886.9276 -32.6 0 R.GIHYVDVGTSGGVWGLDR.G**

Spot ID: 21**

**N-(5'-phosphoribosyl) anthranilate isomerase [*Bradyrhizobium japonicum* USDA 110]** **Mass:** 23966    **Score:** 137    **Matches:** 1(1)  **Sequences:** 1(1)

Protein sequence coverage: 9% **Monoisotopic mass of neutral peptide Mr(calc):** 2126.1372

**Fixed modifications:** Carbamidomethyl (C)

**Matches :** 56/403 fragment ions using 40 most intense peaks

**Start – End Observed Mr(expt) Mr(calc) ppm M Peptide**

**106 – 127 2127.0669 2126.0596 2126.1372 -36.5 0**

**K.AVPVATSADLAVLPGYAAVADR.I**

Spot ID: 22**

**Isopropylmalate isomerase small subunit [*Bradyrhizobium japonicum* USDA 110] Mass:** 22781    **Score:** 173    **Matches:** 1(1)  **Sequences:** 1(1)

**Protein sequence coverage:** 9% **Monoisotopic mass of neutral peptide Mr(calc):** 2227.0182

**Fixed modifications:** Carbamidomethyl (C)

**Matches :** 60/351 fragment ions using 56 most intense peaks

**Start – End Observed Mr(expt) Mr(calc) ppm M Peptide**

**48 – 66 2227.9968 2226.9895 2227.0182 -12.9 1**

**R.YKDDGSENPDFVLNQPAYR.N**

Spot ID: 23**

**Amino acid ABC transporter substrate-binding protein** **[*Bradyrhizobium japonicum* USDA 110] Mass:** 36860    **Score:** 75     **Matches:** 1(1) **Sequences:** 1(1)

Protein sequence coverage: 6% **Monoisotopic mass of neutral peptide Mr(calc):** 2289.1754

**Fixed modifications:** Carbamidomethyl (C)

**Matches :** 33/387 fragment ions using 43 most intense peaks

**Start – End Observed Mr(expt) Mr(calc) ppm M Peptide**

**274 – 294 2289.9010 2288.8937 2289.1754 -123 1 R.VLGSDGNFGEQLGLTKDWVVR.I**

Spot ID: 24**

**Diaminopimelate epimerase [*Bradyrhizobium japonicum* USDA 110]** **Mass:** 31803    **Score:** 109    **Matches:** 1(1)  **Sequences:** 1(1)  **Protein sequence coverage:** 7% **Monoisotopic mass of neutral peptide Mr(calc):** 2227.1783

**Fixed modifications:** Carbamidomethyl (C)

**Matches :** 46/390 fragment ions using 38 most intense peaks

**Start – End Observed Mr(expt) Mr(calc) ppm M Peptide**

**38 – 58 2227.9035 2226.8962 2227.1783 -127 0 R.AVASAQGGVPYDQLMVLQKPR.L**

Spot ID: 25**

**Hypothetical protein blr3064 [*Bradyrhizobium japonicum* USDA 110]** **Mass:** 50837    **Score:** 122    **Matches:** 1(1)  **Sequences:** 1(1)

**Protein sequence coverage:** 3% **Monoisotopic mass of neutral peptide Mr(calc):** 1656.8042

**Fixed modifications:** Carbamidomethyl (C)

**Matches :** 38/282 fragment ions using 40 most intense peaks

**Start – End Observed Mr(expt) Mr(calc) ppm M Peptide**

**219 – 234 1657.5790 1656.5717 1656.8042 -140 0 R.DLHSGVFGGSAMNPIR.V**

Spot ID: 26**

**Glutamine synthetase [Bradyrhizobium japonicum USDA 110] Mass: 52623 Score: 91 Calculated pI: 5.44 Mass values searched: 32 Mass values matched: 11**

**Protein sequence coverage: 30%**

**Start – End Observed Mr(expt) Mr(calc) ppm M Peptide**

**119 – 141 2519.8386 2518.8313 2519.1679 -134 0 K.SMGVGDTVFVGPEAEFFVFDDVR.F**

**119 – 141 2535.8125 2534.8052 2535.1629 -141 0 K.SMGVGDTVFVGPEAEFFVFDDVR.F + Oxidation (M)**

**142 – 152 1262.4121 1261.4048 1261.5728 -133 0 R.FSSSPYNTGFR.L**

**153 – 174 2404.7542 2403.7469 2404.0779 -138 0 R.LDSSELPTNTDTEYEGGNLGHR.V**

**177 – 194 2021.6947 2020.6874 2020.9677 -139 1 R.TKGGYFPVPPQDSVQDMR.S**

**179 – 194 1792.5900 1791.5827 1791.8250 -135 0 K.GGYFPVPPQDSVQDMR.S**

**241 – 252 1488.5404 1487.5331 1487.7343 -135 0 K.YCIHQVAHIYGK.T**

**288 – 304 1952.7096 1951.7023 1951.9713 -138 0 K.YADLSETCLHYIGGIIK.H**

**308 – 322 1683.6021 1682.5948 1682.8376 -144 1 K.AINAFTNPSTNSYKR.L**

**323 – 338 1718.7008 1717.6935 1717.9403 -144 0 R.LVPGYEAPVLLAYSAR.N**

**346 – 353 876.3703 875.3630 875.4752 -128 0 R.IPYTASPK.A**

Spot ID: 27**

**L-asparaginase [*Bradyrhizobium japonicum* USDA 110] Mass:** 39549    **Score:** 128    **Matches:** 1(1)  **Sequences:** 1(1)

**Protein sequence coverage:** 6% **Monoisotopic mass of neutral peptide Mr(calc):** 2070.0746

**Fixed modifications:** Carbamidomethyl (C)

**Matches :** 65/374 fragment ions using 62 most intense peaks

**Start – End Observed Mr(expt) Mr(calc) ppm M Peptide**

**115 – 133 2071.1621 2070.1548 2070.0746 38.8 0 K.QIANTLSGTPFAPLEEQVR.R**

Spot ID: 28**

**Phosphoserine phosphatase** **[*Bradyrhizobium japonicum* USDA 110]** **Mass:** 32322    **Score:** 62     **Matches:** 1(1)  **Sequences:** 1(1) **Protein** **sequence coverage:** 3% **Monoisotopic mass of neutral peptide Mr(calc):** 1159.5873

**Fixed modifications:** Carbamidomethyl (C)

**Matches :** 10/140 fragment ions using 9 most intense peaks

Start – End Observed Mr(expt) Mr(calc) ppm M Peptide

125 – 134 1160.4768 1159.4695 1159.5873 -102 0 R.GEIEFEPALR.E

### Spot ID: 29**

### Acetylglutamate kinase [Bradyrhizobium japonicum USDA 110] Mass: **31294**    Score: **108**    Matches: **1(1)**  Sequences: **1(1)**

**Protein sequence coverage:** 4% **Monoisotopic mass of neutral peptide Mr(calc):** 1260.6826

**Fixed modifications:** Carbamidomethyl (C

**Matches :** 50/188 fragment ions using 44 most intense peaks

**Start – End Observed Mr(expt) Mr(calc) ppm M Peptide**

**77 – 88 1261.6247 1260.6174 1260.6826 -51.7 0 R.LGIQSEFAAGLR.I**

Spot ID: 30**

**Hypothetical protein Blr5678 [*Bradyrhizobium japonicum* USDA 110] Mass: 33744    Score: 121    Matches: 1(1)  Sequences: 1(1)**

**Protein sequence coverage:** 6% **Monoisotopic mass of neutral peptide Mr(calc):** 2073.1106

**Fixed modifications:** Carbamidomethyl (C)

**Matches :** 87/388 fragment ions using 59 most intense peaks

**Start – End Observed Mr(expt) Mr(calc) ppm M Peptide**

**21 – 41 2074.2524 2073.2451 2073.1106 64.9 0 K.VASGTTAIIFDSPAVAAIDVR.G**

Spot ID: 31**

**Thiamine-phosphate pyrophosphorylase** **[*Bradyrhizobium japonicum* USDA 110] Mass:** 22484    **Score:** 128    **Matches:** 1(1)  **Sequences:** 1(1)

Protein sequence coverage: 7% **Monoisotopic mass of neutral peptide Mr(calc):** 1699.8417

**Fixed modifications:** Carbamidomethyl (C)

**Matches :** 30/252 fragment ions using 29 most intense peaks

**Start – End Observed Mr(expt) Mr(calc) ppm M Peptide**

**80 – 94 1700.8084 1699.8011 1699.8417 -23.9 0 K.YLHLGQEDLADADLK.A**

Spot ID: 32*

**S-adenosyl-L-homocysteine hydrolase [Bradyrhizobium japonicum USDA 110]**

**Mass: 52318 Score: 90 Calculated pI: 6.00 Mass values searched: 22**

**Mass values matched: 9 Protein sequence coverage: 31%**

**Start – End Observed Mr(expt) Mr(calc) ppm M Peptide**

**24 – 39 1749.0002 1747.9930 1747.8484 82.7 0 K.ELSLAETEMPGLMATR.E**

**24 – 45 2436.3059 2435.2986 2435.1712 52.3 1 K.ELSLAETEMPGLMATREEFGPK.Q**

**107 – 119 1576.8209 1575.8136 1575.7093 66.2 0 K.GETLTEYWDYTAK.L**

**211 – 227 1799.1000 1798.0927 1797.9625 72.4 0 K.AGTLLWPAINVNDSVTK.S**

**228 – 237 1259.7205 1258.7132 1258.5764 109 1 K.SKFDNLYGCR.E**

**256 – 274 1793.0524 1792.0451 1791.9302 64.1 1 K.VAMVAGFGDVGKGSAASLR.Q**

**280 – 309 3281.7905 3280.7833 3280.5124 82.6 0 R.VMVSEVDPICALQAAMEGYEVVTMEDAAPR.A**

**310 – 329 2245.2859 2244.2786 2244.1572 54.1 1 R.ADIFVTATGNKDIITIEHMR.A**

**335 – 353 2140.2161 2139.2088 2139.0895 55.8 0 R.AIVCNIGHFDNEIQIAGLR.N**

Spot ID: 33*

**S-adenosylmethionine synthetase [Bradyrhizobium japonicum USDA 110] Mass: 43613 Score: 165 Calculated pI: 5.88 Mass values searched: 29 Mass values matched: 14**

**Protein sequence coverage: 40%**

**Start – End Observed Mr(expt) Mr(calc) ppm M Peptide**

**3 – 23 2384.1187 2383.1114 2383.0750 15.2 1 R.ASYLFTSESVSEGHPDKVCDR.I**

**24 – 34 1369.7623 1368.7551 1368.6925 45.7 0 R.ISDEIVDLFYR.E**

**39 – 47 1055.6034 1054.5961 1054.5560 38.1 0 K.AGIDPWQIR.A**

**48 – 66 2005.0885 2004.0812 2004.0310 25.1 1 R.AACETLATTNKVVIAGETR.G**

**70 – 81 1330.7480 1329.7408 1329.6888 39.1 0 K.SVTNEQIEGVVR.A**

**82 – 97 1891.9828 1890.9755 1890.9264 26.0 1 R.AAIKDIGYEQEGFHWK.T**

**86 – 97 1508.7520 1507.7447 1507.6732 47.4 0 K.DIGYEQEGFHWK.T**

**195 – 213 2144.1882 2143.1810 2143.1386 19.8 0 R.EIVVSHQHLVPDLTSSQVR.D**

**214 – 221 990.5685 989.5613 989.5182 43.5 0 R.DIVEPYVR.E**

**233 – 242 1166.6514 1165.6441 1165.6244 16.9 0 K.TIWHINPTGK.F**

**243 – 257 1495.7803 1494.7730 1494.7103 41.9 0 K.FYIGGPDGDAGLTGR.K**

**243 – 258 1623.8687 1622.8614 1622.8053 34.6 1 K.FYIGGPDGDAGLTGRK.I**

**355 – 366 1454.8458 1453.8385 1453.7790 41.0 0 R.SHLDLNRPIYAR.T**

**367 – 375 995.5172 994.5099 994.4621 48.1 0 R.TSAYGHFGR.T**

Spot ID: 34**

**S-Adenosylmethionine synthetase [*Bradyrhizobium japonicum*** **USDA 110]** **Mass:** 43613    **Score:** 109    **Matches:** 1(1) **Sequences:** 1(1)

**Protein sequence coverage:** 4% **Monoisotopic mass of neutral peptide Mr(calc):** 2143.1386

**Fixed modifications:** Carbamidomethyl (C)

**Matches :** 51/359 fragment ions using 52 most intense peaks

**Start – End Observed Mr(expt) Mr(calc) ppm M Peptide**

**195 – 213 2143.8931 2142.8858 2143.1386 -118 0 R.EIVVSHQHLVPDLTSSQVR.D**

Spot ID: 35**

**Delta-aminolevulinic acid dehydratase/ Porphobilinogen synthase [*Bradyrhizobium japonicum* USDA 110]** **Mass:** 38843    **Score:** 69     **Matches:** 1(1)  **Sequences:** 1(1)

Protein sequence coverage: 2% **Monoisotopic mass of neutral peptide Mr(calc):** 1177.5556

**Fixed modifications:** Carbamidomethyl (C)

**Matches :** 40/128 fragment ions using 50 most intense peaks

**Start – End Observed Mr(expt) Mr(calc) ppm M Peptide**

**222 – 231 1178.3949 1177.3876 1177.5556 -143 0 K.YASAFYGPFR.D**

Spot ID: 36**

**Hypothetical protein blr3798 [*Bradyrhizobium japonicum* USDA 110]** **Mass:** 27452    **Score:** 116    **Matches:** 1(1)  **Sequences:** 1(1)

**Protein sequence coverage:** 5% **Monoisotopic mass of neutral peptide Mr(calc):** 1639.7631

**Fixed modifications:** Carbamidomethyl (C)

**Matches :** 64/208 fragment ions using 62 most intense peaks

**Start – End Observed Mr(expt) Mr(calc) ppm M Peptide**

**99 – 112 1640.6289 1639.6216 1639.7631 -86.3 0 R.IEYYEYVGTGHGPR.I**

Spot ID: 37**

**Hypothetical protein Bll4565** **[*Bradyrhizobium japonicum* USDA 110] Mass:** 24885    **Score:** 127    **Matches:** 1(1)  **Sequences:** 1(1) **Protein sequence coverage:** 6% **Monoisotopic mass of neutral peptide Mr(calc):** 1774.8373

**Fixed modifications:** Carbamidomethyl (C)

**Matches :** 44/278 fragment ions using 39 most intense peaks

**Start – End Observed Mr(expt) Mr(calc) ppm M Peptide**

**60 – 75 1775.8153 1774.8080 1774.8373 -16.5 0 R.EDLATPESWSSPISTR.T**

Spot ID: 38**

**Phosphoribosylaminoimidazole-succinocarboxamide synthase [*Bradyrhizobium japonicum* USDA 110]** **Mass:** 33736    **Score:** 132    **Matches:** 1(1)  **Sequences:** 1(1)

**Protein sequence coverage:** 4% **Monoisotopic mass of neutral peptide Mr(calc):** 1722.8941

**Fixed modifications:** Carbamidomethyl (C)

**Matches :** 42/274 fragment ions using 45 most intense peaks

**Start – End Observed Mr(expt) Mr(calc) ppm M Peptide**

**202 – 216 1723.7791 1722.7718 1722.8941 -71.0 1 R.EQGIIIADTKFEFGR.D**

Spot ID: 39*

**Phosphoribosylaminoimidazole-succinocarboxamide synthase [*Bradyrhizobium japonicum* USDA 110]** **Mass:** 33736 **Score:** 59     **Matches:** 1(1)  **Sequences:** 1(1)

**Protein sequence coverage:** 7% **Monoisotopic mass of neutral peptide Mr(calc):** 2085.8763

**Fixed modifications:** Carbamidomethyl (C)

**Matches:** 26/323 fragment ions using 43 most intense peaks

**Start – End Observed Mr(expt) Mr(calc) ppm M Peptide**

**34 – 52 2086.7476 2085.7403 2085.8763 -65.2 0 R.SATPDYGWLSEESADDSTR.L**

Spot ID: 40*

**3-oxoacyl-ACP synthase [*Bradyrhizobium japonicum* USDA 110] Mass: 44874 Score: 173 Calculated pI: 5.56 Mass values searched: 19 Mass values matched: 13**

**Protein sequence coverage: 50%**

**Start – End Observed Mr(expt) Mr(calc) ppm M Peptide**

**44 – 54 1246.6682 1245.6609 1245.7081 -37.9 1 R.TITRFPVDGLK.T**

**55 – 77 2404.0405 2403.0333 2403.1265 -38.8 0 K.TTMAGTVDFVSVDPFSSTGLSER.M**

**122 – 135 1625.7908 1624.7835 1624.8461 -38.5 1 R.AVGKLDFTYDDLLR.I**

**142 – 148 933.4088 932.4015 932.4253 -25.5 0 K.YSAYHHR.F**

**166 – 190 2446.1521 2445.1448 2445.2533 -44.4 0 K.GSPISLSTACASGATSIQLGVEAIR.R**

**191 – 213 2415.0823 2414.0750 2414.1860 -46.0 1 R.RGETDAALCVATDGTVNPEALVR.F**

**214 – 236 2462.1667 2461.1595 2461.2601 -40.9 0 R.FSLLSALSTQNDPPQAASRPFSK.N**

**237 – 261 2598.1694 2597.1622 2597.2544 -35.5 1 K.NRDGFVMAEGAGALVLESYEAATAR.G**

**239 – 261 2328.0203 2327.0130 2327.1103 -41.8 0 R.DGFVMAEGAGALVLESYEAATAR.G**

**265 – 281 1844.8624 1843.8552 1843.9251 -37.9 0 K.ILGVIAGCGELTDSFHR.T**

**374 – 395 2443.1362 2442.1290 2442.2683 -57.0 0 R.IPPTINYETPDPTILFDVVGNK.A**

**401 – 425 2567.1926 2566.1854 2566.2850 -38.8 1 R.VTAVMSNSFGFGGQNASLILTREPA.-**

**401 – 425 2583.1846 2582.1773 2582.2799 -39.7 1 R.VTAVMSNSFGFGGQNASLILTREPA.- + Oxidation (M)**

Spot ID: 41**

**4-Hydroxy-3-methylbut-2-enyl diphosphate reductase [*Bradyrhizobium japonicum* USDA 110]**   **Mass:** 35239    **Score:** 53     **Matches:** 1(1)  **Sequences:** 1(1)

**Protein sequence coverage:** 2% **Monoisotopic mass of neutral peptide Mr(calc):** 923.4865

**Fixed modifications:** Carbamidomethyl (C)

**Matches :** 35/80 fragment ions using 40 most intense peaks

**Start – End Observed Mr(expt) Mr(calc) ppm M Peptide**

**35 – 42 924.4634 923.4561 923.4865 -32.9 0 K.YGAPVYVR.H**

Spot ID: 42**

**Nitrogenase iron protein ACP S-malonyl transferase   [*Bradyrhizobium japonicum* USDA 110] Mass:** 32462    **Score:** 82     **Matches:** 1(1)  **Sequences:** 1(1)

Protein sequence coverage: 3% **Monoisotopic mass of neutral peptide Mr(calc):** 1233.6353

**Fixed modifications:** Carbamidomethyl (C)

**Matches:** 39/181 fragment ions using 35 most intense peaks

**Start – End Observed Mr(expt) Mr(calc) ppm M Peptide**

**75 – 86 1234.5063 1233.4990 1233.6353 -111 0 R.VLEAEAGFSVGR.D**

Spot ID: 43**

**Enoyl CoA hidratase   [*Bradyrhizobium japonicum* USDA 110]** **Mass:** 27829    **Score:** 115    **Matches:** 1(1)  **Sequences:** 1(1)

**Protein sequence coverage:** 6% **Monoisotopic mass of neutral peptide Mr(calc):** 1846.8196

**Fixed modifications:** Carbamidomethyl (C)

**Matches :** 24/277 fragment ions using 24 most intense peaks

**Start – End Observed Mr(expt) Mr(calc) ppm M Peptide**

**75 – 91 1847.6960 1846.6887 1846.8196 -70.9 0 K.GFIDMFSEDFAAIGGDR.V**

Spot ID: 44**

**4-Diphosphocytidyl-2-C-methyl-D-erythritol kinase [*Bradyrhizobium japonicum* USDA 110**] **Mass:** 31076    **Score:** 85     **Matches:** 1(1)  **Sequences:** 1(1)

**Protein sequence coverage:** 5% **Monoisotopic mass of neutral peptide Mr(calc):** 1690.8712

**Fixed modifications:** Carbamidomethyl (C)

**Matches :** 36/269 fragment ions using 25 most intense peaks

**Start – End Observed Mr(expt) Mr(calc) ppm M Peptide**

**134 – 149 1692.0000 1690.9927 1690.8712 71.8 0 K.VALATGADVPVCLYSR.A**

Spot ID: 45**

**ABC transporter molybdenum-binding protein** **[Bradyrhizobium japonicum USDA 110] Mass:** 27290    **Score:** 141    **Matches:** 1(1)  **Sequences:** 1(1)

**Protein sequence coverage:** 9% **Monoisotopic mass of neutral peptide Mr(calc):** 2438.3210

**Fixed modifications:** Carbamidomethyl (C)

**Matches:** 63/442 fragment ions using 60 most intense peaks

**Start – End Observed Mr(expt) Mr(calc) ppm M Peptide**

**205 – 228 2439.2336 2438.2263 2438.3210 -38.8 0 K.IVGTFPADSHPAIIYPVAATVTAK.G**

Spot ID: 46**

**Nitrogenase iron protein [*Bradyrhizobium japonicum* USDA 110] Mass:** 31826    **Score:** 101    **Matches:** 1(1) **Sequences:** 1(1)

**Protein sequence coverage:** 4% **Monoisotopic mass of neutral peptide Mr(calc):** 1433.8395

**Fixed modifications:** Carbamidomethyl (C)

**Matches:** 66/191 fragment ions using 58 most intense peaks

**Start – End Observed Mr(expt) Mr(calc) ppm M Peptide**

**203 – 214 1434.6967 1433.6894 1433.8395 -105 1 K.KLGTQLIYFVPR.D**

Spot ID: 47**

**Thiol oxidoreductase FrnE [*Bradyrhizobium japonicum* USDA 110] Mass:** 24357    **Score:** 171    **Matches:** 1(1)  **Sequences:** 1(1)

**Protein sequence coverage:** 8% **Monoisotopic mass of neutral peptide Mr(calc):** 2000.0942

**Fixed modifications:** Carbamidomethyl (C)

**Matches:** 62/327 fragment ions using 52 most intense peaks

**Start – End Observed Mr(expt) Mr(calc) ppm M Peptide**

**80 – 98 2001.0907 2000.0834 2000.0942 -5.39 0 R.VVAAASEEGLVYKPELVAR.Q**

Spot ID: 48*

**Elongation factor G [*Bradyrhizobium japonicum* USDA 110] Mass: 77966    Score: 92**

**Calculated pI: 5.32 Number of mass values searched: 25 Number of mass values matched: 12 Protein Sequence Coverage: 22%**

**Start - End Observed Mr(expt) Mr(calc) ppm M Peptide**

**82 - 99 2102.1330 2101.1257 2101.0480 37 0**

**R.FNIIDTPGHVDFTIEVER.S**

**259 - 269 1342.6320 1341.6247 1341.5999 19 0**

**K.FHPMFCGTAFK.N**

**321 - 334 1567.7840 1566.7767 1566.7865 -6 0**

**K.IMNDPFVGSLTFAR.I**

**321 - 334 1583.7820 1582.7747 1582.7814 -4 0**

**K.IMNDPFVGSLTFAR.I Oxidation (M)**

**387 - 405 2158.1730 2157.1657 2157.0987 31 0**

**K.ETTTGDTLCDPLKPVILER.M**

**435 - 443 1005.6120 1004.6047 1004.4927 112 0**

**R.LAAEDPSFR.V**

**444 - 469 2839.6300 2838.6227 2838.4433 63 1**

**R.VKTDEESGQTIIAGMGELHLDIIVDR.M**

**446 - 469 2612.1530 2611.1457 2611.2799 -51 0**

**K.TDEESGQTIIAGMGELHLDIIVDR.M**

**473 - 488 1750.9020 1749.8947 1749.9050 -6 1**

**R.EFKVEASVGAPQVAYR.E**

**569 - 591 2434.5560 2433.5487 2433.2176 136 0**

**K.ATLIDGAFHDVDSSVLAFEIASR.A**

**612 - 631 2176.2310 2175.2237 2175.0696 71 0**

**K.VEVVTPEDYVGDVIGDLNSR.R**

**612 - 632 2332.2660 2331.2587 2331.1707 38 1**

**K.VEVVTPEDYVGDVIGDLNSRR.G**

Spot ID: 49*

**Elongation factor G [*Bradyrhizobium japonicum* USDA 110] Mass: 77966   Score: 104 Calculated pI: 5.32 Number of mass values searched: 36 Number of mass values matched: 16**

**Protein Sequence Coverage: 29%**

**Start - End Observed Mr(expt) Mr(calc) ppm M Peptide**

**81 - 99 2258.2840 2257.2767 2257.1491 57 1**

**R.RFNIIDTPGHVDFTIEVER.S**

**82 - 99 2102.0750 2101.0677 2101.0480 9 0**

**R.FNIIDTPGHVDFTIEVER.S**

**103 - 126 2538.5650 2537.5577 2537.3126 97 0**

**R.VLDGAIALLDANAGVEPQTETVWR.Q**

**179 - 192 1642.8880 1641.8807 1641.8912 -6 0**

**K.GVIDLIEMNALIWR.D**

**179 - 192 1658.8920 1657.8847 1657.8861 -1 0**

**K.GVIDLIEMNALIWR.D**  Oxidation (M)

**220 - 247 3145.4270 3144.4197 3144.5536 -43 0**

**K.LIETVVDIDEAAMEAYLEGILPDNDQIR.A**

**259 - 269 1342.6140 1341.6067 1341.5999 5 0**

**K.FHPMFCGTAFK.N**

**321 - 334 1567.7760 1566.7687 1566.7865 -11 0**

**K.IMNDPFVGSLTFAR.I**

**321 - 334 1583.7760 1582.7687 1582.7814 -8 0**

**K.IMNDPFVGSLTFAR.I**  Oxidation (M)

**387 - 405 2158.1550 2157.1477 2157.0987 23 0**

**K.ETTTGDTLCDPLKPVILER.M**

**406 - 420 1740.8890 1739.8817 1739.9168 -20 0**

**R.MEFPEPVIQIAIEPK.T**

**435 - 443 1005.5580 1004.5507 1004.4927 58 0**

**R.LAAEDPSFR.V**

**504 - 513 1008.5490 1007.5417 1007.4785 63 0**

**K.QSGGTGQFAR.V**

**569 - 591 2434.4160 2433.4087 2433.2176 79 0**

**K.ATLIDGAFHDVDSSVLAFEIASR.A**

**612 - 631 2176.1700 2175.1627 2175.0696 43 0**

**K.VEVVTPEDYVGDVIGDLNSR.R**

**612 - 632 2332.1570 2331.1497 2331.1707 -9 1**

**K.VEVVTPEDYVGDVIGDLNSRR.G**

Spot ID: 50*

**GTP-binding tyrosin phosphorylated protein [*Bradyrhizobium japonicum* USDA 110] Mass: 67131 Score: 116 Calculated pI: 5.49 Mass values searched: 43 Mass values matched: 17**

**Protein sequence coverage: 32%**

**Start – End Observed Mr(expt) Mr(calc) ppm M Peptide**

**17 – 31 1706.8341 1705.8268 1705.9363 -64.2 1 K.TTLVDKLLQQSGTFR.E**

**23 – 31 1049.5166 1048.5093 1048.5665 -54.6 0 K.LLQQSGTFR.E**

**68 – 85 1911.8038 1910.7966 1910.9123 -60.6 0 R.VNIVDTPGHADFGGEVER.I**

**181 – 191 1303.6339 1302.6266 1302.7006 -56.8 0 K.GMEPLFDLILR.H**

**181 – 191 1319.6534 1318.6462 1318.6955 -37.4 0 K.GMEPLFDLILR.H + Oxidation (M)**

**192 – 204 1435.6516 1434.6443 1434.7256 -56.6 0 R.HVAPPTVEEGPFR.M**

**205 – 218 1547.7318 1546.7245 1546.8177 -60.2 0 R.MIGTILEANPYLGR.I**

**205 – 218 1563.7147 1562.7074 1562.8126 -67.3 0 R.MIGTILEANPYLGR.I + Oxidation (M)**

**262 – 280 1883.8676 1882.8603 1882.9888 -68.2 0 R.TPLDEAEAGDIVAIAGLTK.G**

**353 – 373 2364.1213 2363.1141 2363.1824 -28.9 1 K.DAMEVSGRGELQLAILIETMR.R + 2 Oxidation (M)**

**375 – 385 1276.5981 1275.5909 1275.6571 -51.9 0 R.EGFELSVSRPR.V**

**440 – 447 966.4965 965.4892 965.5334 -45.8 0 R.LVFYAPTR.G**

**469 – 483 1735.7850 1734.7778 1734.8842 -61.3 1 R.LFHGYAPYKGEIQGR.R**

**504 – 517 1658.7130 1657.7057 1657.7956 -54.2 1 K.LEDRGPMMIEPGWK.V**

**508 – 520 1535.7188 1534.7115 1534.7676 -36.6 1 R.GPMMIEPGWKVYK.G**

**521 – 539 2152.9724 2151.9651 2152.0946 -60.2 1 K.GMIVGEHTRDNDLEINVLK.G**

**568 – 583 1804.8042 1803.7969 1803.9142 -65.0 0 K.ALAYIEDDELVEVTPK.S**

Spot ID: 51*

**Aspartyl-tRNA synthetase [*Bradyrhizobium* sp. YR681] Mass: 66949 Score: 174 Calculated pI: 5.71 Mass values searched: 41 Mass values matched: 21 Protein sequence coverage: 40%**

**Start – End Observed Mr(expt) Mr(calc) ppm M Peptide**

**23 – 29 854.4100 853.4027 853.4559 -62.3 0 R.LSGWVHR.V**

**30 – 42 1496.7220 1495.7148 1495.8260 -74.3 1 R.VRDHGGVLFIDLR.D**

**130 – 135 819.4305 818.4233 818.4763 -64.7 1 R.FLDLRR.E**

**155 – 178 2642.1152 2641.1080 2641.2595 -57.4 1 R.RMEGQGFFEFNTPILTASSPEGAR.D**

**156 – 178 2486.0095 2485.0022 2485.1584 -62.8 0 R.MEGQGFFEFNTPILTASSPEGAR.D**

**191 – 202 1453.6603 1452.6530 1452.7401 -60.0 0 K.FYALPQAPQQYK.Q**

**203 – 212 1197.5251 1196.5179 1196.5682 -42.1 0 K.QLLMMSGFDR.Y**

**213 – 226 1813.7325 1812.7253 1812.8253 -55.2 1 R.YFQIAPCFRDEDPR.A**

**276 – 284 1072.5619 1071.5546 1071.6189 -60.0 1 R.RIPFAEALR.K**

**277 – 284 916.4954 915.4881 915.5178 -32.4 0 R.IPFAEALR.K**

**286 – 307 2633.1060 2632.0987 2632.2340 -51.4 1 K.YGSDKPDLRNPIEMQDVSEHFR.G**

**295 – 307 1601.6611 1600.6539 1600.7304 -47.8 0 R.NPIEMQDVSEHFR.G**

**317 – 336 2122.9346 2121.9273 2122.0629 -63.9 1 R.MLEDPKNQVWAIPAPGGGSR.A**

**342 – 360 2180.8838 2179.8765 2179.9931 -53.5 0 R.MNSWAQGEGQPGLGYIMWR.E**

**342 – 360 2196.8655 2195.8582 2195.9881 -59.1 0 R.MNSWAQGEGQPGLGYIMWR.E + Oxidation (M)**

**361 – 377 1637.6907 1636.6834 1636.7805 -59.3 0 R.EGGEGAGPLANNIGPER.T**

**389 – 405 1899.7701 1898.7629 1898.8839 -63.8 1 K.EGDAAFFVAGDPDKFWK.F**

**501 – 515 1682.7024 1681.6951 1681.7948 -59.2 0 K.AFEIAGYGEQEVVDR.F**

**501 – 521 2393.9883 2392.9810 2393.1110 -54.3 1 K.AFEIAGYGEQEVVDRFGGMYR.A**

**522 – 539 1801.7609 1800.7536 1800.8366 -46.1 0 R.AFQYGAPPHGGMAAGVDR.I**

**540 – 551 1390.8206 1389.8133 1389.7472 47.5 0 R.IVMLLCGTTNLR.E**

Spot ID: 52**

**Aspartyl-tRNA synthetase [*Bradyrhizobium japonicum* USDA 110]** **Mass:** 66989    **Score:** 63     **Matches:** 1(1)  **Sequences:** 1(1)

Protein sequence coverage: 3% **Monoisotopic mass of neutral peptide Mr(calc):** 2485.1584

**Fixed modifications:** Carbamidomethyl (C)

**Matches :** 75/448 fragment ions using 80 most intense peaks

**Start – End Observed Mr(expt) Mr(calc) ppm M Peptide**

**156 – 178 2485.8572 2484.8499 2485.1584 -124 0 R.MEGQGFFEFNTPILTASSPEGAR.D**

Spot ID: 53*

**ABC transporter ATP-binding protein [*Bradyrhizobium japonicum* USDA 110] Mass: 61850 Score: 199 Calculated pI: 5.58 Mass values searched: 45 Mass values matched: 22**

**Protein sequence coverage: 49%**

**Start – End Observed Mr(expt) Mr(calc) ppm M Peptide**

**2 – 14 1602.8486 1601.8414 1601.8388 1.59 1 M.ARQFIYFMQGLTK.S**

**4 – 14 1375.7158 1374.7085 1374.7006 5.78 0 R.QFIYFMQGLTK.S**

**20 – 34 1759.9551 1758.9478 1758.9305 9.87 1 R.KVLDNIHLSFYPDAK.I**

**21 – 34 1631.8451 1630.8378 1630.8355 1.42 0 K.VLDNIHLSFYPDAK.I**

**21 – 45 2613.2769 2612.2696 2612.3962 -48.5 1 K.VLDNIHLSFYPDAKIGVLGVNGSGK.S**

**51 – 70 2179.0576 2178.0503 2178.0527 -1.11 1 K.IMAGLDKEYNGEAWVAQGAR.V**

**51 – 70 2195.0532 2194.0459 2194.0477 -0.78 1 K.IMAGLDKEYNGEAWVAQGAR.V + Oxidation (M)**

**71 – 83 1489.7814 1488.7741 1488.7460 18.9 0 R.VGYLEQEPQLDAK.L**

**104 – 121 2139.1772 2138.1700 2137.8819 135 0 R.YNELAMNYSEETADEMTK.L**

**231 – 245 1740.8615 1739.8542 1739.8519 1.33 0 K.GIPYEGNYSSWLVQK.Q**

**249 – 261 1510.7603 1509.7530 1509.7171 23.7 1 R.LEQEGREDAAHQK.T**

**266 – 275 1160.5690 1159.5617 1159.5509 9.28 0 R.EQEWVASSPK.A**

**285 – 293 1241.6602 1240.6529 1240.6451 6.24 1 R.YQRYEELLK.Q**

**294 – 312 2111.1411 2110.1338 2110.1382 -2.07 1 K.QASEKQTQTAQIIIPVAER.L**

**299 – 312 1567.8710 1566.8637 1566.8729 -5.90 0 K.QTQTAQIIIPVAER.L**

**313 – 325 1348.7329 1347.7256 1347.7034 16.5 0 R.LGANVVDFEGLSK.G**

**366 – 389 2630.2805 2629.2732 2629.2984 -9.57 1 K.QETPDKGTITVGETVHLGYVDQSR.D**

**455 – 475 2270.1365 2269.1292 2269.1438 -6.42 0 K.SGANVLLLDEPTNDLDVDTLR.A**

**476 – 495 2216.0557 2215.0484 2215.0579 -4.30 0 R.ALEEALEDFAGCAVIISHDR.W**

**501 – 528 3325.5271 3324.5198 3324.5364 -4.97 1 R.IATHILAFEGDSHVEWFEGNFQDYEKDK.M**

**531 – 541 1291.7272 1290.7199 1290.7157 3.29 1 R.RLGQDSIIPHR.V**

**532 – 541 1135.6293 1134.6220 1134.6145 6.56 0 R.LGQDSIIPHR.V**

Spot ID: 54**

**Aspartyl/glutamyl-tRNA amidotransferase subunit A [*Mesorhizobium loti* MAFF303099]** **Mass:** 55801    **Score:** 128    **Matches:** 1(1)**Sequences:** 1(1)

**Protein sequence coverage:** 3% **Monoisotopic mass of neutral peptide Mr(calc):** 2139.1000

**Fixed modifications:** Carbamidomethyl (C)

**Matches :** 76/334 fragment ions using 56 most intense peaks

**Start – End Observed Mr(expt) Mr(calc) ppm M Peptide**

**337 – 356 2139.9562 2138.9489 2139.1000 -70.6 0 K.YALPAYYIVAPAEASSNLAR.Y**

###

Spot ID: 55*

**Elongation factor Tu [*Bradyrhizobium japonicum* USDA 110] Mass: 43569 Score: 130**

**Calculated pI:5.78 Mass values searched: 51 Mass values matched: 16**

**Protein sequence coverage: 35%**

**Start – End Observed Mr(expt) Mr(calc) ppm M Peptide**

**74 – 90 2038.9314 2037.9241 2037.9228 0.66 1 K.NRHYAHVDCPGHADYVK.N**

**76 – 90 1768.8024 1767.7951 1767.7787 9.25 0 R.HYAHVDCPGHADYVK.N**

**91 – 117 2715.3013 2714.2940 2714.3190 -9.21 0 K.NMITGAAQMDGAILVVSAADGPMPQTR.E**

**91 – 117 2731.2837 2730.2764 2730.3139 -13.7 0 K.NMITGAAQMDGAILVVSAADGPMPQTR.E + Oxidation (M)**

**91 – 117 2747.2908 2746.2835 2746.3088 -9.22 0 K.NMITGAAQMDGAILVVSAADGPMPQTR.E + 2 Oxidation (M)**

**118 – 124 851.4969 850.4896 850.5025 -15.1 0 R.EHILLAR.Q**

**125 – 137 1383.8185 1382.8112 1382.8286 -12.6 0 R.QVGVPALVVFLNK.C**

**161 – 172 1419.7736 1418.7663 1418.7809 -10.3 1 K.YEFPGDKIPIIK.G**

**186 – 196 1267.6764 1266.6691 1266.6754 -4.97 0 K.LGHDAILELMR.N**

**186 – 196 1283.6648 1282.6575 1282.6703 -9.99 0 K.LGHDAILELMR.N + Oxidation (M)**

**241 – 251 1213.6628 1212.6556 1212.6714 -13.0 0 K.VGEEIEIVGLR.A**

**256 – 265 1140.5605 1139.5533 1139.5645 -9.87 0 K.TTVTGVEMFR.K**

**256 – 266 1268.6493 1267.6420 1267.6595 -13.8 1 K.TTVTGVEMFRK.L**

**266 – 282 1781.9858 1780.9786 1780.9795 -0.54 1 R.KLLDQGQAGDNIGALLR.G**

**267 – 282 1653.8876 1652.8803 1652.8846 -2.58 0 K.LLDQGQAGDNIGALLR.G**

**322 – 336 2020.9855 2019.9782 2019.9744 1.87 0 R.HTPFFTNYRPQFYFR.T**

Spot ID: 56*

**Elongation factor Tu [*Bradyrhizobium japonicum* USDA 110] Mass: 43569 Score: 129**

**Calculated pI: 5.78 Mass values searched: 45 Mass values matched: 15**

**Protein sequence coverage: 41%**

**Start – End Observed Mr(expt) Mr(calc) ppm M Peptide**

**9 – 25 1883.9435 1882.9362 1882.9221 7.51 0 R.NKPHCNIGTIGHVDHGK.T**

**74 – 90 2038.9833 2037.9760 2037.9228 26.1 1 K.NRHYAHVDCPGHADYVK.N**

**76 – 90 1768.8370 1767.8298 1767.7787 28.9 0 R.HYAHVDCPGHADYVK.N**

**91 – 117 2715.4004 2714.3931 2714.3190 27.3 0 K.NMITGAAQMDGAILVVSAADGPMPQTR.E**

**91 – 117 2731.3613 2730.3541 2730.3139 14.7 0 K.NMITGAAQMDGAILVVSAADGPMPQTR.E + Oxidation (M)**

**118 – 124 851.5648 850.5575 850.5025 64.7 0 R.EHILLAR.Q**

**125 – 137 1383.8676 1382.8603 1382.8286 22.9 0 R.QVGVPALVVFLNK.C**

**138 – 155 2174.0884 2173.0811 2173.0283 24.3 0 K.CDMVDDPELLELVELEVR.E**

**161 – 172 1419.8303 1418.8230 1418.7809 29.7 1 K.YEFPGDKIPIIK.G**

**185 – 196 1395.8234 1394.8161 1394.7704 32.8 1 K.KLGHDAILELMR.N**

**186 – 196 1267.7179 1266.7106 1266.6754 27.8 0 K.LGHDAILELMR.N**

**241 – 251 1213.7124 1212.7051 1212.6714 27.8 0 K.VGEEIEIVGLR.A**

**266 – 282 1782.0261 1781.0188 1780.9795 22.1 1 R.KLLDQGQAGDNIGALLR.G**

**267 – 282 1653.9344 1652.9272 1652.8846 25.8 0 K.LLDQGQAGDNIGALLR.G**

**322 – 336 2021.0447 2020.0374 2019.9744 31.2 0 R.HTPFFTNYRPQFYFR.T**

Spot ID: 57*

**Elongation factor Tu [*Bradyrhizobium japonicum* USDA 110]** **Mass: 43569 Score: 126**

**Calculated pI: 5.78 Mass values searched: 31 Mass values matched: 13**

**Protein sequence coverage: 37%**

**Start – End Observed Mr(expt) Mr(calc) ppm M Peptide**

**74 – 90 2038.9323 2037.9250 2037.9228 1.08 1 K.NRHYAHVDCPGHADYVK.N**

**91 – 117 2715.2932 2714.2859 2714.3190 -12.2 0 K.NMITGAAQMDGAILVVSAADGPMPQTR.E**

**91 – 117 2731.2964 2730.2891 2730.3139 -9.08 0 K.NMITGAAQMDGAILVVSAADGPMPQTR.E + Oxidation (M)**

**118 – 124 851.4911 850.4838 850.5025 -21.9 0 R.EHILLAR.Q**

**125 – 137 1383.8088 1382.8016 1382.8286 -19.5 0 R.QVGVPALVVFLNK.C**

**138 – 155 2174.0037 2172.9964 2173.0283 -14.7 0 K.CDMVDDPELLELVELEVR.E**

**161 – 172 1419.7681 1418.7608 1418.7809 -14.2 1 K.YEFPGDKIPIIK.G**

**186 – 196 1267.6697 1266.6624 1266.6754 -10.3 0 K.LGHDAILELMR.N**

**186 – 196 1283.6884 1282.6811 1282.6703 8.38 0 K.LGHDAILELMR.N + Oxidation (M)**

**241 – 251 1213.6594 1212.6521 1212.6714 -15.9 0 K.VGEEIEIVGLR.A**

**266 – 282 1781.9625 1780.9552 1780.9795 -13.6 1 R.KLLDQGQAGDNIGALLR.G**

**267 – 282 1653.8666 1652.8593 1652.8846 -15.3 0 K.LLDQGQAGDNIGALLR.G**

**322 – 336 2020.9594 2019.9521 2019.9744 -11.1 0 R.HTPFFTNYRPQFYFR.T**

Spot ID: 58*

**Elongation factor Tu [*Bradyrhizobium japonicum* USDA 110 Mass: 43569 Score: 126**

**Calculated pI: 5.78 Mass values searched: 31 Mass values matched: 13**

**Protein sequence coverage: 37%**

**Start – End Observed Mr(expt) Mr(calc) ppm M Peptide**

**74 – 90 2038.9323 2037.9250 2037.9228 1.08 1 K.NRHYAHVDCPGHADYVK.N**

**91 – 117 2715.2932 2714.2859 2714.3190 -12.2 0 K.NMITGAAQMDGAILVVSAADGPMPQTR.E**

**91 – 117 2731.2964 2730.2891 2730.3139 -9.08 0 K.NMITGAAQMDGAILVVSAADGPMPQTR.E + Oxidation (M)**

**118 – 124 851.4911 850.4838 850.5025 -21.9 0 R.EHILLAR.Q**

**125 – 137 1383.8088 1382.8016 1382.8286 -19.5 0 R.QVGVPALVVFLNK.C**

**138 – 155 2174.0037 2172.9964 2173.0283 -14.7 0 K.CDMVDDPELLELVELEVR.E**

**161 – 172 1419.7681 1418.7608 1418.7809 -14.2 1 K.YEFPGDKIPIIK.G**

**186 – 196 1267.6697 1266.6624 1266.6754 -10.3 0 K.LGHDAILELMR.N**

**186 – 196 1283.6884 1282.6811 1282.6703 8.38 0 K.LGHDAILELMR.N + Oxidation (M)**

**241 – 251 1213.6594 1212.6521 1212.6714 -15.9 0 K.VGEEIEIVGLR.A**

**266 – 282 1781.9625 1780.9552 1780.9795 -13.6 1 R.KLLDQGQAGDNIGALLR.G**

**267 – 282 1653.8666 1652.8593 1652.8846 -15.3 0 K.LLDQGQAGDNIGALLR.G**

**322 – 336 2020.9594 2019.9521 2019.9744 -11.1 0 R.HTPFFTNYRPQFYFR.T**

Spot ID: 59**

**ATP phosphoribosyltransferase [*Bradyrhizobium japonicum* USDA 110]** **Mass:** 41078    **Score:** 76     **Matches:** 1(1)  **Sequences:** 1(1)

**Protein sequence coverage:** 3% **Monoisotopic mass of neutral peptide Mr(calc):** 1565.7991

**Fixed modifications:** Carbamidomethyl (C)

**Matches :** 37/246 fragment ions using 40 most intense peaks

**Start – End Observed Mr(expt) Mr(calc) ppm M Peptide**

**85 – 99 1566.6651 1565.6578 1565.7991 -90.2 0 R.AGQPAGFSYLGPVFR.Y**

### Spot ID: 60*

**Elongation factor Ts [*Bradyrhizobium japonicum* USDA 110] Mass: 32175 Score: 82 Calculated pI: 6.17 Mass values searched: 26 Mass values matched: 9**

**Protein sequence coverage: 37%**

**Start – End Observed Mr(expt) Mr(calc) ppm M Peptide**

**25 – 42 2004.6384 2003.6312 2003.9007 -134 0 K.AALTENDGNMEAAQDWLR.K**

**25 – 42 2020.6508 2019.6435 2019.8956 -125 0 K.AALTENDGNMEAAQDWLR.K + Oxidation (M)**

**25 – 43 2132.7529 2131.7457 2131.9956 -117 1 K.AALTENDGNMEAAQDWLRK.K**

**25 – 43 2148.7544 2147.7471 2147.9905 -113 1 K.AALTENDGNMEAAQDWLRK.K + Oxidation (M)**

**139 – 164 2607.0815 2606.0743 2606.3565 -108 1 R.RAASLEVSQGVVSHYVHGAVIDGAGK.M**

**140 – 164 2450.9517 2449.9444 2450.2554 -127 0 R.AASLEVSQGVVSHYVHGAVIDGAGK.M**

**165 – 185 2096.8831 2095.8758 2096.1299 -121 1 K.MGVIVALESPGKADELAALGR.Q**

**186 – 211 2654.1838 2653.1766 2653.4374 -98.3 1 R.QIAMHVAAANPLALDPSGLDPAVVKR.E**

**283 – 306 2440.9070 2439.8997 2440.1758 -113 1 R.YALGEGIEKQESDFAAEVAAASGK.K**

Spot ID: 61**

**Sigma-54 modulation protein [*Bradyrhizobium japonicum* USDA 110]**   **Mass:** 21727    **Score:** 55     **Matches:** 1(1)  **Sequences:** 1(1)

Protein sequence coverage: 7% **Monoisotopic mass of neutral peptide Mr(calc):** 1523.7481

**Fixed modifications:** Carbamidomethyl (C)

**Matches :** 33/261 fragment ions using 36 most intense peaks

**Start – End Observed Mr(expt) Mr(calc) ppm M Peptide**

**182 – 196 1524.6670 1523.6597 1523.7481 -58.0 1 R.RADGNVGWVDPPGGK.A**

###

Spot ID: 62**

**50S ribosomal protein L9** **[*Bradyrhizobium japonicum* USDA 110]**    **Mass:** 21886    **Score:** 152    **Matches:** 1(1)  **Sequences:** 1(1)

Protein sequence coverage: 7% **Monoisotopic mass of neutral peptide Mr(calc):** 1561.7849

**Fixed modifications:** Carbamidomethyl (C)

**Matches :** 72/271 fragment ions using 52 most intense peaks

**Start – End Observed Mr(expt) Mr(calc) ppm M Peptide**

**82 – 96 1562.7817 1561.7744 1561.7849 -6.69 0 R.QASEAGQLFGSVNVR.D**

###

Spot ID: 63**

**ACP S-malonyltransferase [*Bradyrhizobium japonicum* USDA 110] Mass:** 18616    **Score:** 41     **Matches:** 1(1)  **Sequences:** 1(1)

Protein sequence coverage: 7% **Monoisotopic mass of neutral peptide Mr(calc):** 1476.6588

**Fixed modifications:** Carbamidomethyl (C)

**Matches :** 32/206 fragment ions using 47 most intense peaks

**Start – End Observed Mr(expt) Mr(calc) ppm M Peptide**

**93 – 105 1477.4695 1476.4622 1476.6588 -133 0 R.VEELEEGPSAMMR.K**

Spot ID: 64*

**30S ribosomal protein S1 [*Bradyrhizobium japonicum* USDA 6] Mass: 62737 Score: 115**

**Calculated pI: 5.27 Mass values searched: 17 Mass values matched: 10**

**Protein sequence coverage: 25%**

**Start – End Observed Mr(expt) Mr(calc) ppm M Peptide**

**2 – 36 3653.2136 3652.2063 3652.6475 -121 1 M.ASTSADTYSPSRDDFAAMLDESFAGGNLQESSVVK.G**

**145 – 153 1083.4734 1082.4661 1082.6196 -142 0 R.SQVDIRPIR.D**

**183 – 189 847.4111 846.4038 846.4447 -48.3 0 R.TVLEETR.A**

**183 – 193 1331.5098 1330.5025 1330.6841 -136 1 R.TVLEETRAEQR.Q**

**190 – 211 2480.9893 2479.9820 2480.2871 -123 1 R.AEQRQELVQNLEEGQVIDGVVK.N**

**238 – 253 1777.7712 1776.7640 1776.9846 -124 1 R.RVNHPTEVLSIGQTVK.V**

**332 – 350 2087.8384 2086.8311 2087.0998 -129 0 K.IVSTSQEVEVQVLEVDSVK.R**

**332 – 351 2243.9307 2242.9234 2243.2009 -124 1 K.IVSTSQEVEVQVLEVDSVKR.R**

**363 – 383 2295.7832 2294.7759 2295.1172 -149 0 R.NPWEVFVEGHPTGSVVEGEVK.N**

**428 – 439 1371.5675 1370.5602 1370.7405 -132 1 K.AVVLDVDVEKER.I**

Spot ID: 65**

**30S ribosomal protein S1 [*Bradyrhizobium japonicum* USDA 110]**   **Mass:** 64213    **Score:** 61     **Matches:** 1(1)  **Sequences:** 1(1)

Protein sequence coverage: 2% **Monoisotopic mass of neutral peptide Mr(calc):** 1368.7402

**Fixed modifications:** Carbamidomethyl (C) (apply to specified residues or termini only)

**Ions Score:** 61 **Expect:** 0.0044

**Matches :** 38/181 fragment ions using 44 most intense peaks

**Start – End Observed Mr(expt) Mr(calc) ppm M Peptide**

**494 – 505 1369.6271 1368.6198 1368.7402 -87.9 1 K.IAGTDFTTFIKR.S**

### Spot ID: 66*

**30S ribosomal protein S1 [*Bradyrhizobium japonicum* USDA 110] Mass: 64213 Score: 126 Calculated pI: 5.27 Mass values searched: 35 Mass values matched: 15**

**Protein sequence coverage: 30%**

**Start – End Observed Mr(expt) Mr(calc) ppm M Peptide**

**159 – 167 1083.6101 1082.6028 1082.6196 -15.5 0 R.SQVDIRPIR.D**

**168 – 186 2232.9731 2231.9659 2232.1031 -61.5 1 R.DVAPLMNNSQPFQILKMDR.R + Oxidation (M)**

**197 – 207 1331.6448 1330.6375 1330.6841 -35.0 1 R.TVLEETRAEQR.Q**

**204 – 225 2481.1265 2480.1192 2480.2871 -67.7 1 R.AEQRQELVQNLEEGQVIDGVVK.N**

**252 – 267 1777.9186 1776.9113 1776.9846 -41.3 1 R.RVNHPTEVLSIGQTVK.V**

**253 – 267 1621.7988 1620.7916 1620.8835 -56.7 0 R.VNHPTEVLSIGQTVK.V**

**273 – 279 906.4771 905.4698 905.4467 25.5 0 K.INHETHR.I**

**346 – 365 2244.0674 2243.0601 2243.2009 -62.8 1 K.IVSTSQEVEVQVLEVDSVKR.R**

**425 – 435 1275.5852 1274.5779 1274.6507 -57.1 0 K.LPGEQVIDNYK.K**

**437 – 451 1632.7975 1631.7902 1631.8440 -33.0 1 K.GDMVKAVVLDVDVEK.E + Oxidation (M)**

**442 – 453 1371.6929 1370.6856 1370.7405 -40.1 1 K.AVVLDVDVEKER.I**

**475 – 493 2045.9701 2044.9628 2045.0715 -53.1 1 K.GAVVTCEVLEVKESGIEVK.I**

**494 – 505 1369.7048 1368.6976 1368.7402 -31.1 1 K.IAGTDFTTFIKR.S**

**520 – 529 1091.5659 1090.5586 1090.5771 -16.9 1 R.FAVGEKVDAR.V**

**530 – 536 877.5051 876.4979 876.5069 -10.3 1 R.VIQFDKK.A**

Spot ID: 67*

**30S ribosomal protein S1 [*Bradyrhizobium japonicum* USDA 110] Calculated pI: 5.91**

**Mass values searched: 45 Mass values searched: 63 Mass values matched: 35**

**Protein sequence coverage: 57%**

**Start – End Observed Mr(expt) Mr(calc) ppm M Peptide**

**59 – 72 1503.7609 1502.7536 1502.7763 -15.1 1 K.DMAVIDVGLKTEGR.V**

**73 – 83 1188.6370 1187.6297 1187.6411 -9.61 1 R.VALREFSGPGR.D**

**77 – 83 749.3694 748.3621 748.3504 15.6 0 R.EFSGPGR.D**

**84 – 99 1849.9036 1848.8963 1848.9105 -7.70 1 R.DSEIKVGDEVEVFLDR.I**

**100 – 111 1271.6863 1270.6790 1270.6881 -7.14 0 R.IENALGEAVLSR.D**

**159 – 167 1083.6207 1082.6135 1082.6196 -5.71 0 R.SQVDIRPIR.D**

**159 – 183 2879.5171 2878.5098 2878.5487 -13.5 1 R.SQVDIRPIRDVAPLMNNSQPFQILK.M**

**159 – 183 2895.5117 2894.5044 2894.5436 -13.5 1 R.SQVDIRPIRDVAPLMNNSQPFQILK.M + Oxidation (M)**

**168 – 183 1814.9291 1813.9218 1813.9396 -9.83 0 R.DVAPLMNNSQPFQILK.M**

**168 – 183 1830.9341 1829.9268 1829.9346 -4.23 0 R.DVAPLMNNSQPFQILK.M + Oxidation (M)**

**189 – 195 744.4387 743.4314 743.4290 3.31 0 R.GNIVVSR.R**

**189 – 196 900.5246 899.5173 899.5301 -14.2 1 R.GNIVVSRR.T**

**197 – 207 1331.6782 1330.6709 1330.6841 -9.86 1 R.TVLEETRAEQR.Q**

**204 – 225 2481.2620 2480.2547 2480.2871 -13.1 1 R.AEQRQELVQNLEEGQVIDGVVK.N**

**208 – 225 1997.0237 1996.0164 1996.0477 -15.7 0 R.QELVQNLEEGQVIDGVVK.N**

**226 – 251 2831.4006 2830.3934 2830.4290 -12.6 0 K.NITDYGAFVDLGGIDGLLHVTDIAWR.R**

**252 – 267 1777.9713 1776.9640 1776.9846 -11.6 1 R.RVNHPTEVLSIGQTVK.V**

**253 – 267 1621.8574 1620.8501 1620.8835 -20.6 0 R.VNHPTEVLSIGQTVK.V**

**273 – 279 906.4697 905.4624 905.4467 17.3 0 K.INHETHR.I**

**286 – 298 1512.7579 1511.7507 1511.7620 -7.50 0 K.QLLDDPWQGIEAK.Y**

**346 – 364 2088.0735 2087.0662 2087.0998 -16.1 0 K.IVSTSQEVEVQVLEVDSVK.R**

**346 – 365 2244.1738 2243.1666 2243.2009 -15.3 1 K.IVSTSQEVEVQVLEVDSVKR.R**

**373 – 397 2812.3376 2811.3304 2811.3650 -12.3 1 K.QTMRNPWEVFVEGHPTGSVVEGEVK.N**

**373 – 397 2828.3394 2827.3321 2827.3600 -9.86 1 K.QTMRNPWEVFVEGHPTGSVVEGEVK.N + Oxidation (M)**

**377 – 397 2296.0967 2295.0894 2295.1172 -12.1 0 R.NPWEVFVEGHPTGSVVEGEVK.N**

**442 – 453 1371.7228 1370.7155 1370.7405 -18.3 1 K.AVVLDVDVEKER.I**

**460 – 474 1629.7930 1628.7857 1628.8046 -11.6 1 K.QLEGDPFAEPGDVKK.G**

**475 – 486 1303.6779 1302.6706 1302.6853 -11.3 0 K.GAVVTCEVLEVK.E**

**475 – 493 2046.0511 2045.0439 2045.0715 -13.5 1 K.GAVVTCEVLEVKESGIEVK.I**

**494 – 504 1213.6331 1212.6258 1212.6391 -10.9 0 K.IAGTDFTTFIK.R**

**494 – 505 1369.7366 1368.7293 1368.7402 -7.94 1 K.IAGTDFTTFIKR.S**

**511 – 516 803.3864 802.3791 802.3682 13.7 1 R.DRNDQR.A**

**520 – 529 1091.5760 1090.5688 1090.5771 -7.64 1 R.FAVGEKVDAR.V**

**530 – 536 877.4981 876.4908 876.5069 -18.3 1 R.VIQFDKK.A**

**546 – 577 3207.5940 3206.5867 3206.6194 -10.2 1 K.ALEVAEEKEAIAQYGSSDSGATLGDILGTALK.N**

Spot ID: 68**

**Transcription elongation factor GreA [*Bradyrhizobium japonicum* USDA 110]** **Mass:** 17169    **Score:** 95     **Matches:** 1(1)  **Sequences:** 1(1)

Protein sequence coverage: 15% **Monoisotopic mass of neutral peptide Mr(calc):** 2694.1654

**Fixed modifications:** Carbamidomethyl (C)

**Matches :** 44/448 fragment ions using 59 most intense peaks

**Start – End Observed Mr(expt) Mr(calc) ppm M Peptide**

**38 – 61 2694.9858 2693.9785 2694.1654 -69.4 1 R.SHGDLSENAEYHAAKEEQSHNEGR.I**

Spot ID: 69**

**Transcription termination factor Rho [Bradyrhizobium japonicum USDA 110]** **Mass:** 47121    **Score:** 101    **Matches:** 1(1)  **Sequences:** 1(1)

**Protein sequence coverage:** 3% **Monoisotopic mass of neutral peptide Mr(calc):** 1673.8526

**Fixed modifications:** Carbamidomethyl (C)

**Matches :** 29/242 fragment ions using 30 most intense peaks

**Start – End Observed Mr(expt) Mr(calc) ppm M Peptide**

**134 – 147 1674.6422 1673.6349 1673.8526 -130 0 K.VNFDNLTPLFPNQR.F**

Spot ID: 70**

**DNA-directed RNA polymerase alpha subunit [*Bradyrhizobium japonicum* USDA 6**] **Mass:** 38082    **Score:** 77     **Matches:** 1(1)  **Sequences:** 1(1)

Protein sequence coverage: 3% **Monoisotopic mass of neutral peptide Mr(calc):** 1244.6765

**Fixed modifications:** Carbamidomethyl (C)

**Matches :** 13/169 fragment ions using 12 most intense peaks

**Start – End Observed Mr(expt) Mr(calc) ppm M Peptide**

**31 – 41 1245.5515 1244.5442 1244.6765 -106 0 R.FATIVAEPLER.G**

Spot ID: 71**

**DNA-directed RNA polymerase alpha subunit [*Bradyrhizobium japonicum* USDA 110]** **Mass:** 38035    **Score:** 77     **Matches:** 1(1)  **Sequences:** 1(1)  rpoA gene product

**Protein sequence coverage:** 3% **Monoisotopic mass of neutral peptide Mr(calc):** 1244.6765

**Fixed modifications:** Carbamidomethyl (C)

**Matches:** 13/169 fragment ions using 12 most intense peaks

**Start – End Observed Mr(expt) Mr(calc) ppm M Peptide**

**31 – 41 1245.5515 1244.5442 1244.6765 -106 0 R.FATIVAEPLER.G**

Spot ID: 72**

**Hypothetical protein bll4752 [*Bradyrhizobium japonicum* USDA 110]** **Mass:** 27960    **Score:** 77     **Matches:** 1(1)  **Sequences:** 1(1)

Protein sequence coverage: 4% **Monoisotopic mass of neutral peptide Mr(calc):** 1233.6176

**Fixed modifications:** Carbamidomethyl (C)

**Matches:** 42/161 fragment ions using 43 most intense peaks

**Start – End Observed Mr(expt) Mr(calc) ppm M Peptide**

**91 – 101 1234.4706 1233.4633 1233.6176 -125 0 R.TAGDLAWLMTR.E**

Spot ID: 73**

**ATP-dependent protease ATP-binding subunit [*Bradyrhizobium japonicum* USDA 110]** **Mass:** 96620    **Score:** 157    **Matches:** 1(1)  **Sequences:** 1(1)

Protein sequence coverage: 2% **Monoisotopic mass of neutral peptide Mr(calc):** 2204.1113

**Fixed modifications:** Carbamidomethyl (C)

**Matches:** 48/378 fragment ions using 43 most intense peaks

**Start – End Observed Mr(expt) Mr(calc) ppm M Peptide**

**647 – 668 2204.7958 2203.7885 2204.1113 -146 0 R.LIGAPPGYVGYDEGGALTEAVR.R**

Spot ID: 74*

**Heat shock protein [*Bradyrhizobium* sp. WM9] Mass: 65113 Score: 143 Calculated pI: 5.27 Number of mass values searched: 8 Number of mass values matched: 7**

**Protein Sequence Coverage: 14%**

**Start - End Observed Mr(expt) Mr(calc) ppm M Peptide**

**138 - 151 1564.9870 1563.9797 1563.8045 112 0**

**K.AVITVPAYFNDAQR.Q**

**231 - 241 1338.9280 1337.9207 1337.7595 121 1**

**R.LVEYLVAEFKK.D**

**298 - 310 1485.9540 1484.9467 1484.8198 85 1**

**R.AKLESLVDDLVQR.T**

**321 - 340 1989.1740 1988.1667 1987.9885 90 0**

**K.DAGVTAAEIDEVVLVGGMSR.M**

**321 - 340 2005.1500 2004.1427 2003.9834 80 0**

**K.DAGVTAAEIDEVVLVGGMSR.M (OxidationM)**

**418 - 434 1841.0080 1840.0007 1839.8599 77 0**

**K.SQTFSTADDNQSAVTIR.V**

**448 - 462 1593.1270 1592.1197 1591.9086 133 0**

**K.LLGQFDLVGLPPAPR.G**

Spot ID: 75*

**Heat shock protein 90 [*Bradyrhizobium japonicum* USDA 110] Mass: 69004 Score: 96 Calculated pI: 5.08 Mass values searched: 16 Mass values matched: 9**

**Protein sequence coverage: 23%**

**Start – End Observed Mr(expt) Mr(calc) ppm M Peptide**

**146 – 167 2341.8508 2340.8436 2341.0723 -97.7 1 R.RAGESDVWSWTSSGGSGFEIAR.A**

**193 – 201 1215.4774 1214.4701 1214.5819 -92.0 0 K.YLETYEIER.I**

**202 – 224 2557.0405 2556.0333 2556.3111 -109 0 R.IVGAYSDNILFPIELVPEEGEPR.Q**

**225 – 235 1273.5244 1272.5171 1272.6575 -110 0 R.QINSASALWQR.S**

**303 – 317 1707.7035 1706.6962 1706.8879 -112 0 R.VFITDDADLLPGYLR.F**

**335 – 347 1582.6831 1581.6758 1581.8297 -97.3 0 R.EMLQNNPQLVQIR.K**

**354 – 371 1948.7721 1947.7648 1947.9789 -110 1 R.VVSELEGLAEKDAENFAK.I**

**412 – 434 2684.0608 2683.0535 2683.3493 -110 0 K.QVVADFKPNQTEIYYLVGDSIER.L**

**476 – 489 1540.6620 1539.6547 1539.8257 -111 0 K.SLSQGDLNLDLIPR.V**

Spot ID: 76*

**Heat shock protein 90 [*Bradyrhizobium japonicum* USDA 110] Mass: 69004 Score: 87**

**Calculated pI: 5.08 Mass values searched: 11 Mass values matched: 7**

**Protein sequence coverage: 17%**

**Start – End Observed Mr(expt) Mr(calc) ppm M Peptide**

**146 – 167 2341.7664 2340.7591 2341.0723 -134 1 R.RAGESDVWSWTSSGGSGFEIAR.A**

**147 – 167 2185.6938 2184.6866 2184.9712 -130 0 R.AGESDVWSWTSSGGSGFEIAR.A**

**202 – 224 2556.9863 2555.9791 2556.3111 -130 0 R.IVGAYSDNILFPIELVPEEGEPR.Q**

**236 – 246 1256.4441 1255.4368 1255.5932 -125 1 R.SKSELSAEDYK.K**

**248 – 266 2197.7368 2196.7295 2197.0262 -135 0 K.AYQQIASAFDDPAMTLHYR.A**

**335 – 347 1582.5999 1581.5926 1581.8297 -150 0 R.EMLQNNPQLVQIR.K**

**412 – 434 2683.9915 2682.9842 2683.3493 -136 0 K.QVVADFKPNQTEIYYLVGDSIER.L**

Spot ID: 77*

**Molecular chaperone GroEL [*Bradyrhizobium japonicum* USDA 110]** **Mass:** 57749    **Score:** 43     **Matches:** 1(1)  **Sequences:** 1(1)

**Protein sequence coverage**: 2% **Monoisotopic mass of neutral peptide Mr(calc):** 1866.8669

**Fixed modifications:** Carbamidomethyl (C)

**Matches :** 8/276 fragment ions using 18 most intense peaks

**Start – End Observed Mr(expt) Mr(calc) ppm M Peptide**

**182 – 197 1867.6041 1866.5968 1866.8669 -145 0 K.SLETELDVVEGMQFDR.G**

Spot ID: 78*

**Molecular chaperone GroEL [*Bradyrhizobium japonicum* USDA 110] Mass: 57716 Score: 211** **Calculated pI: 5.45 Mass values searched: 46 Mass values matched: 27**

**Protein sequence coverage: 54%**

**Start – End Observed Mr(expt) Mr(calc) ppm M Peptide**

**8 – 13 694.3486 693.3413 693.3446 -4.75 0 K.FSVDAR.D**

**59 – 65 875.4409 874.4336 874.4283 5.99 0 K.EIELEDK.F**

**59 – 75 2038.9520 2037.9447 2037.9499 -2.54 1 K.EIELEDKFENMGAQMVR.E**

**59 – 75 2054.9565 2053.9493 2053.9448 2.16 1 K.EIELEDKFENMGAQMVR.E + Oxidation (M)**

**81 – 101 1988.9926 1987.9853 1988.0175 -16.2 0 K.SADAAGDGTTTATVLAQAIVR.E**

**106 – 118 1389.6495 1388.6423 1388.6904 -34.7 1 K.SVAAGMNPMDLKR.G**

**118 – 133 1667.8990 1666.8918 1666.9618 -42.0 1 K.RGIDLAVEAVVADLVK.N**

**119 – 133 1511.8038 1510.7966 1510.8607 -42.4 0 R.GIDLAVEAVVADLVK.N**

**182 – 197 1867.8442 1866.8370 1866.8669 -16.1 0 K.SLETELDVVEGMQFDR.G**

**182 – 197 1883.8501 1882.8428 1882.8619 -10.1 0 K.SLETELDVVEGMQFDR.G + Oxidation (M)**

**198 – 210 1474.6705 1473.6633 1473.7140 -34.4 0 R.GYISPYFVTNADK.M**

**211 – 225 1839.8384 1838.8311 1838.8906 -32.4 1 K.MRVEMDDAYILINEK.K**

**211 – 225 1855.8591 1854.8519 1854.8855 -18.2 1 K.MRVEMDDAYILINEK.K + Oxidation (M)**

**278 – 284 719.3360 718.3287 718.3398 -15.5 0 K.APGFGDR.R**

**287 – 308 2257.2065 2256.1993 2256.2035 -1.88 0 K.AMLQDIAILTGGQAISEDLGIK.L**

**309 – 319 1259.6384 1258.6312 1258.6703 -31.1 0 K.LENVTLNMLGR.A**

**351 – 364 1684.7217 1683.7144 1683.7587 -26.3 1 K.AQIEETTSDYDREK.L**

**372 – 380 855.5404 854.5331 854.5338 -0.77 0 K.LAGGVAVIR.V**

**381 – 392 1273.6412 1272.6340 1272.6674 -26.3 1 R.VGGATEVEVKER.K**

**394 – 404 1286.5768 1285.5695 1285.5833 -10.8 1 K.DRVDDAMHATR.A**

**405 – 421 1607.8652 1606.8580 1606.9043 -28.8 0 R.AAVEEGIVPGGGVALLR.A**

**438 – 444 773.4655 772.4582 772.4443 18.0 0 K.TGVEIVR.K**

**446 – 452 685.4178 684.4105 684.3918 27.3 0 K.ALSAPAR.Q**

**474 – 492 2156.0232 2155.0159 2155.0110 2.30 0 K.TYAYGFDSQTGEYVNLVTK.G**

**503 – 527 2526.3950 2525.3877 2525.3774 4.09 0 R.TAIQNAASVAALLITTEAMVAELPK.K**

**503 – 527 2542.3755 2541.3682 2541.3723 -1.63 0 R.TAIQNAASVAALLITTEAMVAELPK.K + Oxidation (M)**

**528 – 546 1691.7026 1690.6954 1690.7266 -18.5 1 K.KGGAGPAMPPGGGMGGMDF.-**

Spot ID: 79*

**Trigger factor [*Bradyrhizobium japonicum* USDA 110] Mass: 50061 Score: 94**

**Calculated pI: 4.87 Mass values searched: 27 Mass values matched: 11**

**Protein sequence coverage: 32%**

**Start – End Observed Mr(expt) Mr(calc) ppm M Peptide**

**61 – 82 2554.9060 2553.8987 2554.2333 -131 1 R.SVMAETIDQTIRDTNTQLFSER.G**

**61 – 82 2570.8989 2569.8916 2570.2282 -131 1 R.SVMAETIDQTIRDTNTQLFSER.G + Oxidation (M)**

**92 – 108 1876.7080 1875.7007 1875.9135 -113 0 K.ITMPSEQAEVEELLSGK.T + Oxidation (M)**

**130 – 151 2445.9321 2444.9249 2445.2387 -128 1 K.TFQVEKPVADVTDADVDEAIKR.I**

**236 – 264 3078.0654 3077.0582 3077.4717 -134 0 K.LAGQPAEFETTATSIEAPQDIAIDDEFAK.T**

**282 – 291 1034.4321 1033.4249 1033.5556 -127 0 R.LVAEFAGATR.Q**

**336 – 352 1990.6110 1989.6037 1989.8439 -121 1 K.TFADEDTTEDAAKEEYR.K**

**359 – 371 1412.6769 1411.6696 1411.8398 -121 1 R.VRLGLVLSEIGEK.N**

**396 – 404 1287.4355 1286.4283 1286.5931 -128 1 R.EKEVWDYYR.S**

**398 – 404 1030.3447 1029.3375 1029.4556 -115 0 K.EVWDYYR.S**

**405 – 414 1071.4529 1070.4456 1070.5832 -129 0 R.SNAQALAQLR.A**

Spot ID: 80*

**Trigger factor [*Bradyrhizobium japonicum* USDA 110] Mass: 50061 Score: 106**

**Calculated pI: 4.87 Mass values searched: 20 Mass values matched: 11**

**Protein sequence coverage: 30%**

**Start – End Observed Mr(expt) Mr(calc) ppm M Peptide**

**61 – 82 2554.9897 2553.9825 2554.2333 -98.2 1 R.SVMAETIDQTIRDTNTQLFSER.G**

**61 – 82 2570.9705 2569.9632 2570.2282 -103 1 R.SVMAETIDQTIRDTNTQLFSER.G + Oxidation (M)**

**92 – 108 1876.7562 1875.7490 1875.9135 -87.7 0 K.ITMPSEQAEVEELLSGK.T + Oxidation (M)**

**130 – 151 2445.9971 2444.9898 2445.2387 -102 1 K.TFQVEKPVADVTDADVDEAIKR.I**

**236 – 264 3078.1511 3077.1438 3077.4717 -107 0 K.LAGQPAEFETTATSIEAPQDIAIDDEFAK.T**

**280 – 291 1319.5493 1318.5420 1318.6993 -119 1 R.ERLVAEFAGATR.Q**

**282 – 291 1034.4656 1033.4583 1033.5556 -94.2 0 R.LVAEFAGATR.Q**

**336 – 352 1990.6609 1989.6536 1989.8439 -95.6 1 K.TFADEDTTEDAAKEEYR.K**

**396 – 404 1287.4855 1286.4782 1286.5931 -89.3 1 R.EKEVWDYYR.S**

**398 – 404 1030.3870 1029.3797 1029.4556 -73.7 0 K.EVWDYYR.S**

**405 – 414 1071.4954 1070.4881 1070.5832 -88.9 0 R.SNAQALAQLR.A**

Spot ID: 81*

**ATP-dependent protease ATP-binding subunit ClpX [*Bradyrhizobium japonicum* USDA 110] Mass: 46932 Score: 152 Calculated pI: 5.57 Mass values searched: 20 Mass values matched: 13**

**Protein sequence coverage: 35%**

**Start – End Observed Mr(expt) Mr(calc) ppm M Peptide**

**72 – 84 1444.7478 1443.7405 1443.7358 3.28 0 K.VLDDYVIGQSHAK.K**

**125 – 133 986.6042 985.5970 985.5920 5.03 0 K.TLLAQTLAR.I**

**164 – 174 1291.6559 1290.6486 1290.6568 -6.33 0 K.LLQAADYNVER.A**

**178 – 190 1520.8206 1519.8133 1519.8246 -7.44 1 R.GIVYIDEIDKISR.K**

**213 – 227 1498.7633 1497.7560 1497.7610 -3.30 0 K.IMEGTVASVPPQGGR.K**

**263 – 278 1647.8296 1646.8223 1646.8264 -2.48 0 R.STSIGFGAQVLAPEDR.R**

**263 – 279 1803.9204 1802.9131 1802.9275 -7.97 1 R.STSIGFGAQVLAPEDRR.T**

**286 – 304 2211.0828 2210.0755 2210.1735 -44.4 1 R.HVEPEDLLKYGLIPEFVGR.L**

**295 – 304 1150.6270 1149.6197 1149.6182 1.25 0 K.YGLIPEFVGR.L**

**305 – 321 1871.0332 1870.0259 1870.0299 -2.12 1 R.LPVVATLEDLDETSLKK.I**

**337 – 357 2339.1189 2338.1116 2338.1515 -17.0 0 R.LFEMENIELTFADEALGAVAR.K**

**337 – 357 2355.1367 2354.1294 2354.1464 -7.19 0 R.LFEMENIELTFADEALGAVAR.K + Oxidation (M)**

**398 – 413 1851.9359 1850.9286 1850.9526 -13.0 0 R.EVVEGTARPLYIYADR.S**

Spot ID: 82**

**Anti-oxidant protein [*Bradyrhizobium japonicum* USDA 110]** **Mass:** 24420    **Score:** 59     **Matches:** 1(1)  **Sequences:** 1(1)

**Protein sequence coverage:** 5% **Monoisotopic mass of neutral peptide Mr(calc):** 1248.6900

**Fixed modifications:** Carbamidomethyl (C)

**Matches:** 43/152 fragment ions using 47 most intense peaks

**Start – End Observed Mr(expt) Mr(calc) ppm M Peptide**

**144 – 154 1249.6488 1248.6415 1248.6900 -38.8 0 K.LVLVYPMTTGR.N**

Spot ID: 83**

**Hypothetical protein BJ6T_08050 [*Bradyrhizobium japonicum* USDA 6]** **Mass:** 20515    **Score:** 97     **Matches:** 1(1)  **Sequences:** 1(1)

**Protein sequence coverage**: 6% **Monoisotopic mass of neutral peptide Mr(calc):** 1408.6657

**Fixed modifications:** Carbamidomethyl (C)

**Matches:** 37/204 fragment ions using 35 most intense peaks

**Start – End Observed Mr(expt) Mr(calc) ppm M Peptide**

**21 – 33 1409.6777 1408.6704 1408.6657 3.37 0 R.VVVDGSPMEFSSR.E**

Spot ID: 84**

**Heat shock protein GrpE** **[*Bradyrhizobium japonicum* USDA 110] Mass:** 21642    **Score:** 50     **Matches:** 1(1)  **Sequences:** 1(1)

**Protein sequence coverage:** 4% **Monoisotopic mass of neutral peptide Mr(calc):** 996.5393

**Fixed modifications:** Carbamidomethyl (C)

**Matches:** 40/110 fragment ions using 51 most intense peaks

**Start – End Observed Mr(expt) Mr(calc) ppm M Peptide**

**72 – 80 997.5517 996.5444 996.5393 5.18 0 R.LYGITGFAR.D**

Spot ID: 85**

**Protein-L-isoaspartate O- ethyltransferase [*Bradyrhizobium japonicum* USDA 110] Mass:** 25182    **Score:** 66     **Matches:** 1(1)  **Sequences:** 1(1)

**Protein sequence coverage:** 6% **Monoisotopic mass of neutral peptide Mr(calc):** 1538.8457

**Fixed modifications:** Carbamidomethyl (C)

**Matches:** 70/253 fragment ions using 60 most intense peaks

**Start – End Observed Mr(expt) Mr(calc) ppm M Peptide**

**220 – 234 1539.8822 1538.8749 1538.8457 19.0 0 R.ALFDAAAPVLPGLER.A**

Spot ID: 86*

**Cell division protein FtsZ Mass: 62990 Score: 174 [Bradyrhizobium japonicum USDA 110]**

**Mass: 62990 Calculated pI: 5.21 Values searched: 6 Mass values matched: 5**

**Protein Sequence Coverage: 20%**

**Start - End Observed Mr(expt) Mr(calc) ppm M Peptide**

**39 - 60 2194.3400 2193.3327 2193.1754 72 0**

**R.IIQLGVNVTEGLGAGSQPEVGR.A**

**106 - 123 1914.2440 1913.2367 1913.0524 96 0**

**K.GILTVGVVTKPFHFEGGR.R**

**138 - 152 1729.1520 1728.1447 1727.9570 109 0**

**K.SVDTLIVIPNQNLFR.I**

**251 - 263 1479.9520 1478.9447 1478.7253 148 0**

**R.DLTLFEVDEAATR.I**

**264 - 289 2885.5690 2884.5617 2884.4818 28 1**

**R.IREEVDPDANIILGATFDESLEGIIR.V**

Spot ID: 87**

**Hypothetical protein Blr2761 [*Bradyrhizobium japonicum* USDA 110**] **Mass:** 29257    **Score:** 66     **Matches:** 1(1)  **Sequences:** 1(1)

**Protein sequence coverage:** 3% **Monoisotopic mass of neutral peptide Mr(calc):** 1017.5607

**Fixed modifications:** Carbamidomethyl (C)

**Matches:** 34/113 fragment ions using 39 most intense peaks

**Start – End Observed Mr(expt) Mr(calc) ppm M Peptide**

**69 – 77 1018.5285 1017.5212 1017.5607 -38.8 0 R.LAALEAQFR.A**

Spot ID: 88**

**Two-component response regulator OmpR [*Bradyrhizobium japonicum* USDA 110]** **Mass:** 26233    **Score:** 63     **Matches:** 1(1)  **Sequences:** 1(1)  unnamed protein product

**Protein sequence coverage:** 3% **Monoisotopic mass of neutral peptide Mr(calc):** 1120.5665

**Fixed modifications:** Carbamidomethyl (C)

**Matches:** 23/130 fragment ions using 30 most intense peaks

**Start – End Observed Mr(expt) Mr(calc) ppm M Peptide**

**209 – 217 1121.5336 1120.5263 1120.5665 -35.9 0 R.NFIETVWGR.G**

Spot ID: 89*

**GTP-dependent nucleic acid-binding protein EngD [*Bradyrhizobium japonicum* USDA 110]**

**Mass: 39493 Score: 132 Calculated pI: 5.13 Mass values searched: 17**

**Mass values matched: 11 Protein sequence coverage: 29%**

**Start – End Observed Mr(expt) Mr(calc) ppm M Peptide**

**69 – 79 1203.8114 1202.8041 1202.7023 84.7 0 R.LTFVDIAGLVR.G**

**80 – 96 1732.0320 1731.0247 1730.9064 68.4 1 R.GASKGEGLGNQFLANIR.E**

**84 – 96 1388.8378 1387.8305 1387.7208 79.0 0 K.GEGLGNQFLANIR.E**

**97 – 106 1108.7191 1107.7118 1107.6037 97.7 0 R.EVDAIAHVVR.C**

**242 – 254 1532.8975 1531.8902 1531.7841 69.2 1 K.IESEIATISREER.A**

**255 – 270 1748.9851 1747.9778 1747.8628 65.8 0 R.ADFLETLGLEEAGLDR.L**

**294 – 299 783.4673 782.4600 782.4187 52.7 0 R.AWTIHR.G**

**321 – 339 1926.0276 1925.0203 1924.9167 53.8 0 R.AETIAFDDYVALGGEAGAR.D**

**346 – 362 1965.0680 1964.0607 1963.9462 58.3 1 R.LEGKEYVVADGDVMHFR.F**

**350 – 362 1537.8262 1536.8189 1536.7031 75.3 0 K.EYVVADGDVMHFR.F**

**350 – 362 1553.8043 1552.7970 1552.6980 63.8 0 K.EYVVADGDVMHFR.F + Oxidation (M)**

Spot ID: 90*

**Cobalt insertion protein [*Bradyrhizobium* sp. S23321] Calculated pI: 5.62 Score: 98**

**Nominal mass (Mr): 37304 Mass values searched: 26 Mass values matched: 10**

**Protein sequence coverage: 36%**

**Start – End Observed Mr(expt) Mr(calc) ppm M Peptide**

**2 – 19 1892.7997 1891.7924 1891.9383 -77.1 1 M.TTAAMSKVQEVSGLPDMK.V**

**82 – 91 1111.4222 1110.4150 1110.5782 -147 0 K.STHIEQVAAR.L**

**92 – 98 944.3427 943.3354 943.4698 -142 0 R.LNWPCVR.V**

**108 – 119 1313.5876 1312.5804 1312.7715 -146 1 R.IDLVGKDSIVVR.D**

**176 – 184 1080.4716 1079.4643 1079.6240 -148 0 K.VIKPHPSFR.L**

**185 – 214 3193.1365 3192.1292 3192.5258 -124 0 R.LFATANTVGLGDTSGLYHGTQQINQGQMDR.W**

**185 – 214 3209.0979 3208.0906 3208.5208 -134 0 R.LFATANTVGLGDTSGLYHGTQQINQGQMDR.W + Oxidation (M)**

**261 – 275 1579.5300 1578.5228 1578.7460 -141 0 R.NAFANGDLSTVMSPR.T**

**296 – 316 2626.9653 2625.9581 2626.3213 -138 1 R.VTFLNKCDELERPLVAEFYQR.C**

**302 – 316 1924.6683 1923.6611 1923.9149 -132 0 K.CDELERPLVAEFYQR.C**

Spot ID: 91**

**Dehydrogenase [*Bradyrhizobium japonicum* USDA 110] Mass:** 33290    **Score:** 138    **Matches:** 1(1)  **Sequences:** 1(1)

**Protein sequence coverage:** 5% **Monoisotopic mass of neutral peptide Mr(calc):** 1928.9228

**Fixed modifications:** Carbamidomethyl (C)

**Matches:** 33/316 fragment ions using 32 most intense peaks

**Start – End Observed Mr(expt) Mr(calc) ppm M Peptide**

**224 – 240 1929.9340 1928.9267 1928.9228 2.02 0 R.QTGPQSWDILVETDQGR.M**

Spot ID: 92**

**Hypothetical protein Bll5663 [*Bradyrhizobium japonicum* USDA 110]** **Mass:** 33547    **Score:** 123    **Matches:** 1(1)  **Sequences:** 1(1)  unnamed protein product

**Protein sequence coverage**: 4% **Monoisotopic mass of neutral peptide Mr(calc):** 1439.8136

**Fixed modifications:** Carbamidomethyl (C)

**Matches :** 49/202 fragment ions using 49 most intense peaks

**Start – End Observed Mr(expt) Mr(calc) ppm M Peptide**

**172 – 184 1440.6642 1439.6569 1439.8136 -109 0 K.APYPPIVIITSNR.T**

Spot ID: 93**

**Competence-damage associated protein [*Bradyrhizobium japonicum* USDA 110] Mass:** 26451    **Score:** 75     **Matches:** 1(1)  **Sequences:** 1(1)

**Protein sequence coverage:** 6% **Monoisotopic mass of neutral peptide Mr(calc):** 1602.8267

**Fixed modifications:** Carbamidomethyl (C)

**Matches:** 41/226 fragment ions using 43 most intense peaks

**Start – End Observed Mr(expt) Mr(calc) ppm M Peptide**

**88 – 102 1603.7723 1602.7650 1602.8267 -38.5 0 K.AFGVGIDHHPEVVAR.F**

Spot ID: 94**

**Hypothetical protein Blr5067 [*Bradyrhizobium japonicum* USDA 110] Mass:** 24247    **Score:** 96     **Matches:** 1(1) **Sequences:** 1(1)

**Protein sequence coverage**: 9% **Monoisotopic mass of neutral peptide Mr(calc):** 2182.1780

**Fixed modifications:** Carbamidomethyl (C)

**Matches :** 87/392 fragment ions using 104 most intense peaks

**Start – End Observed Mr(expt) Mr(calc) ppm M Peptide**

**138 – 157 2183.1457 2182.1384 2182.1780 -18.1 1 R.VTVVSTIASQPPMIADELRR.Q**

Spot ID: 95**

**Unknown [*Bradyrhizobium japonicum* USDA 110] Mass:** 16852    **Score:** 75     **Matches:** 1(1)  **Sequences:** 1(1)

Protein sequence coverage: 6% **Monoisotopic mass of neutral peptide Mr(calc):** 1226.6295

**Fixed modifications:** Carbamidomethyl (C)

**Matches:** 45/141 fragment ions using 50 most intense peaks

**Start – End Observed Mr(expt) Mr(calc) ppm M Peptide**

**24 – 33 1227.6068 1226.5995 1226.6295 -24.5 1 K.TLGFDIDYKR.L**

Spot ID: 96*

**Transcription elongation factor NusA [*Bradyrhizobium japonicum* USDA 110] Mass: 59333 Score: 303 Calculated pI: 4.70 Mass values searched: 45 Mass values matched: 28**

**Protein sequence coverage: 56%**

**Start – End Observed Mr(expt) Mr(calc) ppm M Peptide**

**2 – 19 1910.0371 1909.0298 1909.0745 -23.4 1 M.AVSANRLELLQIADAVAR.E**

**8 – 19 1311.7262 1310.7189 1310.7558 -28.1 0 R.LELLQIADAVAR.E**

**26 – 38 1301.6835 1300.6762 1300.7060 -22.9 0 R.GIVIAAMEDAIAK.A**

**42 – 57 1787.8160 1786.8088 1786.8486 -22.3 1 R.ARYGSETDVHAEIDPK.K**

**44 – 57 1560.6959 1559.6886 1559.7104 -13.9 0 R.YGSETDVHAEIDPK.K**

**66 – 87 2546.2510 2545.2437 2545.3071 -24.9 1 R.HMLVVEKVENHSNQISLVDAQR.A**

**73 – 87 1709.8107 1708.8034 1708.8492 -26.8 0 K.VENHSNQISLVDAQR.A**

**88 – 109 2255.0728 2254.0655 2254.1230 -25.5 0 R.ANPGAQVGDTIADTLPPLEYGR.I**

**131 – 136 842.5031 841.4958 841.3970 117 0 R.QYQEFK.D**

**131 – 138 1113.5204 1112.5131 1112.5251 -10.7 1 R.QYQEFKDR.I**

**149 – 161 1462.7660 1461.7587 1461.7940 -24.1 1 K.RVEYGSVIVDLGR.G**

**150 – 167 1946.0256 1945.0184 1945.0633 -23.1 1 R.VEYGSVIVDLGRGEAIIR.R**

**185 – 191 883.4559 882.4486 882.4599 -12.9 0 R.AYIFDVR.R**

**196 – 203 917.5092 916.5019 916.5130 -12.2 0 R.GPQIFLSR.T**

**212 – 228 1962.9912 1961.9839 1962.0350 -26.0 0 K.LFAQEVPEIYDGIVEIK.A**

**246 – 259 1449.6200 1448.6127 1448.6388 -18.0 0 R.DSSVDPVGACVGMR.G**

**309 – 326 1938.0109 1937.0036 1937.0582 -28.2 0 R.IEVVVPDTNNQLSLAIGR.R**

**327 – 332 729.4108 728.4035 728.4042 -0.92 1 R.RGQNVR.L**

**333 – 353 2433.1201 2432.1128 2432.1707 -23.8 0 R.LASQLTGWDIDILTEQEESER.R**

**333 – 354 2589.2112 2588.2039 2588.2718 -26.2 1 R.LASQLTGWDIDILTEQEESERR.Q**

**355 – 363 1067.4679 1066.4606 1066.4679 -6.86 0 R.QADFENSTR.V**

**397 – 415 2148.9473 2147.9400 2147.9971 -26.6 0 K.ELAGIEGFDEETAQELQNR.A**

**416 – 430 1806.8395 1805.8322 1805.8795 -26.2 1 R.AREYLEQQEAELEAK.R**

**418 – 430 1579.7159 1578.7087 1578.7413 -20.7 0 R.EYLEQQEAELEAK.R**

**454 – 479 2897.2568 2896.2496 2896.3185 -23.8 1 K.FGENDIKTVDDLAGCATDDLVGWTER.K**

**461 – 479 2093.9019 2092.8946 2092.9372 -20.4 0 K.TVDDLAGCATDDLVGWTER.K**

**461 – 480 2221.9880 2220.9808 2221.0321 -23.1 1 K.TVDDLAGCATDDLVGWTERK.E**

**513 – 536 2499.0850 2498.0777 2498.1448 -26.9 0 K.AGWITEADLAKPAEEAEASEDQPA.-**

Spot ID: 97*

**ATP-dependent phosphoenolpyruvate carboxykinase [*Bradyrhizobium* sp. YR681] Mass: 59235 Score: 115 Calculated pI: 6.01 Mass values searched: 24 Mass values matched: 10**

**Protein sequence coverage: 22%**

**Start – End Observed Mr(expt) Mr(calc) ppm M Peptide**

**106 – 121 1743.6388 1742.6315 1742.8264 -112 0 K.SLFAQDLYGGADPAYR.I**

**171 – 185 1702.6500 1701.6427 1701.8257 -107 1 R.HGCKSENVVAIDFAR.K**

**175 – 185 1220.4968 1219.4896 1219.6197 -107 0 K.SENVVAIDFAR.K**

**202 – 214 1602.6288 1601.6215 1601.8089 -117 0 K.SVFTTLNYYLPER.G**

**280 – 294 1663.6237 1662.6164 1662.8213 -123 0 K.LSQEAEPQIYAASTR.F**

**295 – 309 1737.6078 1736.6005 1736.8040 -117 0 R.FGAVLENCVLDEDTR.V**

**324 – 336 1450.5579 1449.5506 1449.7252 -120 0 R.SAYPLDFIPNASR.T**

**451 – 462 1200.5488 1199.5416 1199.6874 -122 0 R.ALLTAALDGSLR.N**

**463 – 470 1008.4949 1007.4876 1007.5036 -15.9 1 R.NVEFRTDK.Y**

**514 – 527 1623.6403 1622.6330 1622.8053 -106 1 K.NFAKFEAQVDAEVR.A**

Spot ID: 98*

**ABC transporter substrate-binding protein [*Bradyrhizobium japonicum* USDA 110] Mass: 59019 Score: 151 Calculated pI: 6.93 Mass values searched: 29 Mass values matched: 15**

**Protein sequence coverage: 37%**

**Start – End Observed Mr(expt) Mr(calc) ppm M Peptide**

**37 – 47 1243.5564 1242.5491 1242.6391 -72.4 0 K.TLTAVMHSDLR.I**

**48 – 60 1523.7073 1522.7000 1522.8395 -91.6 0 R.IIDPIFTTAYITR.D**

**61 – 78 2075.7610 2074.7537 2074.9306 -85.3 0 R.DHGYMVYDTLVATDSNFK.I**

**88 – 100 1572.6834 1571.6761 1571.8195 -91.3 1 K.ISDDKLTYTFTLR.D**

**152 – 169 1958.9028 1957.8956 1958.0836 -96.1 1 K.LKEPYGLVLDSIGKPSSR.V**

**198 – 208 1265.5446 1264.5373 1264.6452 -85.3 0 K.FVQSEFQPGVK.A**

**286 – 293 925.4183 924.4110 924.4818 -76.5 0 K.FGFQTLGR.M**

**294 – 305 1484.5979 1483.5906 1483.7170 -85.2 0 R.MNFLYPPFDNVK.V**

**332 – 354 2469.9773 2468.9700 2469.1920 -89.9 0 K.VCGAFFVCDTPLATDVGAETLVK.G**

**388 – 398 1179.6073 1178.6000 1178.7135 -96.3 0 K.AQPIVAAQLLR.E**

**399 – 417 2149.8984 2148.8912 2149.0804 -88.1 1 R.EAGFKVDLQATDWQTVVSR.R**

**404 – 417 1617.6810 1616.6738 1616.8159 -87.9 0 K.VDLQATDWQTVVSR.R**

**494 – 514 2481.0488 2480.0416 2480.2740 -93.7 0 K.EAYEQVIYVPLGQYLLPSGWR.K**

**494 – 515 2609.1284 2608.1211 2608.3689 -95.0 1 K.EAYEQVIYVPLGQYLLPSGWRK.S**

**516 – 536 2231.9397 2230.9324 2231.1110 -80.1 1 K.SLSGVLDGPATPLFWNVDKSE.-**

Spot ID: 99**

**Hypothetical protein Bll0565 [*Bradyrhizobium japonicum* USDA 110] Mass: 41554    Score: 75     Matches: 1(1) Sequences: 1(1)**

**Protein sequence coverage:** 3% **Monoisotopic mass of neutral peptide Mr(calc):** 1397.6687

**Fixed modifications:** Carbamidomethyl (C)

**Matches :** 43/199 fragment ions using 51 most intense peaks

**Start – End Observed Mr(expt) Mr(calc) ppm M Peptide**

**250 – 262 1398.5020 1397.4947 1397.6687 -125 0 R.HGDPYEAQLAAAR.S**

Spot ID: 100*

**TldD protein [*Bradyrhizobium japonicum* USDA 110] Mass: 51101 Score: 86 Calculated pI: 5.51 Mass values searched: 32 Mass values matched: 11**

**Protein sequence coverage: 30%**

**Start – End Observed Mr(expt) Mr(calc) ppm M Peptide**

**2 – 16 1688.7291 1687.7218 1687.7472 -15.0 0 M.DGHMTNPDTTSLLDR.A + Oxidation (M)**

**57 – 71 1684.7759 1683.7686 1683.8580 -53.1 1 R.LKQATYDTSQGFGLR.A**

**59 – 71 1443.6165 1442.6092 1442.6790 -48.4 0 K.QATYDTSQGFGLR.A**

**72 – 93 2284.0193 2283.0120 2283.1859 -76.2 1 R.AVKDDAVGYAHSSDVSLPALIR.A**

**138 – 147 1176.5825 1175.5752 1175.6550 -67.8 0 K.LLAEIDAYLR.D**

**186 – 203 1830.8309 1829.8237 1829.9232 -54.4 1 R.VNISVVAGQGDRQESGSK.G**

**275 – 284 1010.4650 1009.4578 1009.5015 -43.3 0 R.TSAFAGLMGR.Q**

**318 – 333 1821.8447 1820.8375 1820.9342 -53.2 0 R.TVLIEDGILVGYMQDR.Q**

**360 – 380 2295.9949 2294.9876 2295.1239 -59.4 1 R.MTNTYMLAGDRDPAEILASVK.N**

**360 – 380 2311.9792 2310.9720 2311.1188 -63.5 1 R.MTNTYMLAGDRDPAEILASVK.N + Oxidation (M)**

**381 – 399 1838.8405 1837.8332 1837.8959 -34.1 0 K.NGVFAANFGGGQVDITSGK.Y**

Spot ID: 101*

**Hypothetical protein Blr7534 [*Bradyrhizobium japonicum* USDA 110] Mass: 49518 Score: 201 Calculated pI: 5.86 Mass values searched: 27 Mass values matched: 18**

**Protein sequence coverage: 45%**

**Start – End Observed Mr(expt) Mr(calc) ppm M Peptide**

**100 – 120 2358.1184 2357.1111 2357.1573 -19.6 1 R.YFTSIDGLMDGNADVILKETR.Q**

**100 – 120 2374.1274 2373.1202 2373.1522 -13.5 1 R.YFTSIDGLMDGNADVILKETR.Q + Oxidation (M)**

**124 – 138 1606.8402 1605.8329 1605.8436 -6.67 0 K.TVTAAVLDVCYPVAK.N**

**144 – 167 2504.2903 2503.2830 2503.3395 -22.6 1 K.DRFVVNLQVAGQTLTGTTQSLGAK.A**

**146 – 167 2233.1523 2232.1451 2232.2114 -29.7 0 R.FVVNLQVAGQTLTGTTQSLGAK.A**

**177 – 186 1197.5784 1196.5711 1196.5826 -9.61 0 R.KPTGDTFEFR.G**

**240 – 250 1234.7368 1233.7295 1233.6969 26.5 1 K.VKLDAATEFLK.S**

**251 – 272 2346.1833 2345.1761 2345.2261 -21.3 1 K.SLKGQEVEVTLASLTVGCDALR.A**

**254 – 272 2017.9720 2016.9648 2017.0150 -24.9 0 K.GQEVEVTLASLTVGCDALR.A**

**273 – 286 1546.6910 1545.6838 1545.7093 -16.5 0 R.AGEQTINMSVDPER.A**

**273 – 293 2171.0508 2170.0435 2170.1052 -28.4 1 R.AGEQTINMSVDPERAGALLAK.F**

**296 – 314 1952.8363 1951.8290 1951.8590 -15.4 0 K.AMPGVTAAGWTAGMTEMDR.T**

**296 – 314 1968.8312 1967.8239 1967.8540 -15.3 0 K.AMPGVTAAGWTAGMTEMDR.T + Oxidation (M)**

**318 – 324 836.4123 835.4050 835.3977 8.79 0 R.FAAADWR.D**

**332 – 344 1240.7177 1239.7104 1239.7299 -15.8 1 R.DKLAAAVAGVLGR.T**

**334 – 344 997.6165 996.6092 996.6080 1.24 0 K.LAAAVAGVLGR.T**

**345 – 361 1743.9131 1742.9058 1742.9315 -14.8 0 R.TLAAKPVSQNFNPATGK.L**

**419 – 445 2789.1509 2788.1436 2788.2159 -25.9 0 K.LNLSDEASADEEGDQPDDNGSVEALAK.E**

Spot ID: 102**

**ABC transporter substrate-binding protein [*Bradyrhizobium japonicum* USDA 110]** **Mass:** 47774 **Score:** 50 **Matches:** 1(1)  **Sequences:** 1(1)

**Sequence Coverage:** 3% **Monoisotopic mass of neutral peptide Mr(calc):** 1981.9170

**Fixed modifications:** Carbamidomethyl (C)

**Matches:** 21/338 fragment ions using 37 most intense peaks

**Start – End Observed Mr(expt) Mr(calc) ppm M Peptide**

**109 – 126 1983.0860 1982.0787 1981.9170 81.6 0 K.FASGNPVTAEDVAWSFER.A**

Spot ID: 103**

**Fructose 1,6-bisphosphatase II [*Bradyrhizobium japonicum* USDA 110] Mass:** 35603    **Score:** 80     **Matches:** 1(1)  **Sequences:** 1(1)

**Protein sequence coverage:** 3% **Monoisotopic mass of neutral peptide Mr(calc):** 1261.5938

**Fixed modifications:** Carbamidomethyl (C)

**Matches :** 35/165 fragment ions using 28 most intense peaks

**Start – End Observed Mr(expt) Mr(calc) ppm M Peptide**

**256 – 266 1262.5440 1261.5367 1261.5938 -45.3 0 K.AIAEAPDQEYR.E**

Spot ID: 104**

**Substrate-binding protein [*Bradyrhizobium japonicum* USDA 110**] **Mass:** 40839    **Score:** 97     **Matches:** 1(1)  **Sequences:** 1(1)

**Protein sequence coverage:** 3% **Monoisotopic mass of neutral peptide Mr(calc):** 1429.6838

**Fixed modifications:** Carbamidomethyl (C)

**Matches :** 35/203 fragment ions using 34 most intense peaks

**Start – End Observed Mr(expt) Mr(calc) ppm M Peptide**

**281 – 292 1430.6523 1429.6450 1429.6838 -27.1 1 R.GDQYSTIFKDTR.D**

Spot ID: 105**

**Ketol-acid reductoisomerase [*Bradyrhizobium japonicum* USDA 110]** **Mass:** 21727    **Score:** 55     **Matches:** 1(1)  **Sequences:** 1(1)

**Protein sequence coverage:** 7% **Monoisotopic mass of neutral peptide Mr(calc):** 1523.7481

**Fixed modifications:** Carbamidomethyl (C)

**Matches :** 33/261 fragment ions using 36 most intense peaks

**Start – End Observed Mr(expt) Mr(calc) ppm M Peptide**

**182 – 196 1524.6670 1523.6597 1523.7481 -58.0 1 R.RADGNVGWVDPPGGK.A**

Spot ID: 106**

**Dioxygenase [*Bradyrhizobium japonicum* USDA 110] Mass: 34110 Score: 85 Calculated pI: 5.35 Mass values searched: 18 Mass values matched: 6**

**Protein sequence coverage: 42%**

**Start – End Observed Mr(expt) Mr(calc) ppm M Peptide**

**2 – 24 2594.0698 2593.0625 2593.2660 -78.5 1 M.TQFNETELTAAVVESFDKTPNPR.A**

**91 – 110 2204.9385 2203.9312 2204.0862 -70.3 1 R.DREGATQTTVLGPFYVGEHK.V**

**111 – 127 1796.7100 1795.7027 1795.8449 -79.2 0 K.VTAHGTDISPNNQTGER.M**

**139 – 173 3775.5352 3774.5279 3774.7551 -60.2 0 K.GKPLAGVPVDVWHADDDGFYDSQKPNYDEVGASAR.A**

**176 – 187 1507.6129 1506.6056 1506.7256 -79.6 1 R.FITDSDGRFFFR.T**

**188 – 211 2658.1377 2657.1304 2657.3193 -71.1 0 R.TILPCSYPIPTDGPVGEMIVQTNR.H**

Spot ID: 107**

**Hypothetical protein Bll5131 [*Bradyrhizobium japonicum* USDA 110]** **Mass:** 34214    **Score:** 167    **Matches:** 1(1)  **Sequences:** 1(1)

Protein sequence coverage: 5% **Monoisotopic mass of neutral peptide Mr(calc):** 2129.9364

**Fixed modifications:** Carbamidomethyl (C)

**Matches :** 52/326 fragment ions using 38 most intense peaks

**Start – End Observed Mr(expt) Mr(calc) ppm M Peptide**

**87 – 104 2130.8378 2129.8305 2129.9364 -49.7 0 R.ILYNFTGSSCEGYTSEFR.Q**

Spot ID: 108**

**Hypothetical protein Blr2961 [*Bradyrhizobium japonicum* USDA 110]** **Mass:** 25510    **Score:** 52     **Matches:** 1(1)  **Sequences:** 1(1)

Protein sequence coverage: 5% **Monoisotopic mass of neutral peptide Mr(calc):** 1311.6936

**Fixed modifications:** Carbamidomethyl (C)

**Matches:** 28/180 fragment ions using 34 most intense peaks

**Start – End Observed Mr(expt) Mr(calc) ppm M Peptide**

**217 – 228 1312.5789 1311.5716 1311.6936 -93.0 1 R.HGYDVVTLPVRG.-**

Spot ID: 109**

**Hypothetical protein Bll5307 [*Bradyrhizobium japonicum* USDA 110]** **Mass:** 14177    **Score:** 124    **Matches:** 1(1)  **Sequences:** 1(1)

**Protein sequence coverage:** 12% **Monoisotopic mass of neutral peptide Mr(calc):** 1792.8479

**Fixed modifications:** Carbamidomethyl (C)

**Matches :** 39/277 fragment ions using 35 most intense peaks

**Start – End Observed Mr(expt) Mr(calc) ppm M Peptide**

**18 – 33 1793.8242 1792.8169 1792.8479 -17.3 0 R.AEAETLFEINEAATER.T**

Spot ID: 110**

**Hypothetical protein Blr2191 [*Bradyrhizobium japonicum* USDA 110]** **Mass:** 25491    **Score:** 117    **Matches:** 1(1)  **Sequences:** 1(1)

**Protein sequence coverage:** 6% **Monoisotopic mass of neutral peptide Mr(calc):** 1629.7899

**Fixed modifications:** Carbamidomethyl (C)

**Matches :** 50/246 fragment ions using 44 most intense peaks

**Start – End Observed Mr(expt) Mr(calc) ppm M Peptide**

**199 – 213 1630.7445 1629.7372 1629.7899 -32.3 0 R.GPAADAHAIQPYYTR.L**

Spot ID: 111**

**ATP-dependent Clp protease proteolytic subunit [*Bradyrhizobium japonicum* USDA 110]**

**Mass:** 22404    **Score:** 100    **Matches:** 1(1)  **Sequences:** 1(1)

**Protein sequence coverage:** 6% **Monoisotopic mass of neutral peptide Mr(calc):** 1689.8250

**Fixed modifications:** Carbamidomethyl (C)

**Matches :** 63/248 fragment ions using 68 most intense peaks

**Start – End Observed Mr(expt) Mr(calc) ppm M Peptide**

**130 – 144 1690.7773 1689.7700 1689.8250 -32.5 1 R.VFETEAGKYVESFGK.Q**

Spot ID: 112**

**Hypothetical protein Bll7551 [*Bradyrhizobium japonicum* USDA 110]** **Mass:** 27565    **Score:** 135    **Matches:** 1(1)  **Sequences:** 1(1)

**Protein sequence coverage:** 5% **Monoisotopic mass of neutral peptide Mr(calc):** 1403.6681

**Fixed modifications:** Carbamidomethyl (C)

**Matches :** 73/226 fragment ions using 53 most intense peaks

**Start – End Observed Mr(expt) Mr(calc) ppm M Peptide**

**109 – 121 1404.6426 1403.6353 1403.6681 -23.3 0 K.QSEVGEAAWTQAK.S**

Spot ID: 113*

**Hypothetical protein Blr0227 [*Bradyrhizobium japonicum* USDA 110] Mass: 22619 Score: 124 Calculated pI: 5.17 Mass values searched: 21 Mass values matched: 9**

**Protein sequence coverage: 50%**

**Start – End Observed Mr(expt) Mr(calc) ppm M Peptide**

**38 – 48 1271.5972 1270.5899 1270.5717 14.3 0 K.DGEDFLVYDAK.T**

**56 – 68 1518.8654 1517.8581 1517.8089 32.4 0 R.SVLAQIIFEQENK.A**

**69 – 80 1330.8085 1329.8012 1329.7405 45.7 0 K.AGQNLLPTTFLR.Q**

**97 – 111 1780.9298 1779.9225 1779.8890 18.8 0 K.YLEQSIATLTQEQEK.F**

**97 – 113 2084.1553 2083.1480 2083.0585 42.9 1 K.YLEQSIATLTQEQEKFR.K**

**114 – 133 2199.2874 2198.2801 2198.1695 50.3 1 R.KQIANTLSGTPFAPLEEQVR.R**

**115 – 133 2071.1621 2070.1548 2070.0746 38.8 0 K.QIANTLSGTPFAPLEEQVR.R**

**115 – 134 2227.2822 2226.2750 2226.1757 44.6 1 K.QIANTLSGTPFAPLEEQVRR.N**

**155 – 179 2656.2510 2655.2437 2655.2147 10.9 1 R.SSTTPEPEPDATAEAPKDSNIDDLR.Q**

Spot ID: 114**

**Hypothetical protein Blr0227** **[*Bradyrhizobium japonicum* USDA 110]** **Mass:** 22619    **Score:** 136    **Matches:** 1(1)  **Sequences:** 1(1)

**Protein sequence coverage**: 7% **Monoisotopic mass of neutral peptide Mr(calc):** 1779.8890

**Fixed modifications:** Carbamidomethyl (C)

**Matches :** 51/275 fragment ions using 30 most intense peaks

**Start – End Observed Mr(expt) Mr(calc) ppm M Peptide**

**97 – 111 1780.9298 1779.9225 1779.8890 18.8 0 K.YLEQSIATLTQEQEK.F**

Spot ID: 115**

**Hypothetical protein Blr7436 [*Bradyrhizobium japonicum* USDA 110]** **Mass:** 15290    **Score:** 202    **Matches:** 1(1)  **Sequences:** 1(1)

**Protein sequence coverage:** 17% **Monoisotopic mass of neutral peptide Mr(calc):** 2535.2188

**Fixed modifications:** Carbamidomethyl (C)

**Matches :** 67/483 fragment ions using 52 most intense peaks

**Start – End Observed Mr(expt) Mr(calc) ppm M Peptide**

**9 – 32 2536.0244 2535.0171 2535.2188 -79.5 0 K.TETTETETVDSNLAAVTEVEAGIR.D**
