# Supplementary material for: Proteomic analysis of free-living Bradyrhizobium diazoefficiens: highlighting potential determinants of a successful symbiosis
Source: BMC Genomics. 2014 Aug 3;15:643. doi: 10.1186/1471-2164-15-643 (PMC4287336; doi:10.1186/1471-2164-15-643)
Supplement: Supplementary file 3 — Additional file 3: Table S2: Statistical results of RT-qPCR data provided by Rest2009 software package. (DOCX 19 KB) [file 12864_2014_6775_MOESM3_ESM.docx]

**Additional file 3: Table S2 Statistical results of RT-qPCR data provided by Rest2009 software package**

| **Hypotetical protein-coding genes** | **Expression** | **Std. Error** | **95% C.I.** | **P(H1)** | **qPCR Result** |
| --- | --- | --- | --- | --- | --- |
| *nodC* | 2,291 | 1,490 - 3,407 | 1,158 - 4,834 | 0 | Up regulated |
| *bll0565* | 1,82 | 1,440 - 2,214 | 1,248 - 2,570 | 0 | Up regulated |
| *blr7534* | 1,379 | 1,076 - 1,722 | 0,907 - 1,933 | 0,002 | Up regulated |
| *bll5131* | 0,842 | 0,477 - 1,427 | 0,309 - 1,932 | 0,381 |  |
| *blr2961* | 1,681 | 1,280 - 2,151 | 1,042 - 2,684 | 0 | Up regulated |
| *bll5307* | 1,433 | 1,117 - 1,835 | 1,003 - 2,210 | 0,001 | Up regulated |
| *blr2191* | 1,228 | 0,993 - 1,484 | 0,850 - 1,706 | 0,011 | Up regulated |
| *bll7551* | 0,43 | 0,168 - 2,316 | 0,140 - 2,542 | 0,07 |  |
| *blr0227* | 1,294 | 1,068 - 1,599 | 0,870 - 1,879 | 0,003 | Up regulated |
| *blr7436* | 1,247 | 0,772 - 2,149 | 0,519 - 3,159 | 0,204 |  |

**Interpretation:**

| **Interpretation** |
| --- |
|  |
| *bll0565* is UP-regulated in sample group (in comparison to control group) by a mean factor of 1,820 (S.E. range is 1,440 - 2,214). |
| *bll0565* sample group is different to control group. P(H1)=0,000 |
|  |
| *blr7534* is UP-regulated in sample group (in comparison to control group) by a mean factor of 1,379 (S.E. range is 1,076 - 1,722). |
| *bll5131* sample group is different to control group. P(H1)=0,002 |
|  |
| *bll5131* sample group is not different to control group. P(H1)=0,381 |
|  |
| *blr296*1 is UP-regulated in sample group (in comparison to control group) by a mean factor of 1,681 (S.E. range is 1,280 - 2,151). |
| *blr2961* sample group is different to control group. P(H1)=0,000 |
|  |
| *bll5307* is UP-regulated in sample group (in comparison to control group) by a mean factor of 1,433 (S.E. range is 1,117 - 1,835). |
| *bll5307* sample group is different to control group. P(H1)=0,001 |
|  |
| *blr2191* is UP-regulated in sample group (in comparison to control group) by a mean factor of 1,228 (S.E. range is 0,993 - 1,484). |
| *blr2191* sample group is different to control group. P(H1)=0,011 |
|  |
| *bll7551* sample group is not different to control group. P(H1)=0,070 |
|  |
| *blr0227* is UP-regulated in sample group (in comparison to control group) by a mean factor of 1,294 (S.E. range is 1,068 - 1,599). |
| *blr0227* sample group is different to control group. P(H1)=0,003 |
|  |
| *blr7436* sample group is not different to control group. P(H1)=0,204 |
|  |
| NodC is UP-regulated in sample group (in comparison to control group) by a mean factor of 2,291 |
| NodC sample group is different to control group. P(H1)=0 |

**Table S2** **Sequence of the primers used in RT-qPCR and their respective rates of amplification efficiency**

| **Hypothetical protein- coding genes** |  | **Primer Sequences** | **Efficiency** |
| --- | --- | --- | --- |
| *nodC* | F | 5' GTCAAGACTACGCCGGAAAG 3' | 93% |
|  | R | 5' CGACGTTGAGAACCAGATCA 3' |  |
| 16S rRNA | F | 5' CAGCTCGTGTCGTGAGATGT 3' | 97% |
|  | R | 5' CACCGGCAGTCTCCTTAGAG 3' |  |
| *bll0565* | F | 5' AAGGTGTCGAGTGGCTTCAG 3' | 96% |
|  | R | 5' CAGGACGATGGACGACAAG 3' |  |
| *blr7534* | F | 5' GAGACGCGCTATTTCACCTC 3' | 94% |
|  | R | 5' ACCGGATAACAGACGTCGAG 3' |  |
| *bll5131* | F | 5' TGTCCTTGACGTCGAACTTG 3' | 99% |
|  | R | 5' GAGCACATGAAATCGCTCAA 3' |  |
| *blr2961* | F | 5' GAGGTCGAGTTCGTGCTGAT 3' | 91% |
|  | R | 5' TCTGCTTGGAGACTGTGACG 3' |  |
| *bll5307* | F | 5' TACATGCCGATGTCGAACAC 3' | 92% |
|  | R | 5' AAGGTCAACAGCGAGATGCT 3' |  |
| *blr2191* | F | 5' ACGATGAGCTTTCGCATCAC 3' | 97% |
|  | R | 5' CCGCGTATAATAAGGCTGGA 3' |  |
| *bll7551* | F | 5' GACCTCCGACTGCTTCTTCA 3' | 93% |
|  | R | 5' CAATGGTCACGCAAAACAAC 3' |  |
| *blr0227* | F | 5' AAATCAGACCAACCCACCAC 3' | 90% |
|  | R | 5'CCTTGTTCTCCTGCTCGAAG 3' |  |
| *blr7436* | F | 5'AGACGGTCGACAGCAATCTC 3' | 92% |
|  | R | 5' ATCAGCGAGTTGACGTTGGT 3' |  |
